# Understanding and Expanding Zinc Cation/Amine Frustrated Lewis Pair Catalyzed C–H Borylation

**DOI:** 10.1021/acscatal.2c05995

**Published:** 2023-01-30

**Authors:** Matthew E. Grundy, Lia Sotorrios, Milan Kumar Bisai, Kang Yuan, Stuart A. Macgregor, Michael J. Ingleson

**Affiliations:** † EaStCHEM School of Chemistry, University of Edinburgh, Edinburgh, EH9 3FJ, United Kingdom; ‡ Institute of Chemical Sciences, Heriot-Watt University, Edinburgh, EH14 4AS, United Kingdom

**Keywords:** Borylation, Frustrated Lewis Pairs, DFT Calculations, zinc, electrophilic substitution

## Abstract

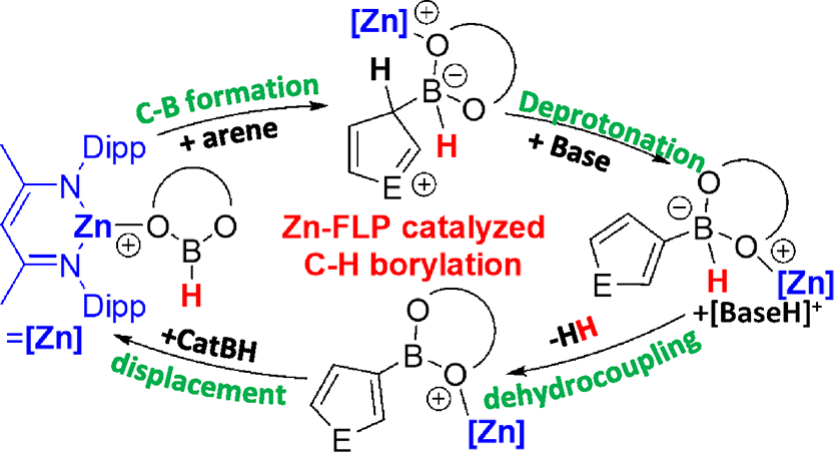

[(NacNac)Zn(DMT)][B(C_6_F_5_)_4_], **1**, (NacNac = {(2,6-^*i*^Pr_2_H_3_C_6_)N(CH_3_)C}_2_CH), DMT = *N,N*-dimethyl-4-toluidine), was synthesized via two routes starting from either (NacNac)ZnEt or (NacNac)ZnH. Complex **1** is an effective (pre)catalyst for the C–H borylation of (hetero)arenes using catecholborane (CatBH) with H_2_ the only byproduct. The scope included weakly activated substrates such as 2-bromothiophene and benzothiophene. Computational studies elucidated a plausible reaction mechanism that has an overall free energy span of 22.4 kcal/mol (for *N*-methylindole borylation), consistent with experimental observations. The calculated mechanism starting from **1** proceeds via the displacement of DMT by CatBH to form [(NacNac)Zn(CatBH)]^+^, **D**, in which CatBH binds via an oxygen to zinc which makes the boron center much more electrophilic based on the energy of the CatB-based LUMO. Combinations of **D** and DMT act as a frustrated Lewis pair (FLP) to effect C–H borylation in a stepwise process via an arenium cation that is deprotonated by DMT. Subsequent B–H/[H-DMT]^+^ dehydrocoupling and displacement from the coordination sphere of zinc of CatBAr by CatBH closes the cycle. The calculations also revealed a possible catalyst decomposition pathway involving hydride transfer from boron to zinc to form (NacNac)ZnH which reacts with CatBH to ultimately form Zn(0). In addition, the key rate-limiting transition states all involve the base, thus fine-tuning of the steric and electronic parameters of the base enabled a further minor enhancement in the C–H borylation activity of the system. Outlining the mechanism for all steps of this FLP-mediated process will facilitate the development of other main group FLP catalysts for C–H borylation and other transformations.

## Introduction

**Figure 1:**
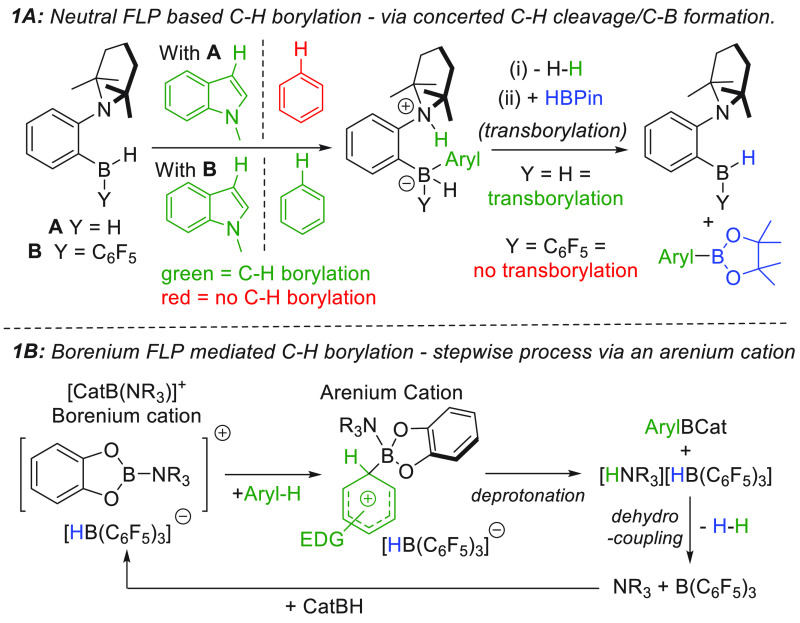
Exemplar catalytic C–H borylation processes using boron electrophiles and bases.

C–H functionalization is an efficient way to install functional groups onto (hetero)aromatics. In this area, one transformation of particular importance is arene C–H borylation,^1^^2^ as this produces synthetically versatile organoboranes.^3^ The most important advance in this area has been iridium-catalyzed C–H borylation;^1^ however, the use of precious metal-based catalysts has drawbacks, with catalysts based on earth abundant elements preferred where possible.^4^ For 3d metal systems, notable advances have been reported using cobalt-based catalysts for C–H borylation.^5^ However, cobalt, like iridium, has a very low permitted daily exposure (PDE) value.^6^ While there are C–H borylation catalysts based on 3d metals with higher PDE values (e.g., Fe, Mn)^7^ these systems are limited in scope and/or require the (hetero) arene substrate to be present in large excess (relative to the borane).^5^ There also have been reports using high PDE main group catalysts for C–H borylation, the majority of which involve a boron electrophile and a base.^3^ To be effective the boron electrophile and base cannot be quenched by the formation of a strong dative bond, thus this represents frustrated Lewis pair (FLP) catalysis.^8^ However, since the seminal report in 2015 on neutral FLP-based C–H borylation catalysis (with compound **A**, Figure 1A),^8,9^ the substrate scope has remained limited to activated (hetero)arenes (more nucleophilic than furan, Mayr *N* value = +1.3).^10^ Main group element based catalytic C–H borylation also can be achieved via borenium cation-mediated processes (e.g., Figure 1B).^11^ However, to date borenium-mediated C–H borylation catalysis also is restricted to highly activated (hetero) arenes.^11^ Therefore, developing main group-based FLP catalysts that have an expanded (hetero)arene C–H borylation scope remains a significant challenge.

Boron electrophile-based C–H borylation generally proceeds via a stepwise or a concerted S_E_Ar type process,^8,12^ and the more electrophilic the borane, the greater the substrate scope amenable to C–H borylation is in terms of (hetero)arene nucleophilicity. For example, the replacement of one H in **A** for a C_6_F_5_ group in **B** (Figure 1, top) enables C–H borylation of nonactivated aromatics such as benzene.^13^ However, with **B**/HBPin this process does not turn over.^8^ Borenium-mediated catalytic borylation processes are restricted in arene scope^14^ due to the electrophilicity at boron being reduced by the presence of the two π donor (OR) groups present in dioxaborolanes (e.g., in [CatB(NR_3_)]^+^ electrophiles).^15^ Therefore, to generate FLP catalysts containing more electrophilic boranes that effect the C–H borylation of less activated arenes and turnover requires a different approach.

**Figure 2:**
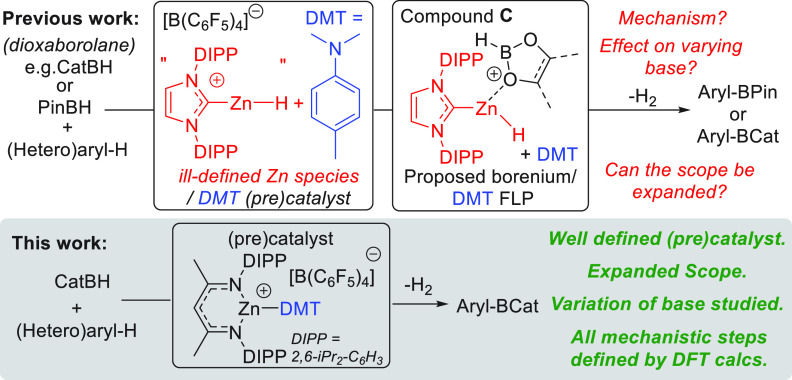
Top: previous work on NHC-Zn cation/DMT catalyzed borylation along with outstanding questions. Bottom: this work with well-defined (NacNac)Zn based electrophiles.

A number of us,^16^ and other groups,^17^ have utilized electrophilic zinc cations to catalyze the borylation of π nucleophiles. As part of this endeavor we reported a zinc-catalyzed C–H borylation process in which the data supported a mechanism proceeding via a cationic zinc electrophile interacting with an oxygen in a dioxaborolane (PinBH or CatBH, compound **C**, Figure 2 inset top).^18^ DFT calculations indicated that binding of a zinc electrophile to the dioxaborolane generated a reactive borenium equivalent (based on the boron-based LUMO energy). In addition, it was found that adding *N,N*-dimethyl-4-toluidine (DMT) as an exogenous base improved the C–H borylation catalysis, indicating that an FLP-type mechanism may be operating.^8,14^ While this study expanded the substrate scope further than previously reported FLP catalyzed borylations, it had limitations, e.g., the activated (toward S_E_Ar) heteroarene thiophene was only borylated in 22% after 36 h at 100 °C using CatBH. Furthermore, after the initial step (the formation of **C**) the subsequent steps were not understood, partly due to the ill-defined nature of the proposed [(IDIPP)Zn–H]^+^ species present in these reactions (IDIPP = 1,3-bis(2,6-diisopropylphenyl) imidazol-2-ylidene). Importantly, the electrophilicity at boron in these systems is linked to the degree of activation of the dioxaborolane on binding to the metal cation (e.g., the strength of the O–Zn interaction in **C**); thus variation in the cationic metal complex will impact the electrophilicity at boron. Therefore, a highly electrophilic zinc complex that would activate a dioxaborolane to a greater extent (than in **C**) should lead to a greater borylation substrate scope.

In this combined experimental–computational study [(NacNac)Zn]^+^ (herein NacNac refers to {(2,6-^*i*^Pr_2_H_3_C_6_)N(CH_3_)C}_2_CH, Figure 2, bottom) based complexes enabled enhanced reactivity in C–H borylation relative to that using **C**, and the generation of a more reactive (in terms of heteroarene nucleophilicity) main group FLP-catalyzed arene C–H borylation system. Furthermore, the well-defined nature of the (pre)catalyst enabled DFT calculations which defined an FLP-mediated stepwise S_E_Ar borylation mechanism that is followed by a dehydrocoupling step between a zinc-coordinated borohydride and an ammonium cation to regenerate the zinc cation and the amine.

## Results and Discussion

### C–H Borylation Catalyzed by [(NacNac)Zn]^+^/DMT

**Figure 3:**
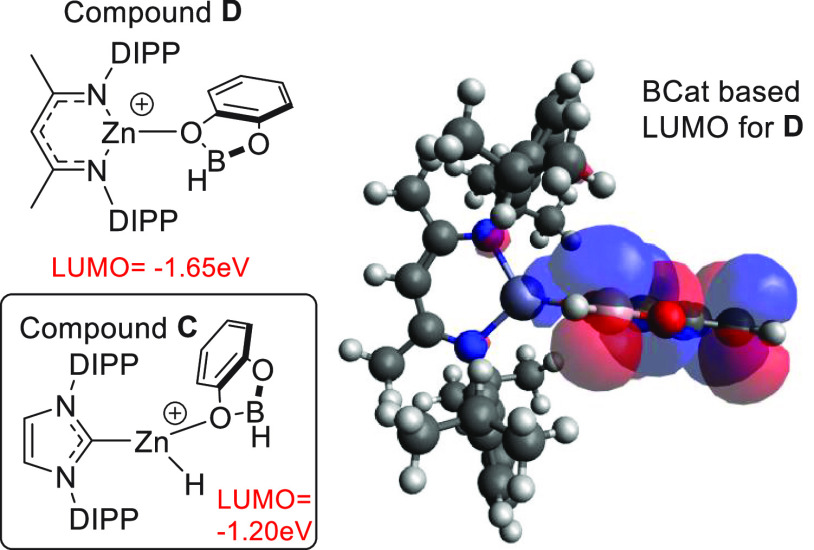
Calculations on the [Zn]···CatBH adducts for cations **C** and **D**. Right: LUMO with significant boron character for **D** (iso surface value = 0.03).

The study commenced by identifying a cationic zinc complex that would coordinate to a dioxaborolane to form a boron-based electrophile with a lower LUMO relative to that in compound **C**. Cognizant of the extremely electrophilic nature of [(NacNac)Zn]^+^ cations (which are more electrophilic than [(IDIPP)Zn-R]^+^ cations),^19^ the CatBH adduct of [(NacNac)Zn]^+^ was calculated at the same computational level used in our previous study.^18^ Notably, this adduct, termed **D**, contains a Zn–O_(CatBH)_ contact (2.14 Å) dramatically shorter than the Zn–O distances calculated for the (IDIPP)Zn(H)-dioxaborolane complexes **C** (in [(IDIPP)Zn(H)]^+^ adducts with PinBH and CatBH the Zn–O distances are 2.36 and 2.59 Å, respectively); presumably the shorter Zn–O distance is due to the higher electrophilicity of the {(NacNac)Zn}^+^ fragment. Furthermore, the LUMO with significant boron character for compound **D** (Figure 3, top) is calculated to be 0.45 eV deeper in energy than the comparable (in character) LUMO calculated for [(IDIPP)ZnH(CatBH)]^+^ (Figure 3, inset). This deeper LUMO with significant boron character suggested that if **D** were accessible it would be a more reactive boron electrophile than **C**.

**Scheme 1:**
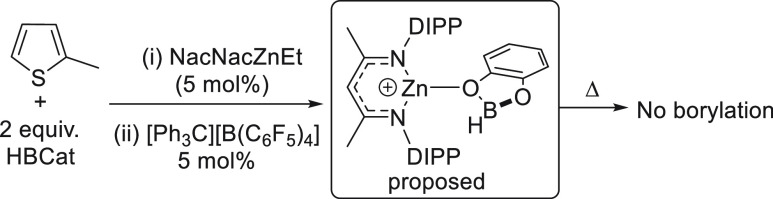
Attempted Borylation under Exogenous Base-Free Conditions

In previous work Dagorne’s procedure was used to isolate [(IDIPP)ZnEt][B(C_6_F_5_)_4_],^20^ with the robust and weakly coordinating [B(C_6_F_5_)_4_]^−^ anion found to maximize C–H borylation yields;^18^ thus this anion is used throughout this study. In an attempt to access a well-defined [(NacNac)Zn]^+^ (pre) catalyst, (NacNac)ZnEt was reacted with [Ph_3_C][B(C_6_F_5_)_4_] in chlorobenzene at room temperature which resulted in competitive formation of Ph_3_CH/ethene and Ph_3_CEt, both consistent with formation of a cationic zinc species. However, the ^1^H NMR spectrum at short reaction times showed multiple NacNac species and no pure material could be isolated from this mixture. This is fully analogous to the work of Schulz and co-workers who performed the same reaction, albeit in CH_2_Cl_2_.^21^ On standing at room temperature in chlorobenzene this mixture undergoes anion decomposition ultimately leading to the formation of B(C_6_F_5_)_3_ and (NacNac)Zn(C_6_F_5_) as the major products (after 18 h by ^19^F NMR spectroscopy, Figure S38). The decomposition of the [B(C_6_F_5_)_4_]^−^ anion is consistent with the considerable electrophilicity expected for the putative [(NacNac)Zn]^+^ species. Adding a species that would coordinate to Zn in preference to the anion would disfavor anion decomposition. Therefore, (NacNac)ZnEt was premixed with a dioxaborolane (PinBH or CatBH) and then [Ph_3_C][B(C_6_F_5_)_4_] was added. While this prevented any significant anion decomposition ([B(C_6_F_5_)_4_]^−^ is the major fluorine containing species (>95%) by ^19^F NMR spectroscopy) it still led to intractable mixtures containing multiple NacNac species (see Figure S39). It should be noted that the reaction using CatBH while still giving multiple species was cleaner than that using PinBH, presumably due to the greater stability of CatBH toward very strong electrophiles.^22^ This observation led to CatBH being utilized hereon. CatBEt was observed in these reactions; however, (NacNac)ZnEt/CatBH metathesis to form CatBEt is slow relative to the reaction of (NacNac)ZnEt with [Ph_3_C][B(C_6_F_5_)_4_] (by *in situ* NMR spectroscopy). Therefore, we attribute the formation of CatBEt to the hydroboration of ethene under these sealed-tube conditions. The minimal anion decomposition observed on activating (NacNac)ZnEt in the presence of CatBH suggested an interaction between the dioxaborolane and [(NacNac)Zn]^+^ (e.g., to form **D**); therefore the activity of this combination in C–H borylation was explored. 2-Methylthiophene was used as it is a relatively activated heteroarene (Mayr nucleophilicity (*N*) parameter = +1.35)^10^ that is challenging to reduce (e.g., to the tetrahydrothiophene). The latter is important to determine the necessity of an exogenous base, as other activated heteroarenes (e.g., indoles) are often reduced *in situ* (to indolines) during catalytic C–H borylation reactions, and these can function as “hidden” Brønsted bases in C–H borylation.^14^ 2-Methylthiophene and CatBH (two equiv) were combined in chlorobenzene and (NacNac)ZnEt (5 mol %) and [Ph_3_C][B(C_6_F_5_)_4_] (5 mol %) then were added. This reaction led to no borylation of 2-methylthiophene even at 80 °C (after 18 h, Scheme 1), suggesting the requirement for an exogenous base in borylations using (NacNac)Zn-based electrophiles.

**Scheme 2:**
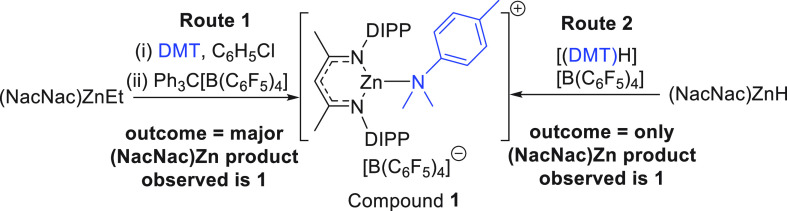
Two Routes Used to Form Compound **1**

Building on our previous report using zinc cations/DMT to catalyze C–H borylation,^18^ the effect of adding *N,N*-dimethyl-4-toluidine (DMT) was explored, with it expected to coordinate to and stabilize a [(NacNac)Zn]^+^ cation, but potentially be displaced by CatBH during a C–H borylation cycle to function as the Brønsted base. Cognizant that [(NacNac)Zn]^+^ can bind one or two equiv of low steric-demand bases (e.g., 4-^*t*^Bu-pyridine),^23^ we combined (NacNac) ZnEt with one and two equiv of DMT before the addition of [Ph_3_C][B(C_6_F_5_)_4_]. The presence of DMT prevented any anion decomposition and led to one major (NacNac)Zn product from each reaction (along with several minor NacNac-containing products); the major (NacNac)Zn products were distinct and thus are attributed to a different speciation of Zn (e.g., one or two equiv of DMT bound to zinc, respectively). As clean material could not be isolated from any of these reactions an alternative route combining [H(DMT)][B(C_6_F_5_)_4_] and (NacNac)ZnH was explored. This combination led to rapid gas evolution (H_2_) and afforded a single (NacNac)Zn product (by *in situ* NMR spectroscopy) with the spectroscopic data fully consistent with [(NacNac)Zn(DMT)][B(C_6_F_5_)_4_], **1**. While compound **1** could not be isolated as a solid it is formed cleanly *in situ* by this route and was fully characterized in C_6_D_5_Br (see Figure S42). Note, the NMR spectra for **1** were identical with the major product formed from the reaction of equimolar (NacNac)ZnEt/DMT and [Ph_3_C][B(C_6_F_5_)_4_] (Figure S43). All studies from hereon use **1** formed *in situ* via either of these routes Scheme 2.

**Chart 1:**
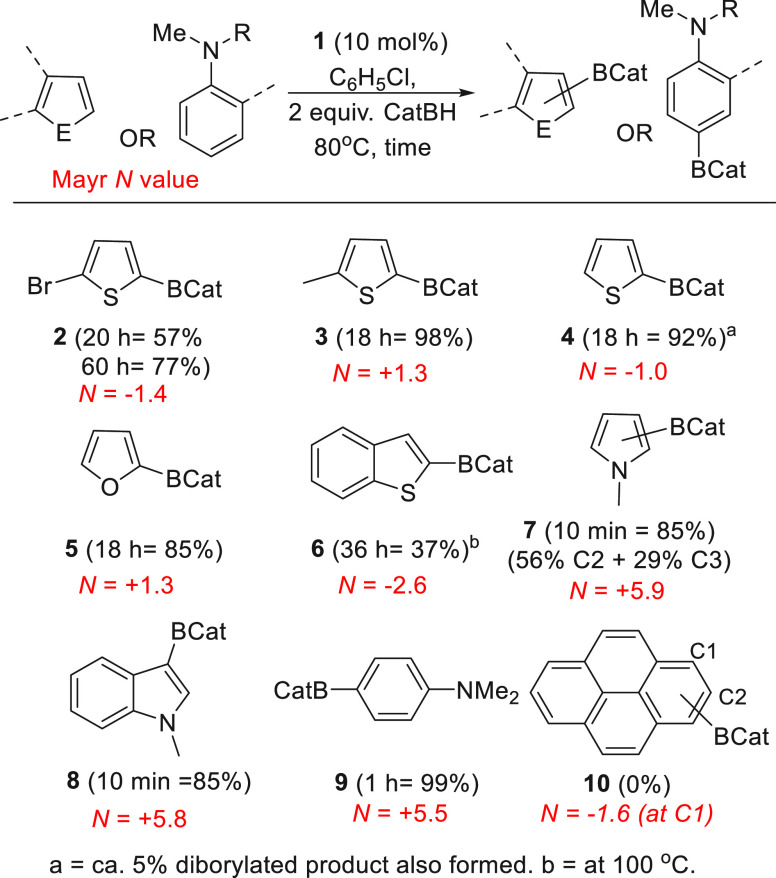
Borylation of (Hetero)arenes Using CatBH Catalyzed by **1**a

With compound **1** accessible cleanly using route 2 we explored its utility as a well-defined (pre) catalyst in the borylation of a weakly activated heteroarene, 2-bromothiophene (Mayr *N* value = −1.4).^10^ The use of **1** (made by route 2) at 10 mol % loading in the borylation of 2-bromothiophene using 2 equiv of CatBH in chlorobenzene led to the formation of **2** in 54% conversion (versus an internal standard) after 20 h at 80 °C. The use of **1** made from 10 mol % (NacNac)ZnEt/DMT and [Ph_3_C][B(C_6_F_5_)_4_] (i.e., made via route 1) led to comparable outcomes for the borylation of 2-bromothiophene under identical conditions (57% conversion, versus an internal standard), consistent with compound **1** being the major (NacNac)Zn species present from route 1. A Zn-based catalyst was essential under these conditions as in the absence of **1** no (or very low yielding) borylation was observed under identical conditions (see Figure S47). The zinc-free control reactions were performed using 10 mol % [Ph_3_C][B(C_6_F_5_)_4_]/DMT and heating to 80 °C (or to 100 °C) with the substrate and 2 equiv CatBH. As complex **1** made via either route gave comparable outcomes in C–H borylations we used route 1 hereon due to (NacNac)ZnEt being simpler to make (and higher yielding in our hands) than the Zn–H congener, combined with route 1 being more readily amenable to variation in base (both the identity and stoichiometry). Indeed, the effect of the equivalents of DMT used (relative to [(NacNac)Zn]^+^) was explored using route 1, and it was found that C–H borylation was retarded using >1 equiv (relative to [Zn]) of DMT). With the effectiveness of **1** for the borylation of weakly activated (hetero)arenes confirmed the scope was probed, particularly to determine the lower limit in terms of (hetero)arene nucleophilicity amenable to this C–H borylation process (Chart 1).

a Made *in situ* via route 1. Conversions are by ^1^H NMR spectroscopy versus an internal standard.

As expected, heteroarenes more nucleophilic than 2-bromothiophene were readily borylated using **1** (e.g., to produce **3** – **5**). Notably, these reactions proceeded at lower temperatures and in higher yields than using our previous catalyst system based on {(IDIPP)Zn} cations. For example, for borylation mediated by **C**, compound **4** was formed in only 22% at 100 °C after 36 h, whereas using compound **1** at the same catalyst loading/concentration **4** was formed in 92% after 18 h at 80 °C. Notably, benzothiophene with a lower Mayr *N* value of −2.6^24^ also was borylated, albeit slowly (37% after 36 h at 100 °C). As expected, much more activated heteroarenes (Mayr *N* values ca. +5 to +6) can be borylated within 10 min at 80 °C (with *N*-methylindole also undergoing borylation at room temperature). Another highly activated substrate, *N,N*-dimethylaniline (Mayr *N* value = +5.5), also was readily borylated in high yield to form compound **9** regioselectively. Finally, during these reactions catalyst decomposition occurs slowly with gray solid (assigned as Zn metal) precipitating. This solid is not catalytically active (based on a control reaction using isolated solid), but its formation means that for slower reactions (e.g., the formation of **6**) conversions plateau and cannot be driven to completion using longer reaction durations.

All substrates discussed thus far are unhindered (hetero)arenes, and for these the substrate scope is predictable using the nucleophilicity scale developed by Mayr and co-workers.^10,24^ Therefore using **1**/CatBH, unhindered (hetero)arenes with a Mayr *N* parameter ≥ *N* = −2.6 (the value for benzothiophene) are expected to be amenable to borylation. This is a notable extension over our previous catalytic system (mediated by (IDIPP)Zn cations) where even thiophene was challenging to borylate. However, it is known that the Mayr scale does not incorporate steric effects; thus hindered electrophiles (such as tritylium derivatives) can show lower reactivity than otherwise predicted, particularly with hindered nucleophiles.^25^ As the boron center in the putative compound **D** is encapsulated by flanking 2,6-diisopropylphenyl units it is a hindered electrophile. Therefore the effect of this steric bulk was probed using pyrene as a nucleophile. Pyrene was chosen as it has a closely comparable Mayr *N* value to that of 2-bromothiophene (pyrene has a *N* value at its most nucleophilic carbon (C1) of −1.6),^24^ but importantly pyrene does not react at C1 in S_E_Ar with bulky electrophiles due to steric hindrance from the peri C–H.^26^ Notably, the use of **1**/ CatBH under conditions that effect the borylation of 2-bromothiophene led to no borylation of pyrene (no formation of **10**, Chart 1), indicating that the proposed adduct **D** is indeed a hindered electrophile at boron which moderates its reactivity with certain hindered nucleophiles. In contrast, less-hindered boron electrophiles do C–H borylate pyrene.^15^

### Computational Studies on the Mechanism of C–H Borylation

Another advantage of using **1** (relative to the precursors to **C**) is that it provides a well-defined and robust (pre)catalyst (no change is observed after heating **1** at 18 h at 80 °C in C_6_D_5_Br). With no reaction observed by NMR spectroscopy on combining **1** separately with 2-bromo-thiophene and with HBCat, and no intermediates observed during the borylation process DFT calculations were used to probe the mechanism. The reaction of **1** with *N*-methylindole was taken as an exemplar process. Calculations used the BP86 functional with geometries and free energies (at 298 K) derived from optimization and frequency calculations in the gas-phase with electronic energies corrected for chlorobenzene solvent, dispersion and basis set effects (see Supporting Materials for full details along with computed geometries of all stationary points).

**Figure 4:**
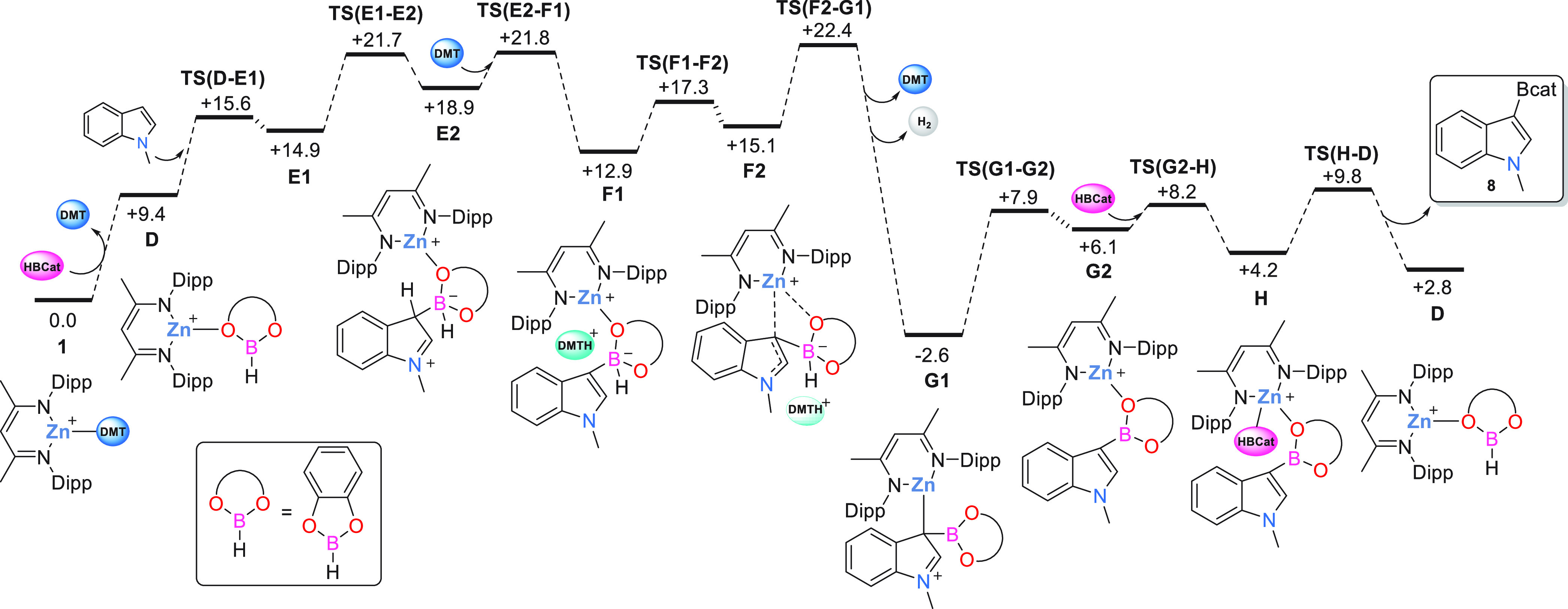
Computed free energy profile (kcal/mol) for C–H borylation of *N*-methylindole. Level of theory: BP86[D3BJ, chlorobenzene]/Def2TZVP//BP86/SDD (Zn); 6-31G** on other atoms.

**Figure 5:**
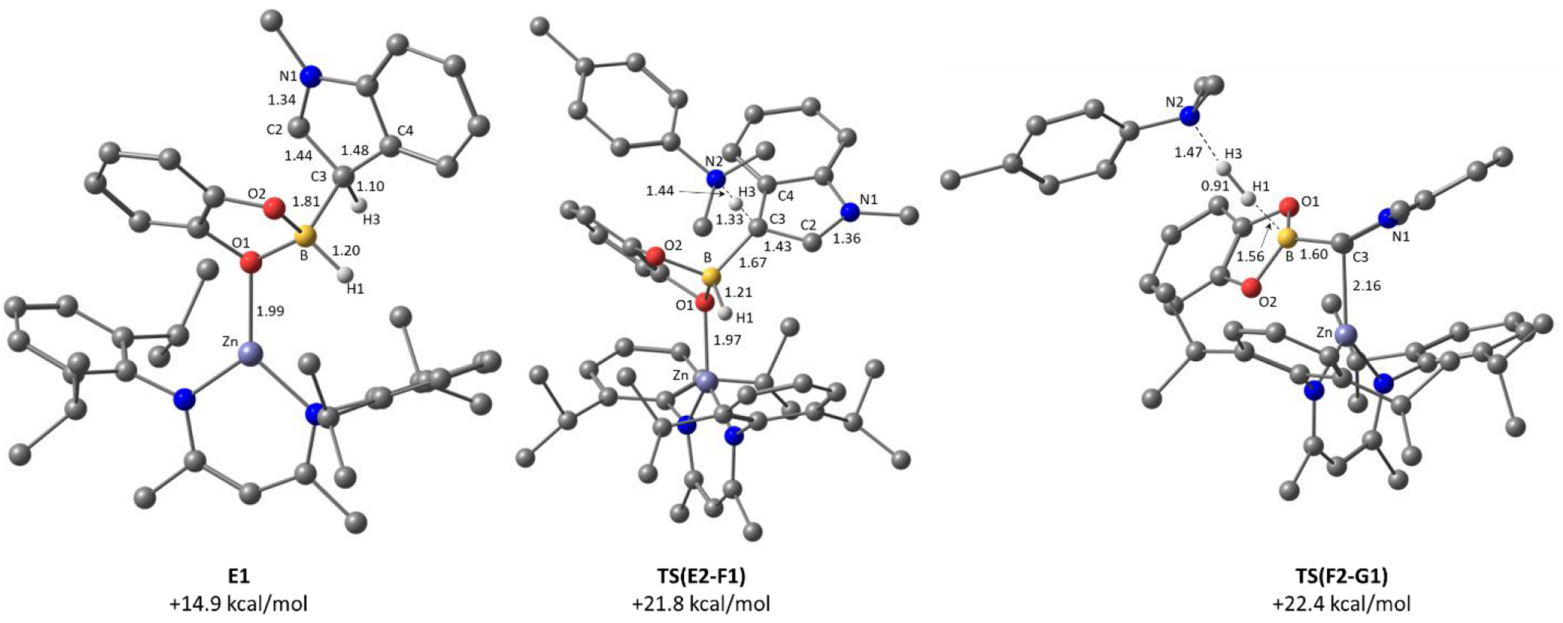
Computed structures of key stationary states highlighting key distances in Å; nonparticipating hydrogens are omitted for clarity.

The computed reaction profile for the C–H borylation of *N*-methylindole is shown in Figure 4 with the geometries of key stationary points in Figure 5. The substitution of the DMT ligand in **1** by CatBH is endergonic and forms intermediate **D** at +9.4 kcal/mol with a computed Zn–O1 distance of 2.05 Å. B–C bond formation then proceeds via **TS(D-E1)** at +15.6 kcal/mol and gives **E1** at +14.9 kcal/mol. **TS(D-E1)** exhibits an extremely early geometry with a B···C3 distance of 3.71 Å and this remains relatively long in **E1** (1.81 Å, Figure 5). **E1** corresponds to an arenium intermediate, with a significant elongation of ca. 0.05 Å computed for both the C2–C3 and the C3–C4 bonds and a similar contraction of the N1–C2 bond compared to the free substrate. The indole C3–H3 bond in **E1** is directed toward the NacNac moiety, and so a ca. 120° rotation about B–C3 via **TS(E1-E2)** is required to form conformer **E2** in which the C3–H3 bond becomes accessible to the external DMT base. Deprotonation of **E2** occurs via **TS(E2-F1)** and gives **F1** in which the [DMT-H]^+^ cation H-bonds to O2 in the BCat moiety (O2···H3 = 1.62 Å). Further rearrangement to isomer **F2** then establishes a B–H^δ−^···H^δ+^–N dihydrogen interaction (H1···H3 = 1.50 Å) as a precursor to dehydrocoupling via **TS(F2-G1)** at +22.4 kcal/mol. As well as loss of H_2_, the dehydrocoupling process from **F1** to **G1** is accompanied by a change in coordination mode of the indole-borate/borane moiety, from O-bound in **F1** (Zn–O1 = 1.96 Å) to C-bound in **G1** (Zn–C3 = 2.13 Å). **F2** shows an intermediate geometry in which both sites interact more weakly with the Zn center (Zn···C3 = 2.20 Å; Zn···O = 2.25 Å), and the increasing degree of Zn···C3 interaction from **F1** to **G1** is also reflected in enhanced arenium cation character within the five-membered ring, with increased pyramidalization at C3, a shortening N1–C2 bond and lengthening of the C2–C3 and C3–C4 bonds. Due to steric constraints, the intact indole-borane product in **G1** lies approximately perpendicular to the (NacNac)Zn plane. Product release proceeds via associative substitution with CatBH, although this requires a low energy rearrangement from the κ-C binding mode in **G1** to the κ-O isomer (**G2**) in order to provide sufficient space for CatBH binding to form **H**.

The calculations indicate that *N*-methylindole C–H borylation with CatBH is thermodynamically favored by 6.6 kcal/mol and proceeds with an overall barrier of 22.4 kcal/mol relative to **1**, consistent with the room temperature reactivity seen experimentally with *N*-methylindole. C–B coupling is best described as a classical stepwise S_E_Ar process via arenium-type intermediates, **E1**/**E2**, mediated by the [(NacNac) Zn(CatBH)]^+^/ DMT frustrated Lewis pair. Dehydrocoupling via **TS(F2-G1)** corresponds to the rate-limiting transition state, although **TS(E1-E2)** (B–C3 rotation) and **TS(E2-F1)** (C–H deprotonation) are very close in energy.

An alternative mechanism involving a C–H activation/B–H activation sequence was also assessed computationally. This showed the initial formation of a C3-metalated [(NacNac)Zn(*N*-Me-indole)] complex and free [DMTH]^+^ to be kinetically accessible, but endergonic (Δ*G*^‡^ = +14.0 kcal/mol; Δ*G* = +8.8 kcal/mol). However, the subsequent B–H activation entailed a transition state at +26.6 kcal/mol, and so this pathway was not competitive with the process shown in Figure 4 (see Figure S49 for more details).

**Scheme 3:**
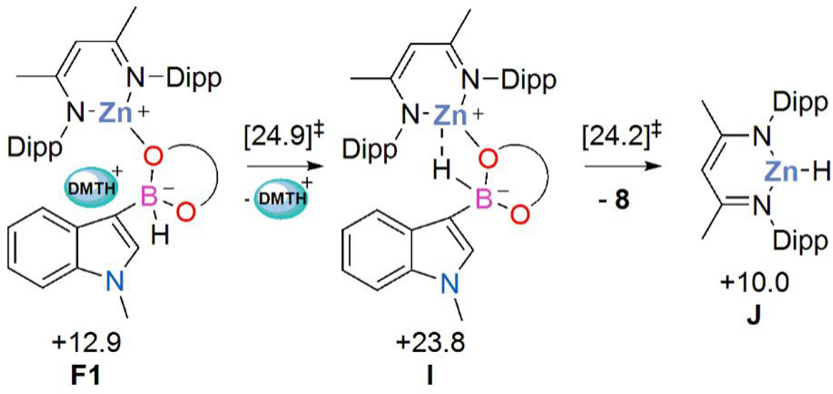
Side Reaction of **F1** to Form (NacNac)ZnH, **J**, with Computed Free Energies in kcal/mol

One interesting side reaction emerging from the computational study was characterized when modeling the loss of the [DMTH]^+^ cation from intermediate **F1** (see Scheme 3). This resulted in the formation of a B–H → Zn σ-interaction in intermediate **I** (comparing **F1** to **I** reveals the B–H distance has elongated from 1.21 to 1.35 Å concomitant with Zn–O bond elongation) from which hydride transfer readily occurs with the release of product **8** and the formation of (NacNac)ZnH, **J**. This pathway has it highest lying transition state at +24.9 kcal/mol, only 2.5 kcal/mol higher than the main C–H borylation profile. Kinetically this process is therefore in potential competition with the C–H borylation. A kinetically facile onward reaction of (NacNac)ZnH could account for the formation of Zn metal noted in the experimental studies run under prolonged heating, and this is investigated further below.

The dehydrocoupling reaction of (NacNac)ZnH with [DMTH]^+^ was also modeled. In the context of the catalytic system this involved a transition state at +27.0 kcal/mol, too high to be competitive with the borylation pathway proceeding from **F1** to **G**. However, when considered as an isolated step this models the experimental formation of **1** via Route 2 and this is computed to have a readily accessible barrier of 17.0 kcal/mol and to be exergonic by 16.6 kcal/mol.

### Additional Experiments Based on DFT Observations

The mechanism elucidated from the DFT studies highlighted two points warranting further investigation: (i) the potential consequence of forming (NacNac)ZnH *in situ*; (ii) the effect of varying the base as it is involved at multiple points in the catalytic cycle, including in the highest energy transition state. Regarding point (i), the formation of zinc metal from (NacNac)ZnH on heating (at 150 °C in the solid-state) has been previously reported,^27^ suggesting that (NacNac)ZnH may be involved in the decomposition process to form zinc metal observed during catalysis. While heating (NacNac)ZnH in chlorobenzene at 80 °C for 18 h led to no solid formation or reaction (by NMR spectroscopy), the addition of excess CatBH to (NacNac)ZnH led to a rapid reaction with heating at 80 °C leading to the decomposition of (NacNac)ZnH and the formation of a gray solid within 10 min. Analysis of the soluble materials from the decomposition of (NacNac)ZnH/CatBH revealed a mixture of several species (by ^1^H NMR spectroscopy) that could not be identified. Nevertheless, with the computed barrier to form (NacNac)ZnH only 2.5 kcal/mol higher than the rate limiting dehydrocoupling (for *N*-methylindole) step in the borylation process, this indicates that a sufficient concentration of (NacNac)ZnH could be present in solution to account for the catalyst decomposition by reaction with CatBH.

Regarding point (ii), a deeper analysis into the effects from varying the base on catalytic C–H borylation was performed. To assess this route 1 (Scheme 2) was used to generate analogues of **1** containing different bases, with the bases initially restricted to compounds similar to DMT to minimize changes due to a variation in steric environment around the donor atom (see Table 1). Note that the p*K*_a_ values used are the predicted values in MeCN using a recent highly accurate model.^28^

**Table 1:**
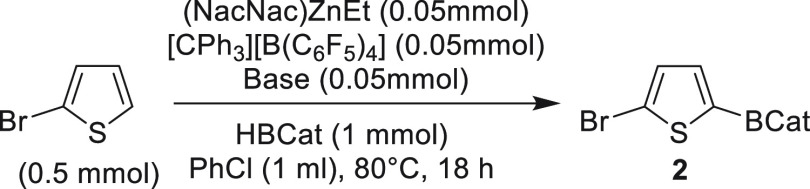

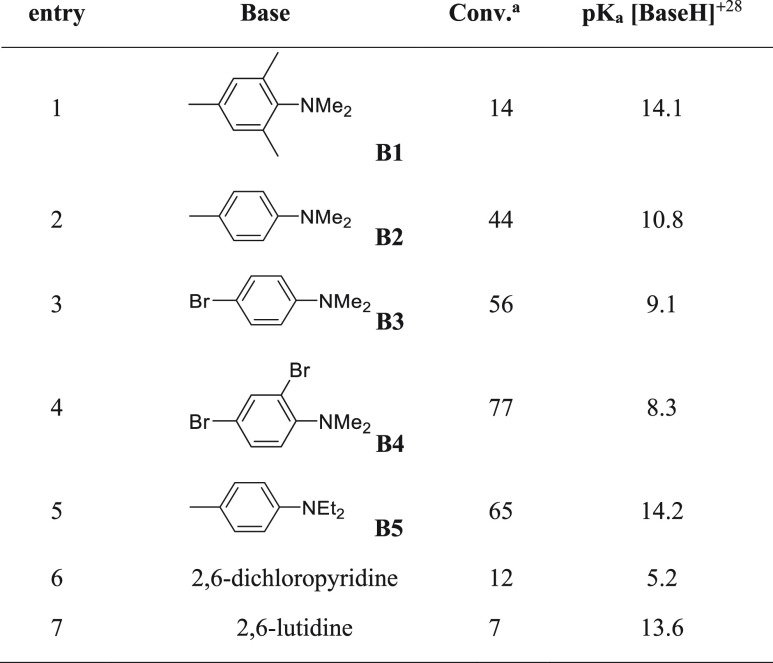
Effect of Base Variation on C–H Borylation^a^

a Conversions vs. CH_2_Br_2_ added as an internal standard after 18 h.

In the borylation of 2-bromothiophene to form **2** a decrease in basicity increased the borylation conversion (entries 1–4), with the highest conversion to **2** observed for dibrominated base **B4** (entry 4). This could be attributed to one (or a combination) of (i) a less endergonic displacement of the weaker base **B4** from {(NacNac)Zn}^+^ by CatBH; (ii) the more acidic conjugate acid ([H-**B4**]^+^) resulting in a lower barrier dehydrocoupling step (the reaction of zinc-bound borohydride with the ammonium salt, analogous to **TS(F2-G1)** in the computational study). Support for the former is forthcoming from the use of *N,N*-Et_2_-toluidine (**B5**, entry 5), which has more steric bulk around N but is more basic than DMT (**B2**), increasing the conversion to **2** relative to using DMT (entry 2) and using a dimethyl-aniline derivative of comparable basicity, **B1** (entry 1). The greater steric bulk in **B5** is potentially weakening the Zn–N dative bond, which may facilitate its displacement by CatBH. However, it should be noted that altering the steric environment around the basic site also can lead to dramatic retardation of borylation. For example, 2,6-disubstituted pyridines (often used as bases in electrophilic borylation reactions)^29^ give extremely poor outcomes regardless of p*K*_a_ (entries 6 and 7). As amine displacement by CatBH will be an associative process (given the high energy of “free” [(NacNac)Zn]^+^”) the 2,6-disubstituted pyridines may shield the zinc center too effectively from incoming CatBH. Regardless, these results show the sensitivity of borylation to both the p*K*_a_ and the steric bulk of the base. Finally, the use of the optimal base from Table 1 does indeed provide slightly enhanced reactivity in borylation of the most challenging substrate in this study: using **B4** in route 1 in place of DMT led to the borylation of benzothiophene in 26% conversion after 18 h at 80 °C, in contrast to only 14% conversion using DMT after 18 h at 80 °C. The modest increase in reactivity observed on switching DMT for **B4** indicates that while altering the base can have a positive effect, altering the electrophilic organometallic complex has a more dramatic effect on controlling the C–H borylation reactivity (compare outcomes using zinc cation **C** versus **D**) and that this is where future endeavors to expand the scope of the catalytic borylation approach should focus.

## Conclusions

This work demonstrates that (i) the activation of dioxaborolanes by electrophilic metal complexes is an approach applicable to distinct electrophilic zinc complexes, specifically [(NHC)ZnR]^+^ and [(NacNac)Zn]^+^ derivatives, and (ii) the borylation substrate scope is significantly affected by changing the zinc complex. A highly electrophilic [(NacNac)Zn]^+^ derivative is able to bind catecholborane (CatBH) and activate it to a greater extent (based on the relative CatB-based LUMO energies) than when using [(NHC)ZnR]^+^ electrophiles. This leads to an expanded substrate scope for catalytic C–H borylation, with this FLP-catalyzed borylation being the first such process able to borylate weakly activated heteroarenes such as 2-bromothiophene and benzothiophene.^30^ The robust and well-defined nature of the [(NacNac)Zn(DMT)]^+^ precatalyst enabled detailed computational analysis which indicated that an endergonic, but kinetically accessible, displacement of DMT by CatBH at zinc is essential to get on cycle. This formed an adduct containing a short Zn···O_(CatBH)_ interaction and a strongly activated CatBH moiety that can be viewed as a borenium cation equivalent. The borenium/DMT FLP then is calculated to mediate C–H borylation by stepwise C–B bond formation, arenium deprotonation, B–H/[DMT-H]^+^ dehydrocoupling, and finally ArylBCat displacement by CatBH. These computational studies indicated that both the metal complex and the base are crucial for effective C–H borylation. Indeed, variation in the base revealed a sensitivity to both steric and electronic factors, and for *N,N*-dialkyl anilines less nucleophilic bases gave superior C–H borylation results, an observation we attribute to the displacement of the base from [(NacNac)Zn]^+^ by CatBH being less endergonic with less nucleophilic amines. Finally, it is clear that the properties of the electrophilic metal activator are crucial, thus electrophilic metal complexes based on elements other than zinc will alter the nature of the metal···O(dioxaborolane) interaction and should enable access to more reactive borenium equivalents.

## Supporting Information Available

NMR spectra for all new compounds, *in situ* NMR spectra for catalytic and mechanistic reactions (PDF)
Cartesian Coordinates for all calculated structures (XYZ)

## Supplementary Material

cs2c05995_si_001.pdf

cs2c05995_si_002.xyz
