# Supplementary material for: Understanding and Expanding Zinc Cation/Amine Frustrated Lewis Pair Catalyzed C–H Borylation
Source: ACS Catal. 2023 Jan 30;13(4):2286–94. doi: 10.1021/acscatal.2c05995 (PMC9942201; doi:10.1021/acscatal.2c05995)
Supplement: Supplementary file 1 — cs2c05995_si_001.pdf [file cs2c05995_si_001.pdf]

Understanding and Expanding Zinc Cation / Amine Frustrated Lewis Pair Catalyzed C-H Borylation.

Matthew E. Grundy[a†], Lia Sotorrios[a‡], Milan Kumar Bisai[†], Kang Yuan[†], Stuart A. Macgregor[‡], Michael J. Ingleson[†]**

† EaStCHEM School of Chemistry, University of Edinburgh, Edinburgh, EH9 3FJ, UK.

‡ Institute of Chemical Sciences, Heriot-Watt University, Edinburgh, EH14 4AS

mingleso@ed.ac.uk

Contents

S1. General information 2

S2. Initial computational data at level used in previous reports 3

S3. Synthesis of unreported compounds or novel syntheses 7

S4. C-H Borylation 13

S5. Variation of Base 35

S6. Stoichiometric reactions with NacNacZnEt 37

S7. Generation of catalyst **1** from NacNacZnH Vs from NacNacZnEt 41

S8. Thermal stability tests 43

S9. Control reactions 46

S10. Computational details: Mechanistic Studies 48

S11. References 83

S1. General information

Unless otherwise stated, all the experiments were carried out under an inert atmosphere using either standard Schlenk techniques or in a MBraun glovebox (< 0.1 ppm H_2O / O_2). Chlorobenzene, d_6-benzene and d_5-bromobenzene were distilled over CaH_2 and stored over 3 Å molecular sieves. All other solvents were obtained from an Inert PureSolv MD5 SPS. Unless otherwise stated all chemicals were purchased from commercial sources and used as received. Trityl tetrakis(pentafluorophenyl)borate,[1] NacNacH (NacNac = {2,6-*i*$Pr_2C_6H_3NC(Me)\}_2CH$),[2] NacNacZnEt[3] and NacNacZnH[4] were prepared according to reported literature procedures.

^{1}H, $^{13}C\{^{1}H\}$, ^{11}B, $^{11}B\{^{1}H\}$, and ^{19}F NMR spectra were recorded on Bruker Advance III 400, Bruker Advance III 500MHz or Bruker PRO 500 MHz spectrometers. Chemical shifts are reported as dimensionless δ values and are frequency referenced relative to residual protio-solvent signals in the NMR solvents for ^{1}H and $^{13}C\{^{1}H\}$, while ^{11}B and ^{19}F shifts are referenced relative to external $BF_3.Et_2O$ and hexafluorobenzene, respectively. Coupling constants J are given in Hertz (Hz) as positive values regardless of their real individual signs. Unless otherwise stated NMR spectroscopy was undertaken at room temperature (~20°C). The multiplicity of the signals are indicated as "s", "d", "t" "q" "pent", "sept" or "m" for singlet, doublet, triplet, quartet, pentet, septet or multiplet, respectively. Mass spectrometry was performed by the University of Edinburgh, School of Chemistry, Mass spectrometry Laboratory using electrospray ionisation.

It should be noted that the very broad signals observed at ca. 0 ppm in the ^{11}B NMR spectra are due to the use of borosilicate glass NMR tubes and boron containing parts in the NMR cavity. These are not coincident with the broad compound signals.

S2. Initial computational data at level used in previous reports

The calculation was performed using the Gaussian09 series of programs.[5] Geometry optimisations were completed with the B3PW91[6] functional. Calculations were run with a 6-311G(d,p) (for H, B, C, N, O, F) basis set and a LANL2DZ (for Zn) basis set. The optimization was full, with no restrictions. Stationary points located in the potential energy surface were characterized as minima (no imaginary frequencies) by vibrational analysis. Solvent effects of the chlorobenzene were introduced using the self-consistent field approach, by means of the integral equation formalism polarizable continuum model (IEFPCM).[7]

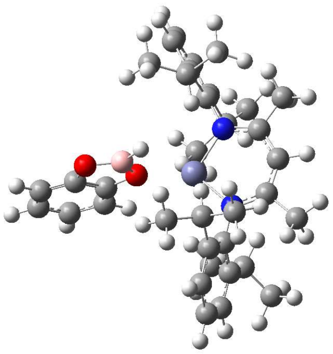

NacNacZn-HBCat

E(RB3PW91) = -1711.603552

Zn	10.12251600	1.03967700	3.23946200
N	8.84019800	2.10328000	4.35161500
N	10.50787200	2.28771800	1.72117300
C	7.67367500	4.21843100	4.79312200
C	8.55160100	3.33958400	3.93690500
C	9.03169800	3.93066500	2.75259300
C	9.92276400	3.48863600	1.75633000
C	10.24522200	4.49383100	0.67798700
C	8.34056300	1.61978200	5.60684400
C	9.00069200	1.97409000	6.80327200
C	8.50790200	1.47178100	8.00803600
C	7.39415600	0.64482500	8.04149300
C	6.76267700	0.29378100	6.85772300
C	7.22078300	0.76177400	5.62399500
C	10.26041400	2.82498300	6.79900900

C	10.30672300	3.86094100	7.92608600
C	11.49724100	1.91835500	6.84208700
C	6.49428000	0.36444600	4.34936200
C	5.27533900	1.26290800	4.09457400
C	6.07808400	-1.11008800	4.33353200
C	11.40923500	1.95251400	0.65899400
C	10.93937200	1.17562400	-0.42067600
C	11.83536800	0.84217600	-1.43853200
C	13.16168300	1.24517800	-1.38856800
C	13.61626600	1.98702300	-0.30704700
C	12.75926400	2.35504900	0.73082100
C	9.49194400	0.71942100	-0.49862100
C	9.34534400	-0.71995400	-1.00186200
C	8.64186200	1.66821100	-1.35544600
C	13.29923700	3.11651100	1.93100200
C	14.31836700	4.19866900	1.56260200
C	13.89361500	2.13402000	2.94893600
H	7.18810300	0.52316400	3.51831500
H	5.89498600	-0.35571200	6.89259700
H	12.45735700	3.61661800	2.41733600
H	14.65774000	2.28692500	-0.27038100
H	10.29344900	3.37301800	5.85406100
H	13.84291800	0.97703000	-2.18932000
H	8.66503000	4.93547300	2.59036400
H	11.48970500	0.25623600	-2.28312600
H	9.00271200	1.73311200	8.93707700
H	9.09390400	0.76305200	0.52092000
H	14.57215100	4.78866600	2.44785200
H	10.35800300	4.02300800	-0.29883500
H	7.02113300	0.27140900	8.98945900
H	11.17626600	4.51199900	7.79816700
H	13.14621600	1.39933100	3.26603800
H	11.19521900	4.98873500	0.90480500

H	9.47384300	5.26094900	0.62159100
H	8.27745000	4.70014200	5.56925600
H	12.41873400	2.50382300	6.77189700
H	4.76911200	0.96884700	3.17006200
H	7.21477800	5.00416800	4.19389300
H	13.92427700	4.87981900	0.80354900
H	14.73409600	1.58355300	2.51588200
H	9.01875400	1.70167600	-2.38239300
H	6.89511400	3.64892400	5.30059500
H	11.48104200	1.20385800	6.01310600
H	14.25165400	2.65980700	3.83902000
H	6.91408000	-1.77402000	4.57260800
H	10.39597700	3.39217600	8.91019000
H	4.55567700	1.17909200	4.91492400
H	15.25024300	3.77225500	1.18048800
H	9.41190500	4.48906500	7.93288000
H	9.99201000	-1.41287400	-0.45580600
H	8.64587000	2.68667200	-0.96186300
H	5.69910000	-1.38183900	3.34424600
H	5.27816600	-1.31470000	5.05063300
H	7.60295400	1.32603500	-1.38856800
H	9.59301400	-0.80586000	-2.06368900
H	8.31054400	-1.05461600	-0.88627900
H	11.52853500	1.34169700	7.77131700
H	5.55795000	2.31372300	4.00176000
C	11.17502900	-2.02201600	3.69071200
C	10.74049600	-3.28987000	3.34012200
C	12.32447000	-1.80163100	4.41618400
C	11.44175100	-4.42273800	3.69591700
C	13.04349200	-2.94290800	4.78176400
H	12.66461800	-0.81232400	4.69447700
C	12.61228200	-4.22172800	4.42949200
H	11.09528100	-5.41011000	3.41769900

H	13.95647300	-2.82508700	5.35316100
H	13.19821600	-5.08155800	4.73239000
O	10.24691400	-1.10021300	3.18050800
B	9.26432700	-1.87996600	2.53028200
H	8.33077600	-1.40639400	1.99386300
O	9.56347900	-3.20292900	2.62540100

S3. Synthesis of unreported compounds or novel syntheses

[DMTH][B(C_6F_5)$_4$]

A solution of *N,N*-dimethyl-4-toluidine (85 µL, 0.59 mmol) in dry diethyl ether (10 mL) was made in a dry Schlenk flask. HCl (2M in Et_2O, 1.5 mL, 1.85 mmol) was added to the stirring solution of *N,N*-dimethyl-4-toluidine and allowed to react for 10 minutes yielding a white suspension. The volatiles were removed *in vacuo* leaving a white powder and the flask transferred to a glovebox. Potassium tetrakis(pentafluorophenyl)borate was added to the flask. The flask was then removed from the glovebox and the powders were suspended in dry DCM (10 mL) under an inert atmosphere and allowed to stir for 72 hours. The mixture was then filtered to remove KCl and the volatiles removed *in vacuo* yielding an off white powder. *Yield*: 87% (420 mg)

^{1}H NMR (500 MHz, CD_2Cl_2): δ 7.47 (d, J = 8.0 Hz, 2H, *Ar*), 7.30 (d, J = 8.8 Hz, 2H, *Ar*), 7.20 (br. s, NH), 3.45 (s, 6H, NMe_2), 2.48 (s, 3H, Me).

^{11}B NMR (160 MHz, CD_2Cl_2): δ – 16.6 (s).

^{13}C{^{1}H} NMR (126 MHz, CD_2Cl_2): δ 148.1 [d, $^1J_{C\text{-}F}$ = 240.8 Hz, B(C_6F_5)$_4$], 143.4 (s, DMT-*C*), 138.3 [d, $^1J_{C\text{-}F}$ = 240.7 Hz, B(C_6F_5)$_4$], 137.8 (s, DMT-*C*), 136.3 [d, $^1J_{C\text{-}F}$ = 241.3 Hz, B(C_6F_5)$_4$], 132.0 (s, DMT-*C*), 118.8 (s, DMT-*C*), 48.8 (N*Me*$_2$), 20.8 (*Me*).

^{19}F NMR (471 MHz, CD_2Cl_2): δ -133.25 (m), -163.19 (t, J = 21.35 Hz), -167.28 (t, J = 22.9 Hz).

Mass Spectrometry: Calculated [M^+] = 136.1121, Observed [M^+] = 136.1141; Calculated [M^-] = 678.9774, Observed [M^-] = 678.9957.

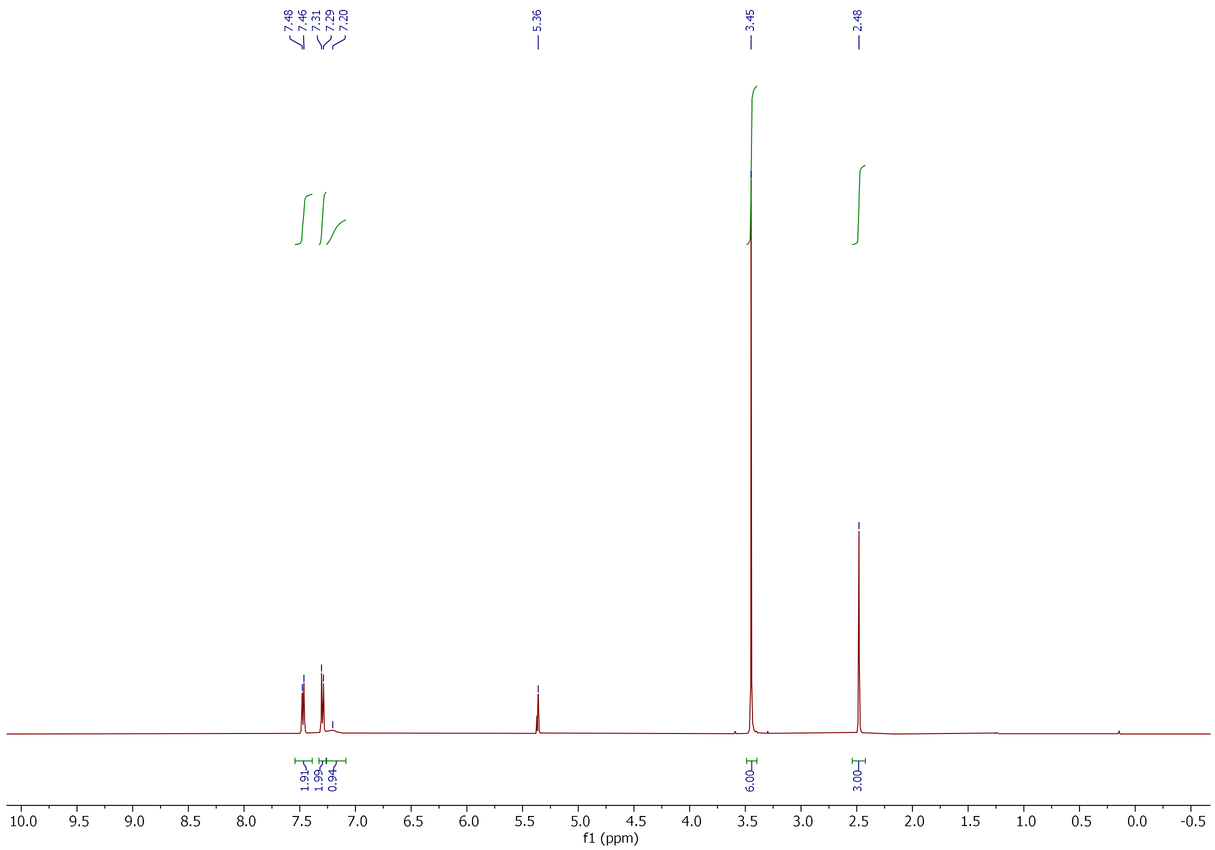

Figure S1: ^{1}H NMR spectroscopy of [DMTH][$B(C_6F_5)_4$] in CD_2Cl_2.

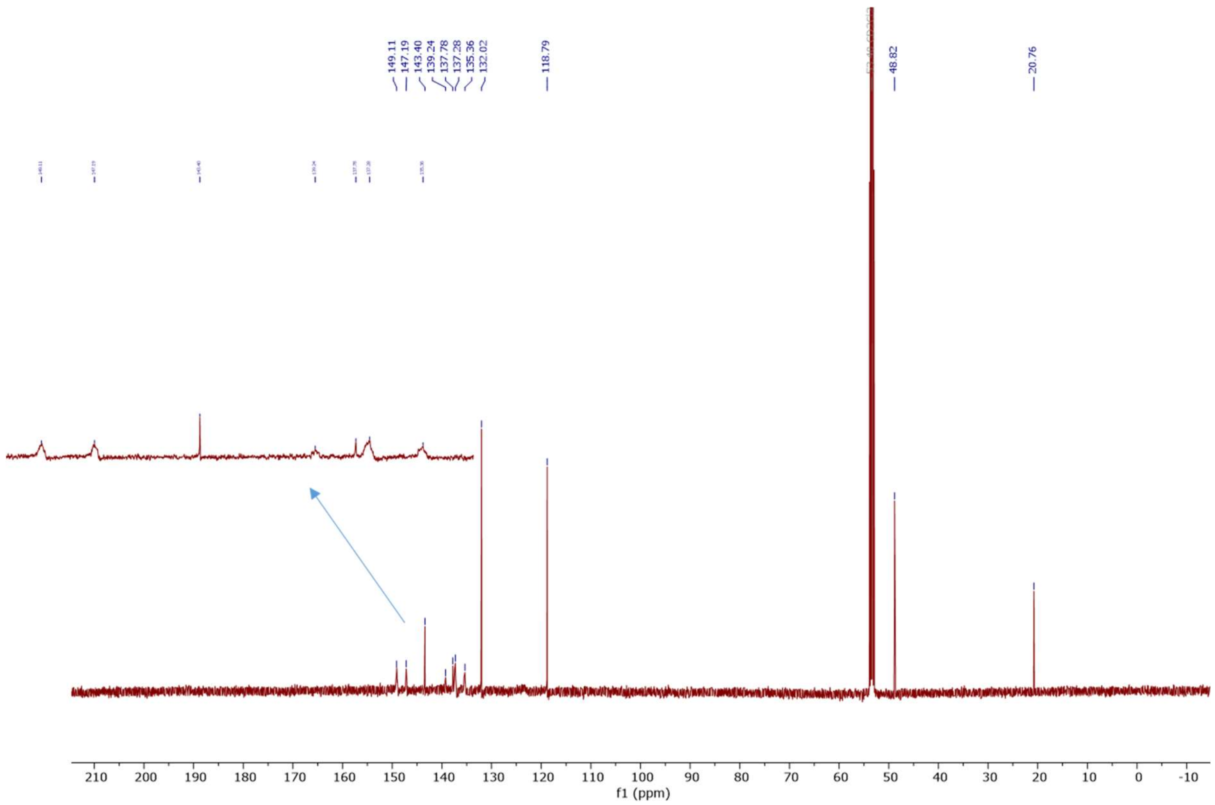

Figure S2: $^{13}C\{^{1}H\}$ NMR spectroscopy of [DMTH][$B(C_6F_5)_4$] in CD_2Cl_2.

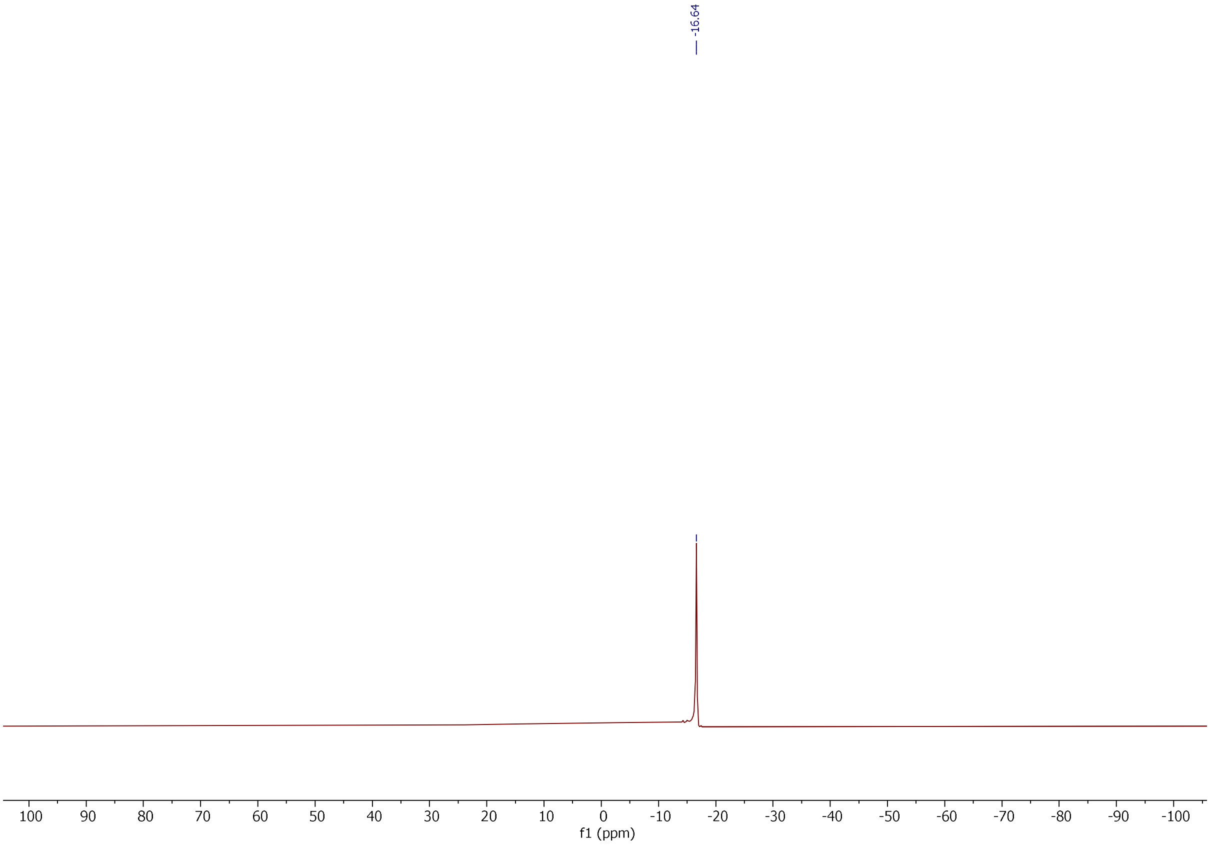

Figure S3: ^{11}B NMR spectroscopy of [DMTH][$B(C_6F_5)_4$] in CD_2Cl_2.

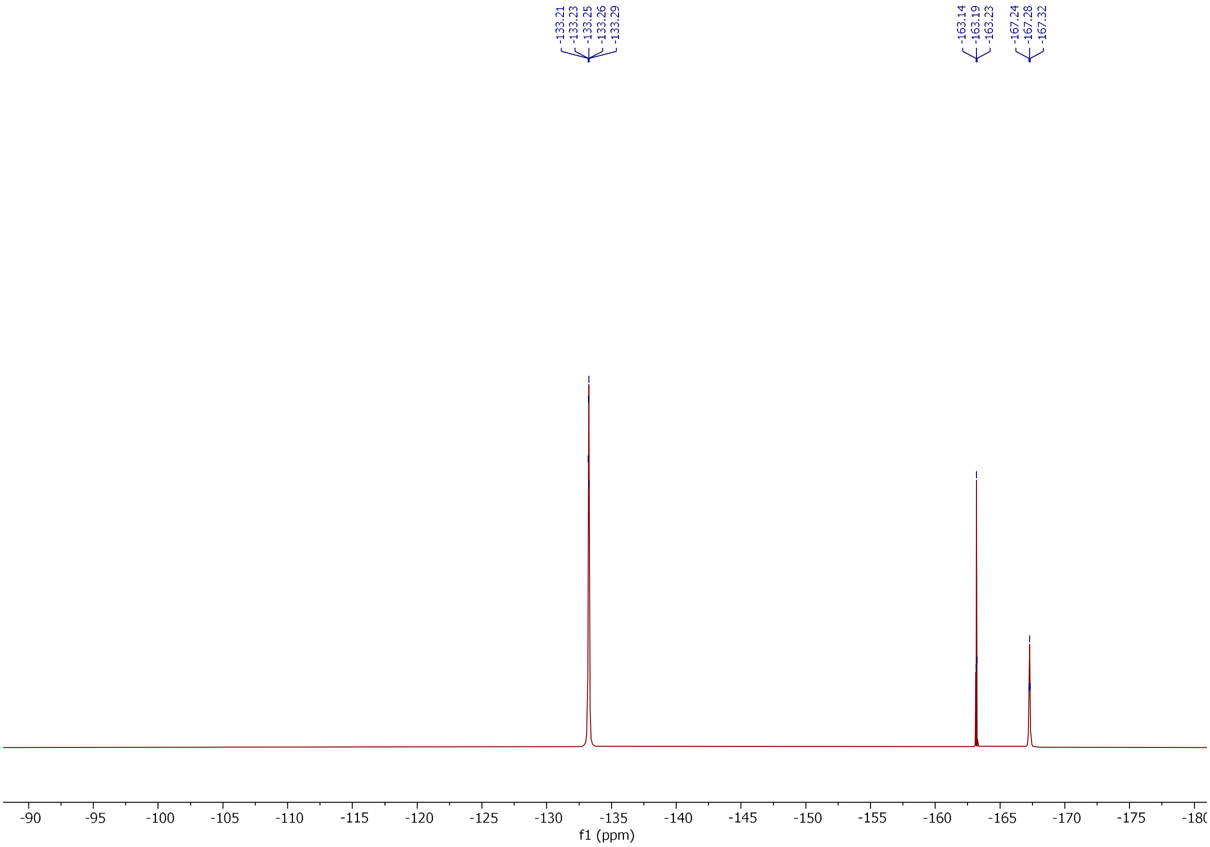

Figure S4: ^{19}F NMR spectroscopy of [DMTH][$B(C_6F_5)_4$] in CD_2Cl_2.

N,N-dimethyl-2,4-dibromoaniline

4-bromo-*N,N*-dimethylaniline (2.00 g, 10 mmol) and *N*-bromosuccinimide (1.78 g, 10 mmol) were added to a round bottomed flask and dissolved in DCM (ca. 10 mL). The reaction was then stirred at room temperature for 24 hours before removal of the volatiles *in vacuo* yielding a clear oil. *Yield*: 17% (462 mg). The spectral data are consistent with that reported previously.[8]

Compound 1

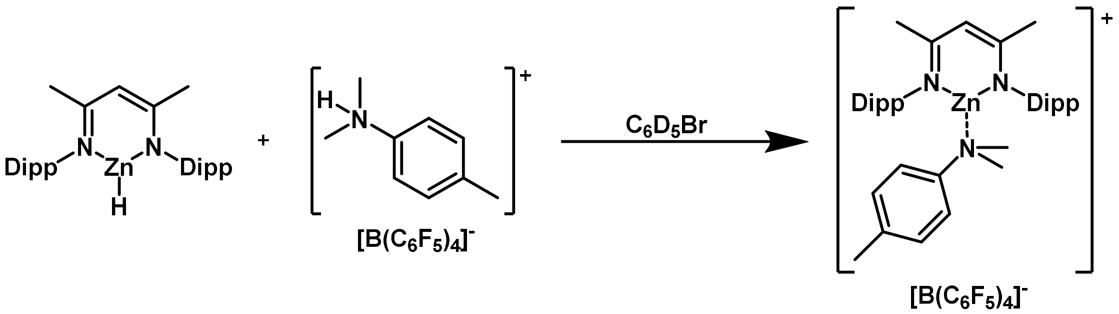

A J Young's NMR tube was charged with NacNacZnH (24 mg, 0.05 mmol) and [DMTH][B(C_6F_5)$_4$] (40.8 mg, 0.05 mmol) and dissolved in C_6D_5Br (0.5 mL) causing evolution of gas. Upon completion (as indicated by the absence of any gas evolution) the reaction mixture was monitored by ^1H, $^{13}C\{^1H\}$, ^{11}B and ^{19}F NMR spectroscopy.

^1H NMR (500 MHz, C_6D_5Br): δ 7.08 (t, J = 7.8 Hz, 2H), 6.96 (d, J = 7.7 Hz, 4H), 6.74 (d, J = 8.5 Hz, 2H), 6.43 (d, J = 8.7 Hz, 2H), 5.05 (s, 1H), 2.68 (Sept, J = 6.8 Hz, 4H), 2.06 (s, 6H), 2.00 (s, 3H), 1.61 (s, 6H), 0.98 (d, J = 6.8 Hz, 12H), 0.80 (d, J = 6.9 Hz, 12H).

^{11}B NMR (160 MHz, C_6D_5Br): δ -16.4 (s).

$^{13}C\{^1H\}$ NMR (126 MHz, C_6D_5Br): δ 173.1 (s, *C*CHC), 149.1 [d, $^1J_{C\text{-}F}$ = 241.3 Hz, B(C_6F_5)$_4$], 144.6 (s, Ar-*C*), 142.0 (s, Ar-*C*), 141.7 (s, DMT-*C*), 140.5 (s, Ar-*C*), 138.9 [d, $^1J_{C\text{-}F}$ = 241.3 Hz, B(C_6F_5)$_4$], 137.0 [d, $^1J_{C\text{-}F}$ = 237.4 Hz, B(C_6F_5)$_4$], 132.1 (s, Ar-*C*), 128.5 (s, Ar-*C*), 125.2 (s, DMT-*C*), 119.6 (s, DMT-*C*), 97.9 (s, C*C*HC), 50.3 (s, N*Me*$_2$), 29.3 (s, *C*HMe$_2$), 24.6 (s, CH*Me*$_2$), 24.2 (s, ^{Me}BDI), 24.0 (s, CH*Me*$_2$), 20.9 (s, ^{Me}DMT).

^{19}F NMR (471 MHz, C_6D_5Br): δ -131.89 (m), -162.45 (t, J = 23.0 Hz), -166.33 (t, J = 23.3 Hz).

Note multiple attempts to obtain solid material from these reactions failed, with either oils or clathrates formed. Furthermore, several attempts were made to perform mass spectrometry on this compound, but due to its sensitivity these all did not show the $[M]^+$.

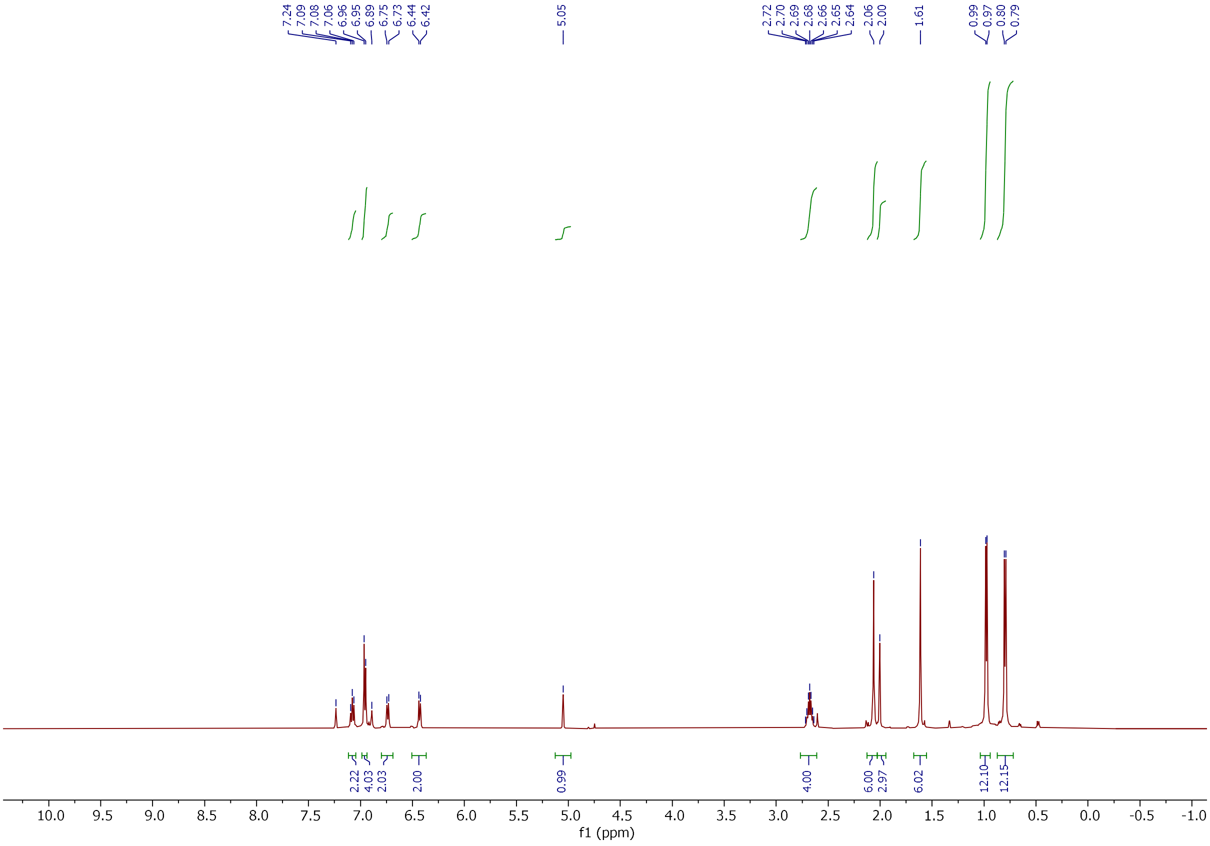

Figure S5: ^{1}H NMR spectroscopy of compound **1** in C_6D_5Br.

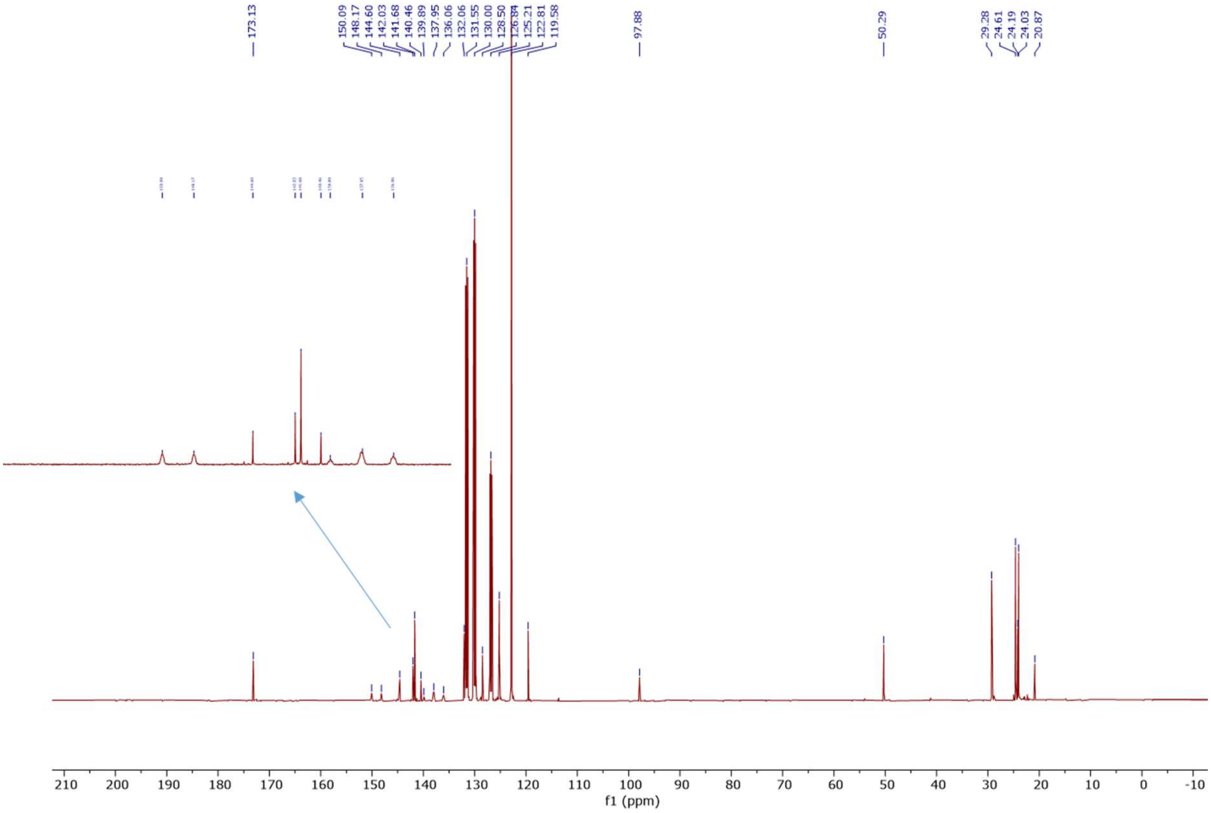

Figure S6: $^{13}C\{^{1}H\}$ NMR spectroscopy of compound **1** in C_6D_5Br.

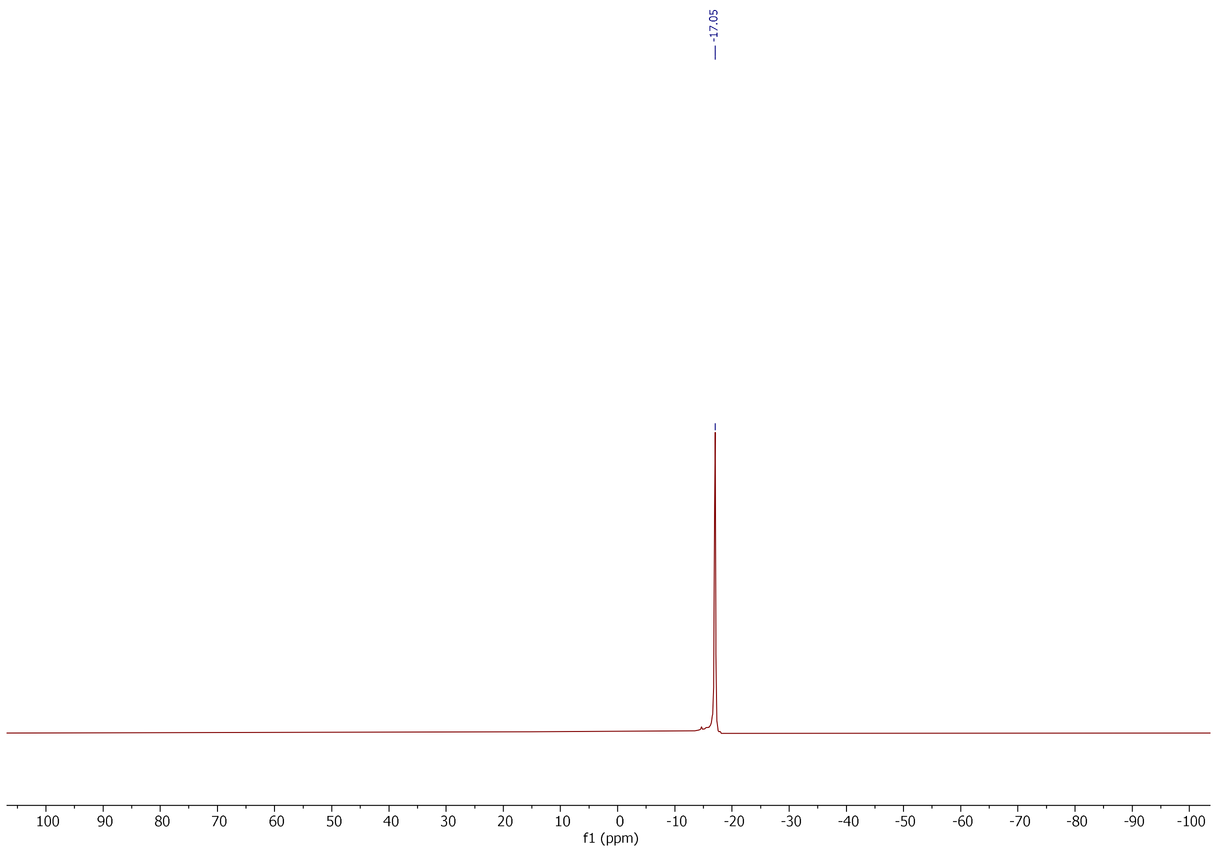

Figure S7: ^{11}B NMR spectroscopy of compound **1** in C_6D_5Br.

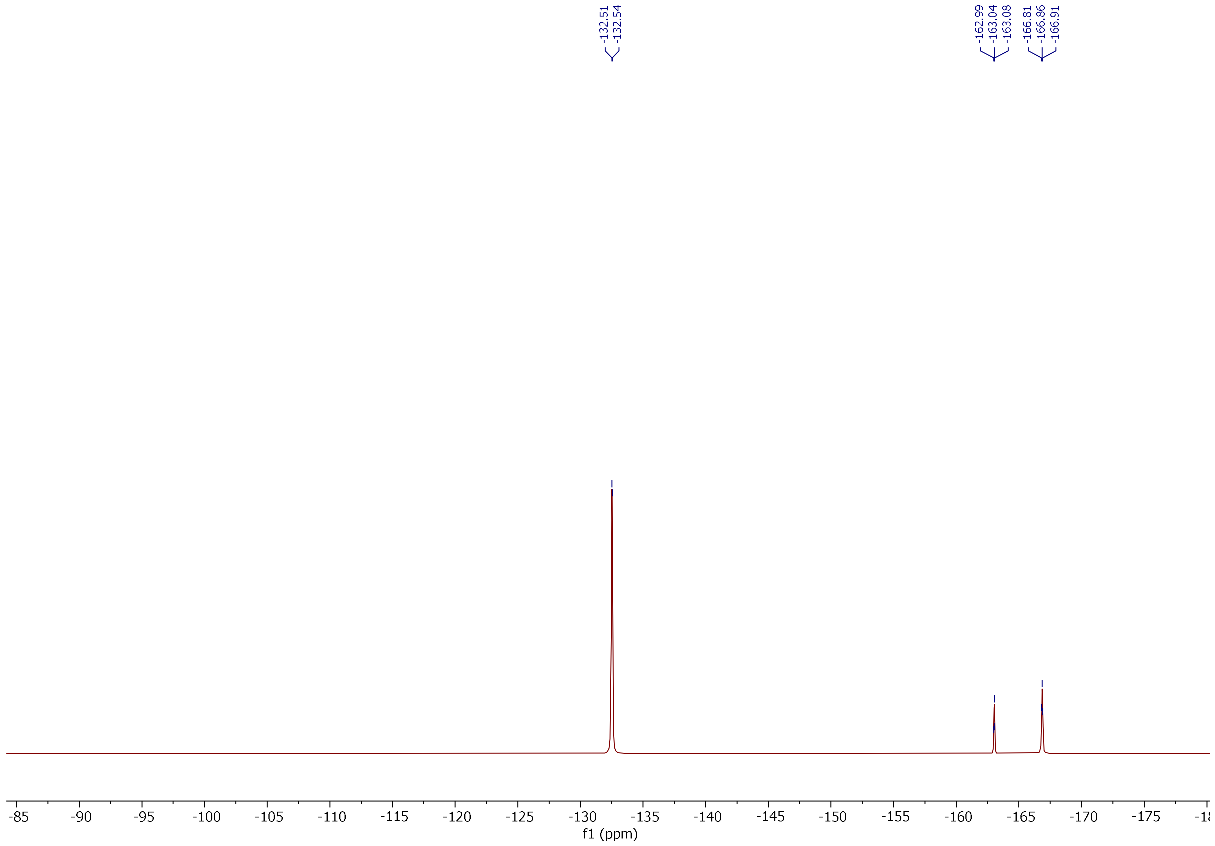

Figure S8: ^{19}F NMR spectroscopy of compound **1** in C_6D_5Br.

S4. C-H Borylation

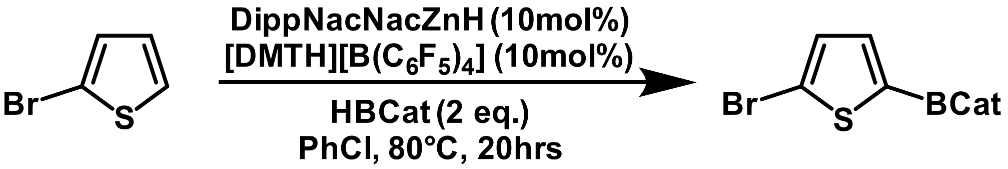

A J Young's NMR tube was charged with NacNacZnH (24 mg, 0.05 mmol) and [DMTH][B(C_6F_5)$_4$] (41 mg, 0.05 mmol) and dissolved in PhCl (1 mL), causing rapid evolution of gas, before addition of 2-bromothiophene (49 μL, 0.5 mmol) and catecholborane (107 μL, 1.00mmol). The reaction mixture was heated for 20 hours at 80°C and monitored by ^{1}H and ^{11}B NMR spectroscopy. Addition of dibromomethane (35 μL, 0.50 mmol) as an internal standard was done at the end of the reaction and used to determine *in-situ* yields by integration of diagnostic ^{1}H resonances.

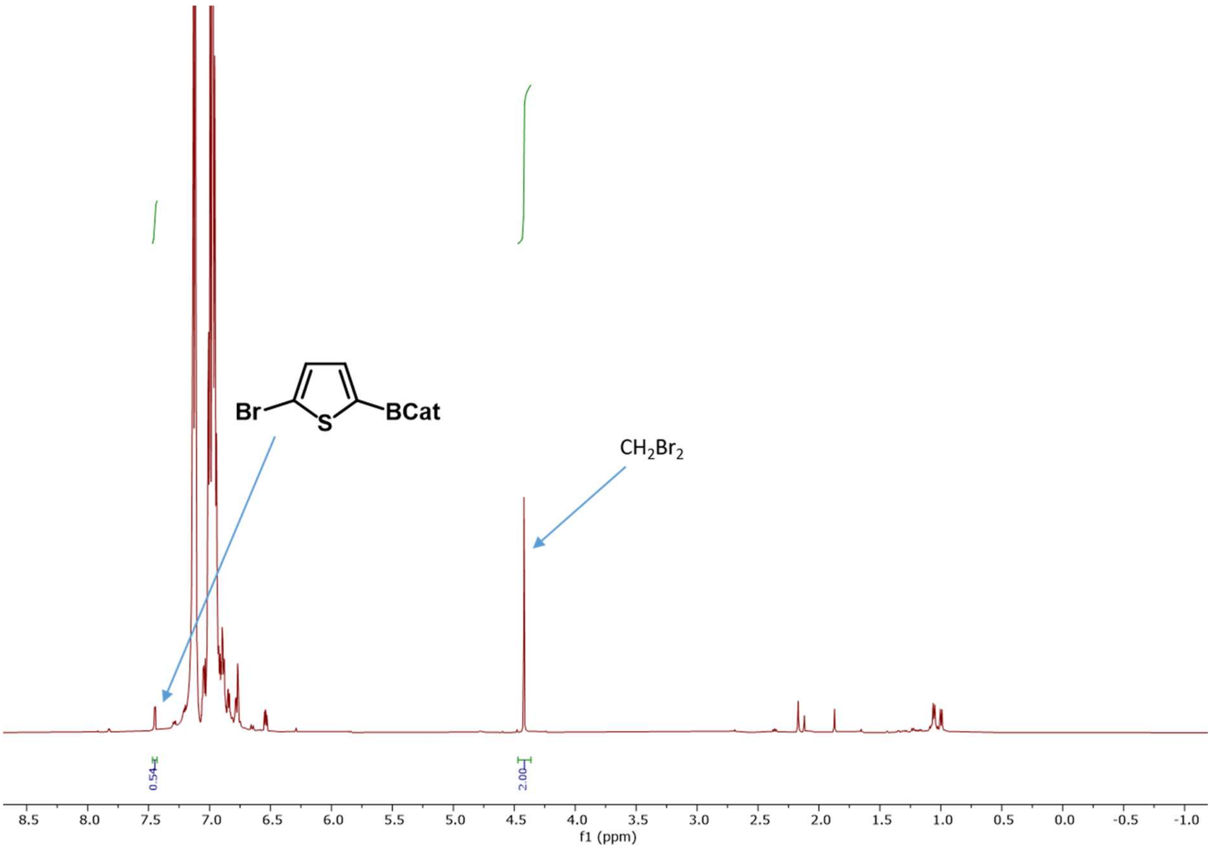

Figure S9: Borylation of 2-bromothiophene in PhCl with dibromomethane internal standard as observed by ^{1}H NMR spectroscopy.

General procedure A: A J Young's NMR tube was charged with NacNacZnEt (26 mg, 0.05 mmol) and $[CPh_3][B(C_6F_5)_4]$ (46 mg, 0.05 mmol) before dissolution in PhCl (1 mL) and immediate addition of *N*,*N*-dimethyl-4-toluidine (7.2 µL, 0.05 mmol), catecholborane (107 µL, 1.00mmol) and then substrate (0.50 mmol). The reaction mixture was heated for 18 hours at 80°C (unless otherwise specified) and monitored by ^{1}H and ^{11}B NMR spectroscopy. Addition of an internal standard, either dibromomethane (35 µL, 0.50 mmol) or mesitylene (70 µL, 0.50mmol), was done at the end of the reaction and used to determine *in-situ* yields by integration of diagnostic ^{1}H resonances.

Table S1 – Borylation scope using general procedure A.

Substrate	Conversion	Substrate	Conversion
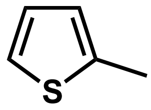	98%[a]	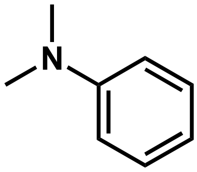	99%[a,b]
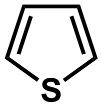	97%[a,c]	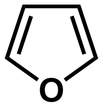	97%[a]
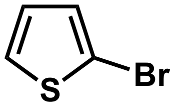	77%[a,e]	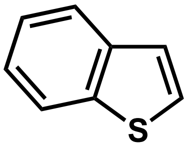	37%[a,d]
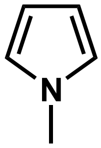	85%[a,f,g]	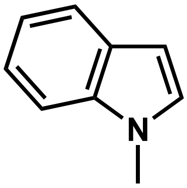	85%[a,f]

[a] Confirmed *in-situ* conversion by comparison to internal standard. [b] Heated for 1 hr. [c] 92:5 ratio mono- : di- BCat regioisomers. [d] Heated for 36 hrs at 100°C. [e] Heated for 60 hrs. [f] Heated for 10 mins. [g] 56:29 2- :3- BCat regioisomers.

Borylation of 2-methylthiophene

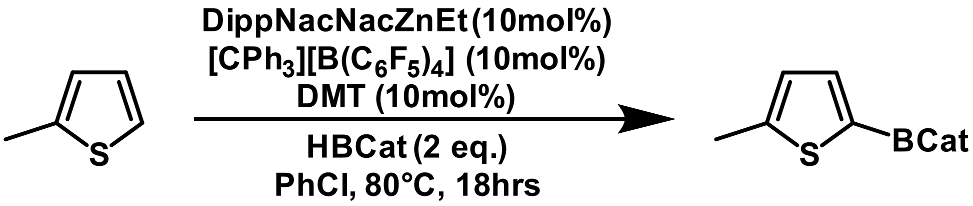

Yield: 98% by integration against internal standard.

Product was synthesised according to general procedure A and conversion versus an internal standard then determined (CH_2Br_2 35 µL, 0.5 mmol). Conversion to the boronic acid pinacol ester then was achieved by drying of the reaction mixture *in vacuo* before dissolving in DCM (0.5 mL) and reacting with a 1M solution of pinacol in NEt_3 (0.75 mL, 0.75 mmol) for 1 hr at room temperature. The reaction mixture was then extracted using (ca. 5 mL) a 9:1 mixture of pentane:ethyl acetate and filtered through a (ca. 5 cm) silica plug, further (ca. 5 mL) pentane:ethyl acetate solution was used to ensure the product eluted through the plug. The eluent was then dried *in vacuo* and redissolved in $CDCl_3$ allowing confirmation of the initial product as 2-methyl-5-(1,3,2-benzodioxaborole)-thiophene by comparison to the literature.[9]

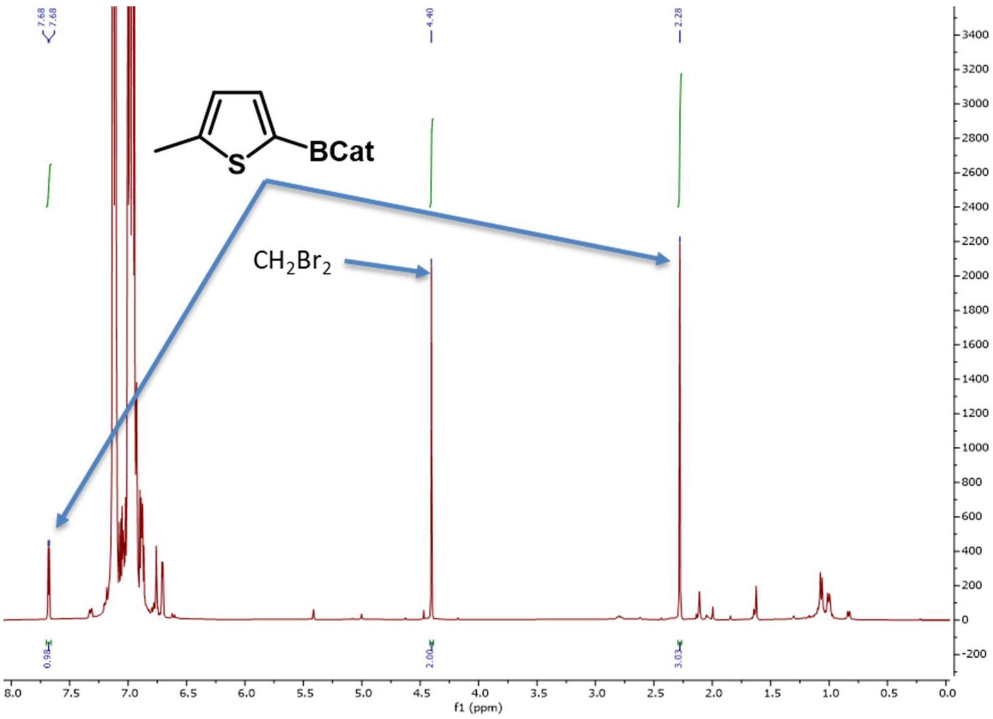

Figure S10: Borylation of 2-methylthiophene in PhCl with CH_2Br_2 internal standard as observed by in-situ ^{1}H NMR spectroscopy.

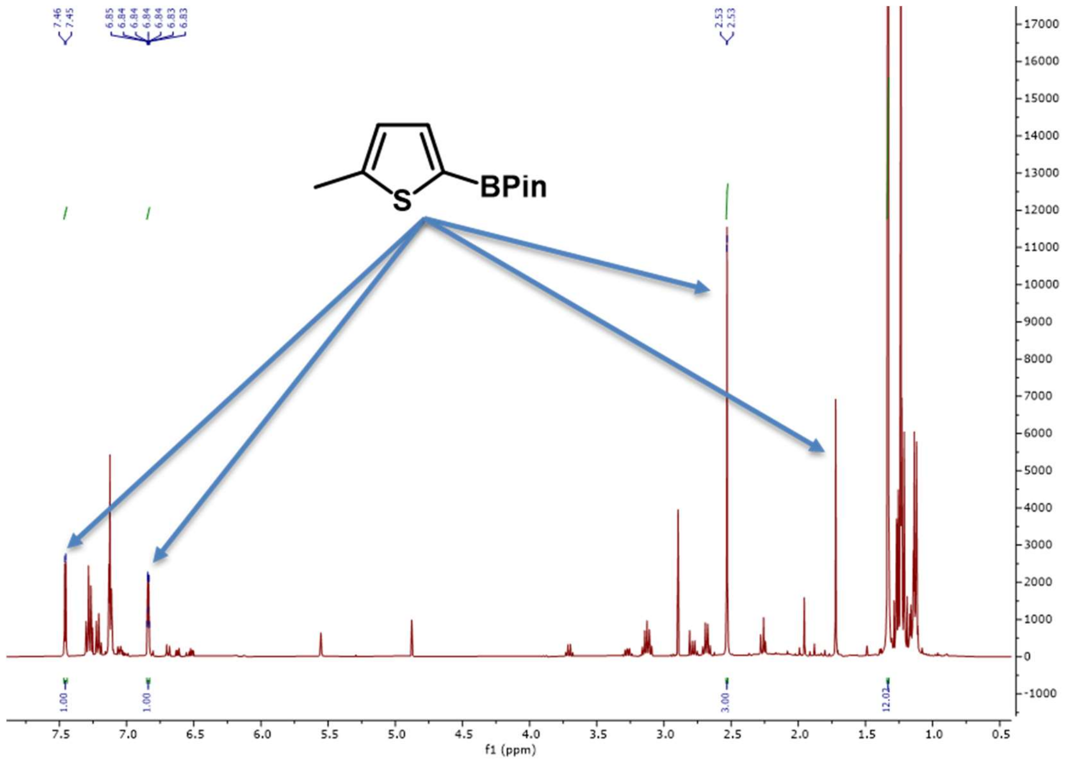

Figure S11: ^{1}H NMR Spectrum ($CDCl_3$) of crude BPin reaction mixture after filtration.

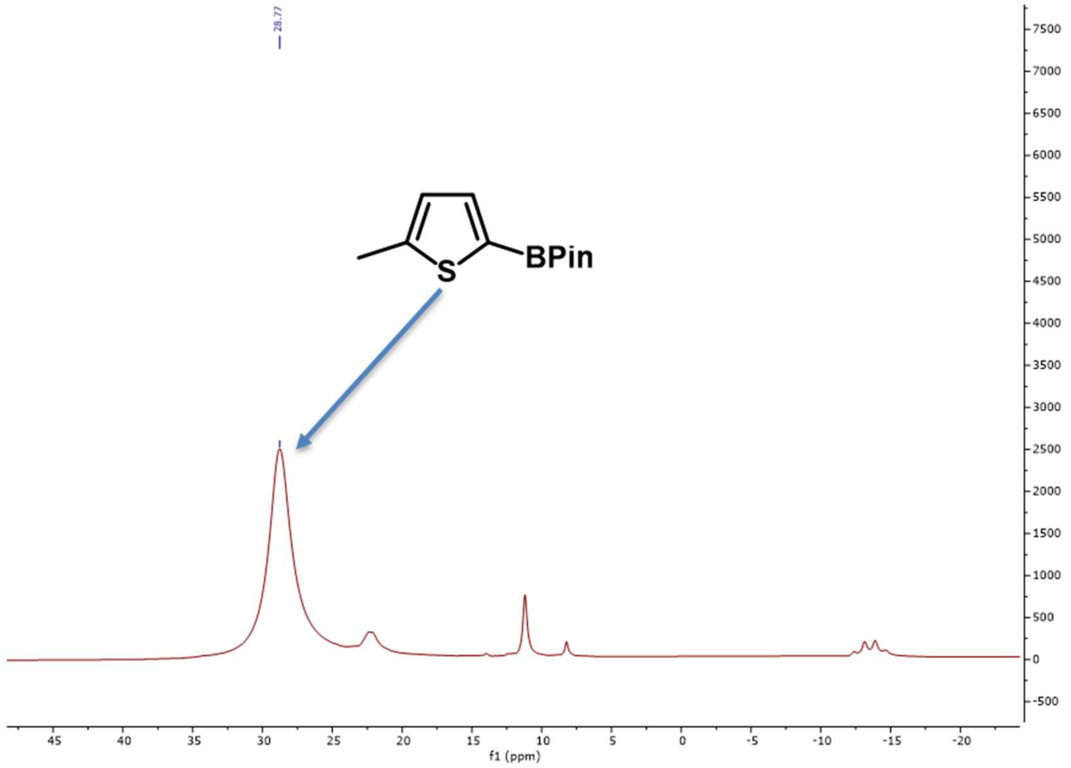

Figure S12: ^{11}B NMR Spectrum ($CDCl_3$) of crude BPin reaction mixture after filtration.

Borylation of thiophene

Yield: 97% (92:5 mono-:di- borylated products) by integration against internal standard.

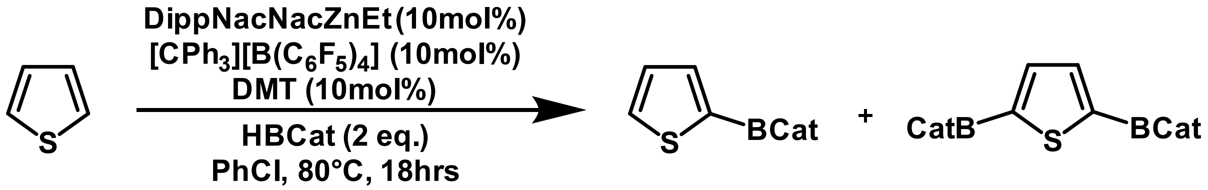

Product was synthesised according to general procedure A and conversion versus an internal standard then determined *in-situ* using mesitylene (70 µL, 0.5 mmol). Conversion to the boronic acid pinacol ester then was achieved by drying of the reaction mixture *in vacuo* before dissolving in DCM (0.5 mL) and reacting with 1M solution of pinacol in NEt_3 (0.75 mL, 0.75 mmol) for 18 hrs at room temperature. The reaction mixture was then extracted using (ca. 5mL) a 9:1 mixture of pentane:ethyl acetate and filtered through a (ca. 5cm) silica plug, further (ca. 5mL) pentane:ethyl acetate solution was used to ensure the product eluted from the plug. The eluent was then dried *in vacuo* and redissolved in $CDCl_3$ allowing confirmation of the initial product as mono borylated 2- isomer and diborylated 2,5-(1,3,2-benzodioxaborole)-thiophene by comparison to previously reported literature.[10]

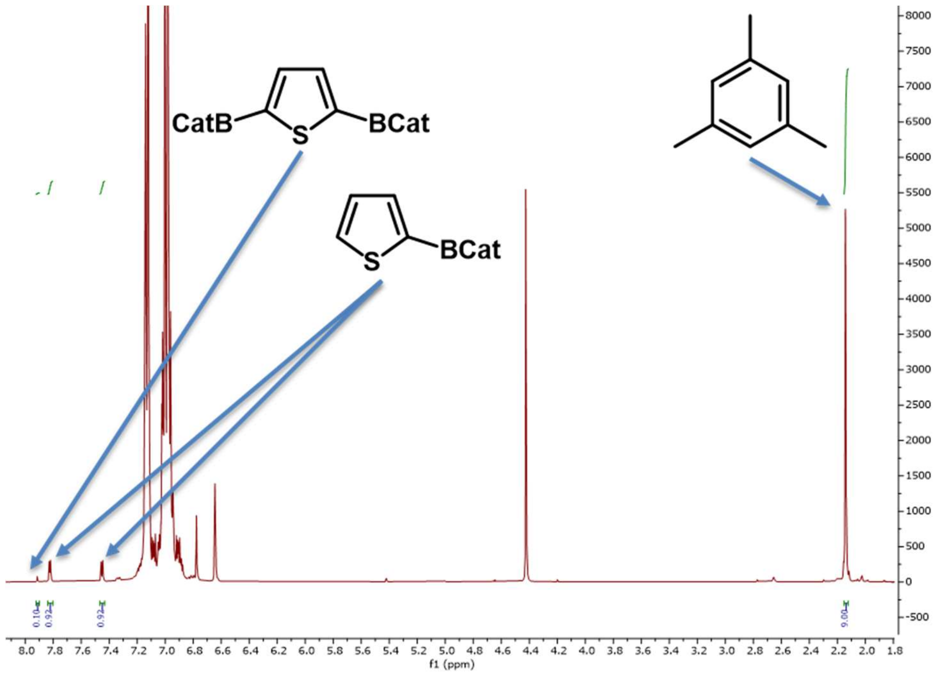

Figure S13: Borylation of thiophene in PhCl with mesitylene internal standard as observed by ^{1}H NMR spectroscopy. Note the resonance at 4.4 ppm is CH_2Br_2.

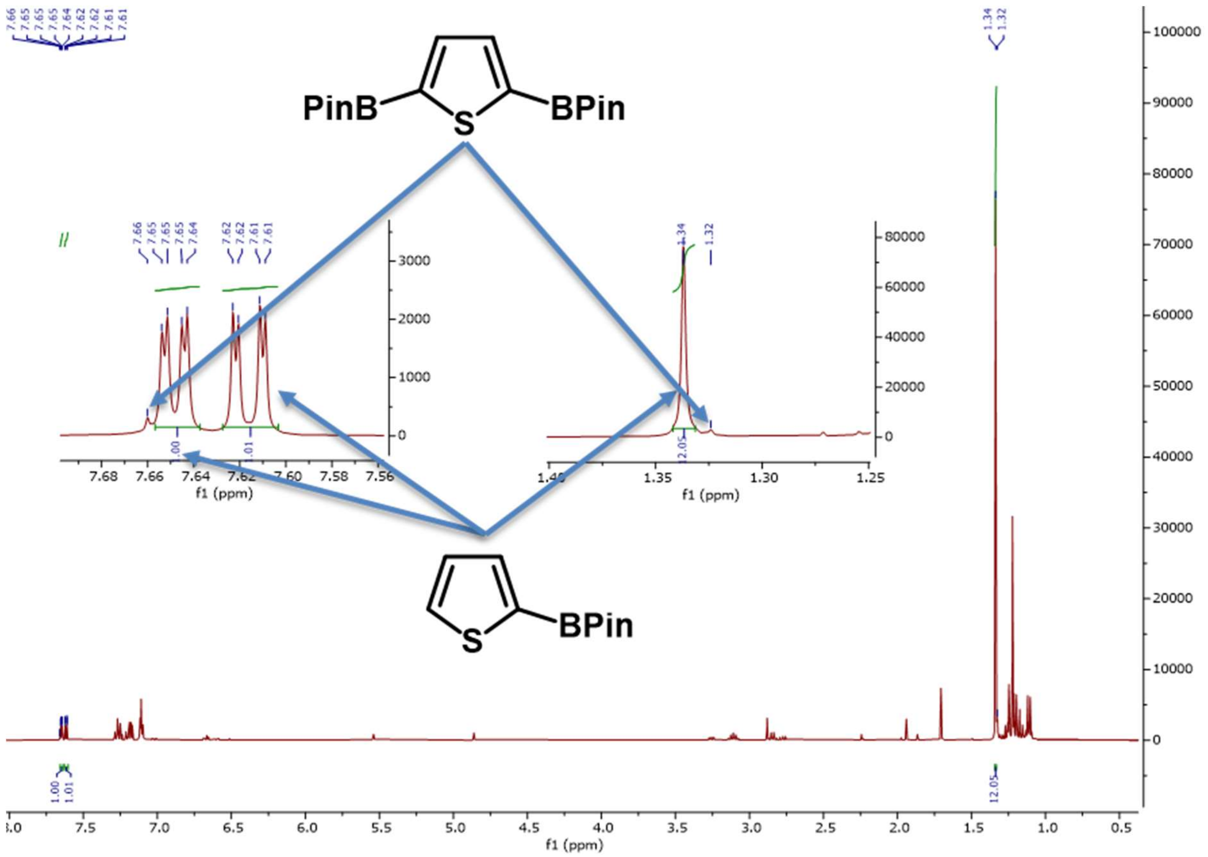

Figure S14: ^{1}H NMR Spectrum ($CDCl_3$) of crude BPin reaction mixture after filtration.

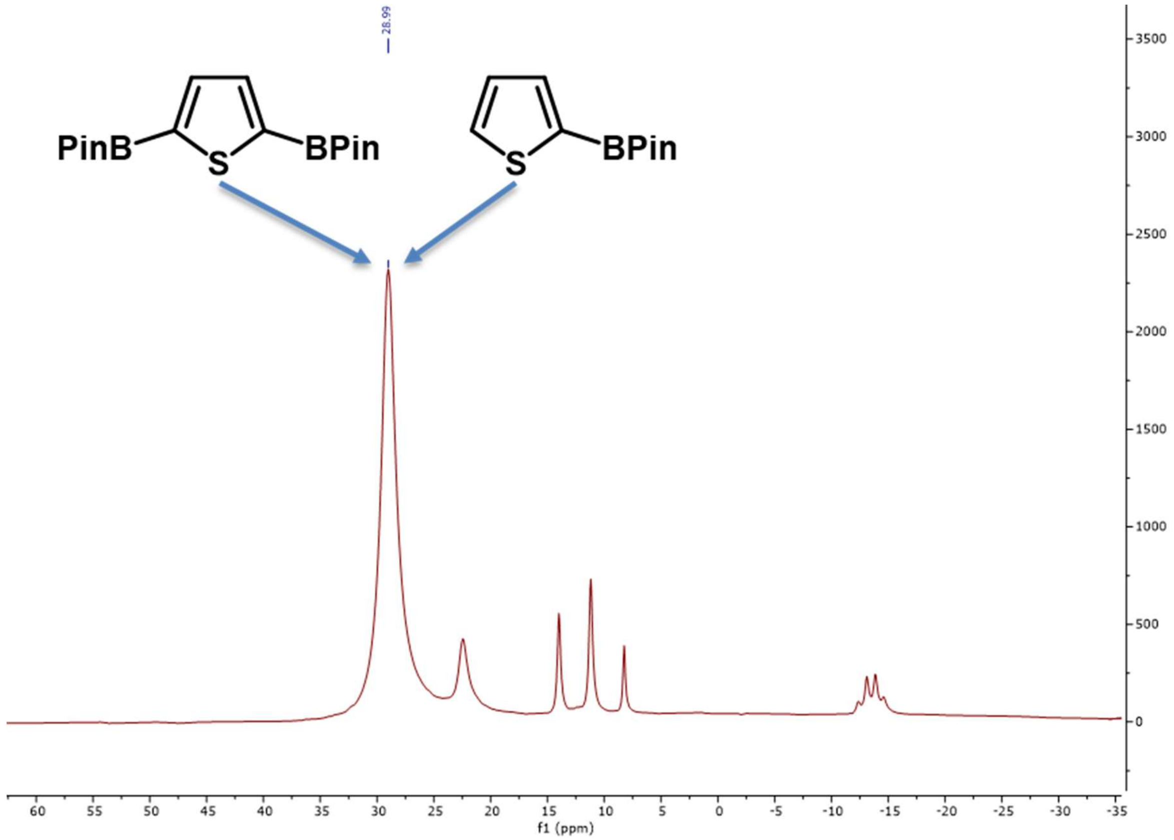

Figure S15: ^{11}B NMR Spectrum ($CDCl_3$) of crude BPin reaction mixture after filtration.

Borylation of 2-bromothiophene

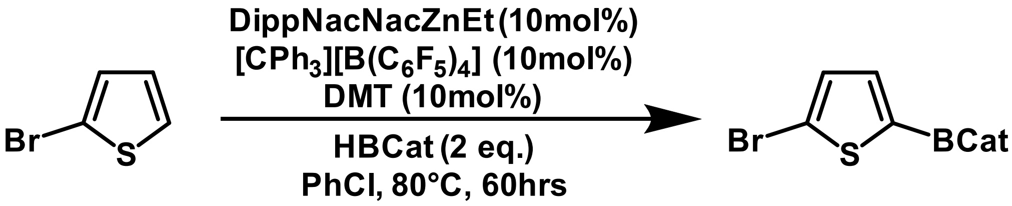

Yield: 77% by integration against an internal standard.

The borylated product was synthesised according to general procedure A. Conversion was determined relative to CH_2Br_2 as internal standard (35 µL, 0.5 mmol). Conversion to the boronic acid pinacol ester then was achieved by drying of the reaction mixture *in vacuo* before dissolving in DCM (0.5 mL) and reacting with 1M solution of pinacol in NEt_3 (0.75 mL, 0.75 mmol) for 18 hrs at room temperature. The reaction mixture was then extracted using (ca. 5mL) a 9:1 mixture of pentane:ethyl acetate and filtered through a (ca. 5cm) silica plug, further (ca. 5mL) pentane:ethyl acetate solution was used to ensure the product eluted from the plug. The eluent was then dried *in vacuo* and redissolved in $CDCl_3$ allowing confirmation of the initial product as 2-bromo-5-(1,3,2-benzodioxaborole)-thiophene by comparison to previously reported literature.[11]

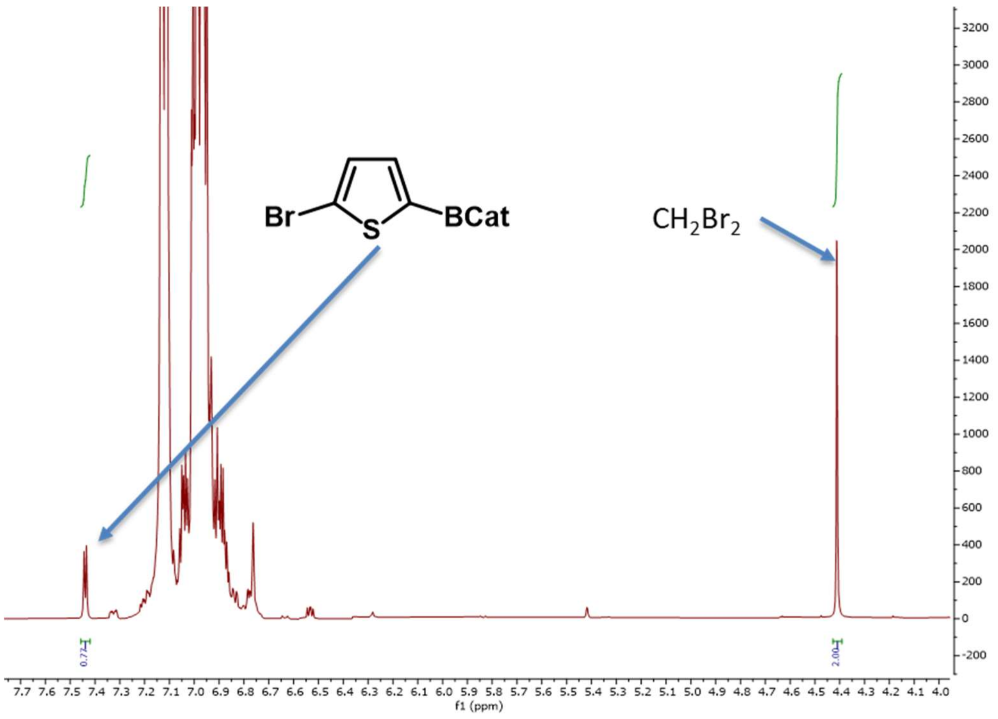

Figure S16: Borylation of 2-bromothiophene in PhCl with CH_2Br_2 internal standard as observed by ^1H NMR spectroscopy.

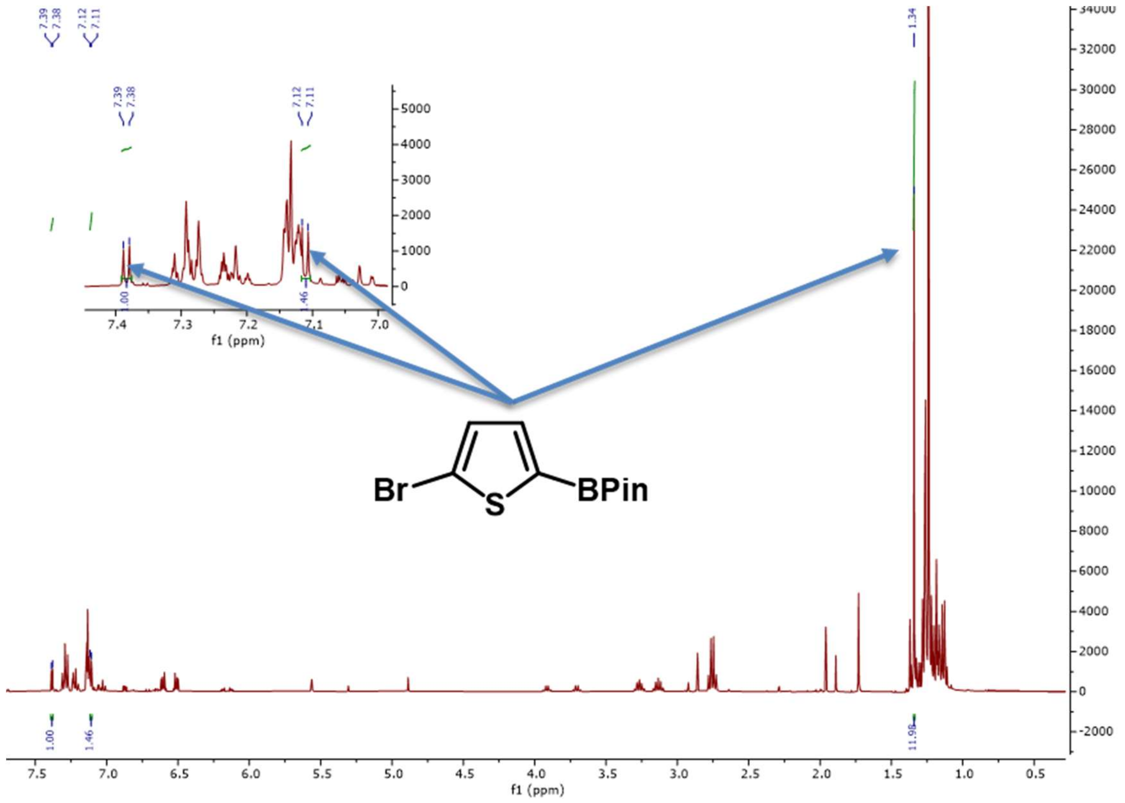

Figure S17: ^{1}H NMR Spectrum ($CDCl_3$) of crude BPin reaction mixture after filtration.

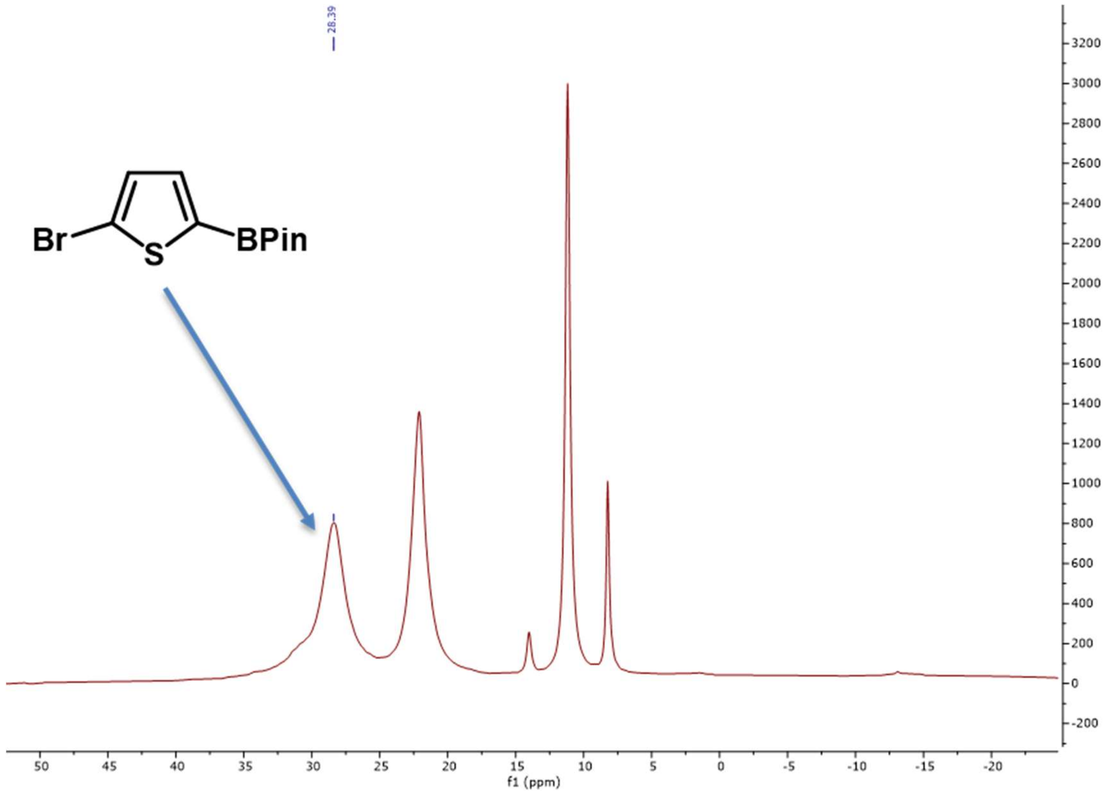

Figure S18: ^{11}B NMR Spectrum ($CDCl_3$) of crude BPin reaction mixture after filtration.

Borylation of *N*-methylindole

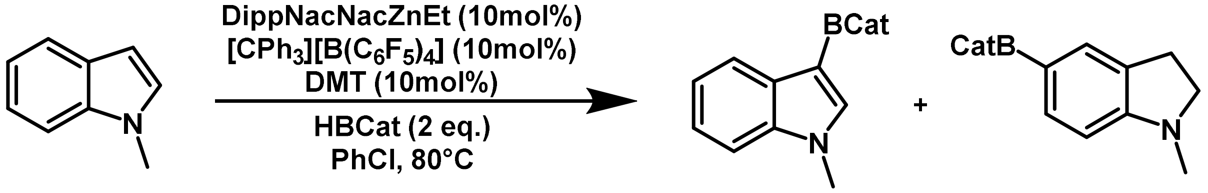

Yield: 85% (after 10mins) or >99% (after 1 hour, 55:45 3-BCat-indole:5-BCat-indoline) by integration against an internal standard.

Product was synthesised according to general procedure A. Yields were examined *in-situ* relative to an internal standard.

Two reactions were run with 10 min and 1 hour reaction time, the latter of which was converted to the boronic acid pinacol ester. Conversion to the boronic acid pinacol ester was achieved by drying of the reaction mixture *in vacuo* before dissolving in DCM (0.5 mL) and reacting with 1M solution of pinacol in NEt_3 (0.75 mL, 0.75 mmol) for 18 hrs at room temperature. The reaction mixture was then extracted using (ca. 5 mL) a 9:1 mixture of pentane:ethyl acetate and filtered through a (ca. 5 cm) silica plug, further (ca. 5 mL) pentane:ethyl acetate solution was used to ensure the product passed through the plug. The eluent was then dried *in vacuo* and redissolved in $CDCl_3$ allowing confirmation of the initial products as *N*-methyl-3-(1,3,2-benzodioxaborole)-indole and *N*-methyl-5-(1,3,2-benzodioxaborole)-indoline by comparison to previously reported literature.[12,13] Product ratio after silica plug is 39:61 3-BCat-indole:5-BCat-indoline due to partial proto-deborylation (formation of *N*-methyl-indole observed by NMR)

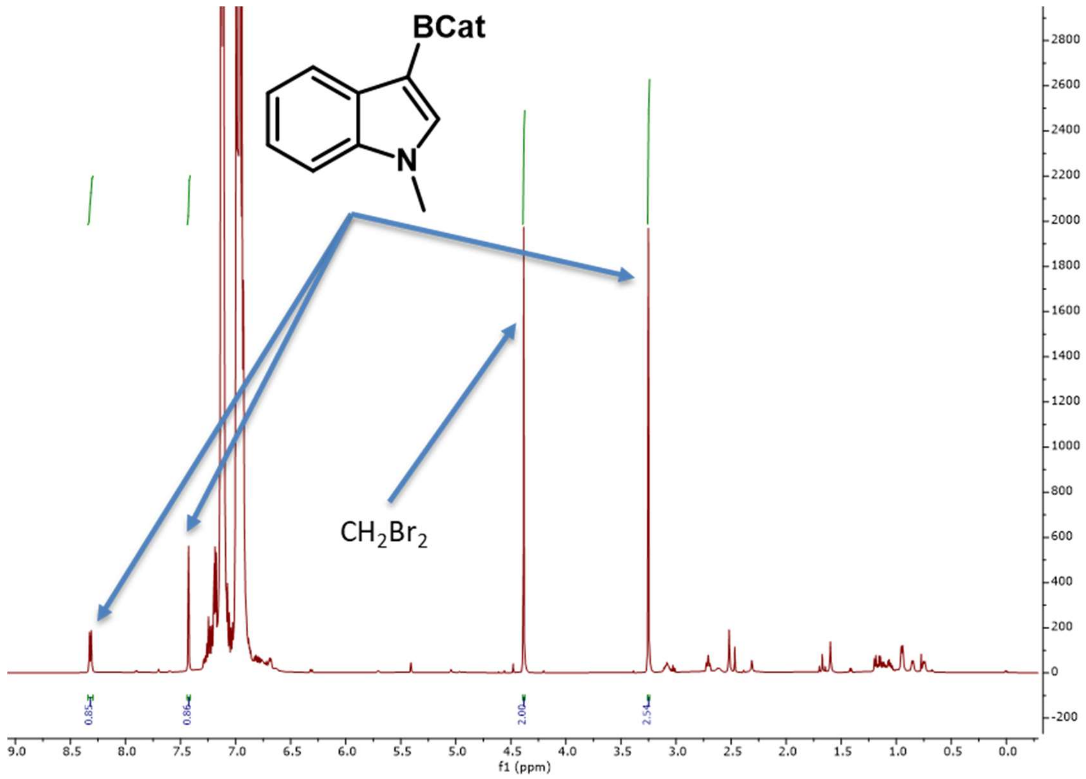

Figure S19: Borylation of *N*-methylindole in PhCl after 10 mins at 80°C with CH_2Br_2 internal standard as observed by ^1H NMR spectroscopy.

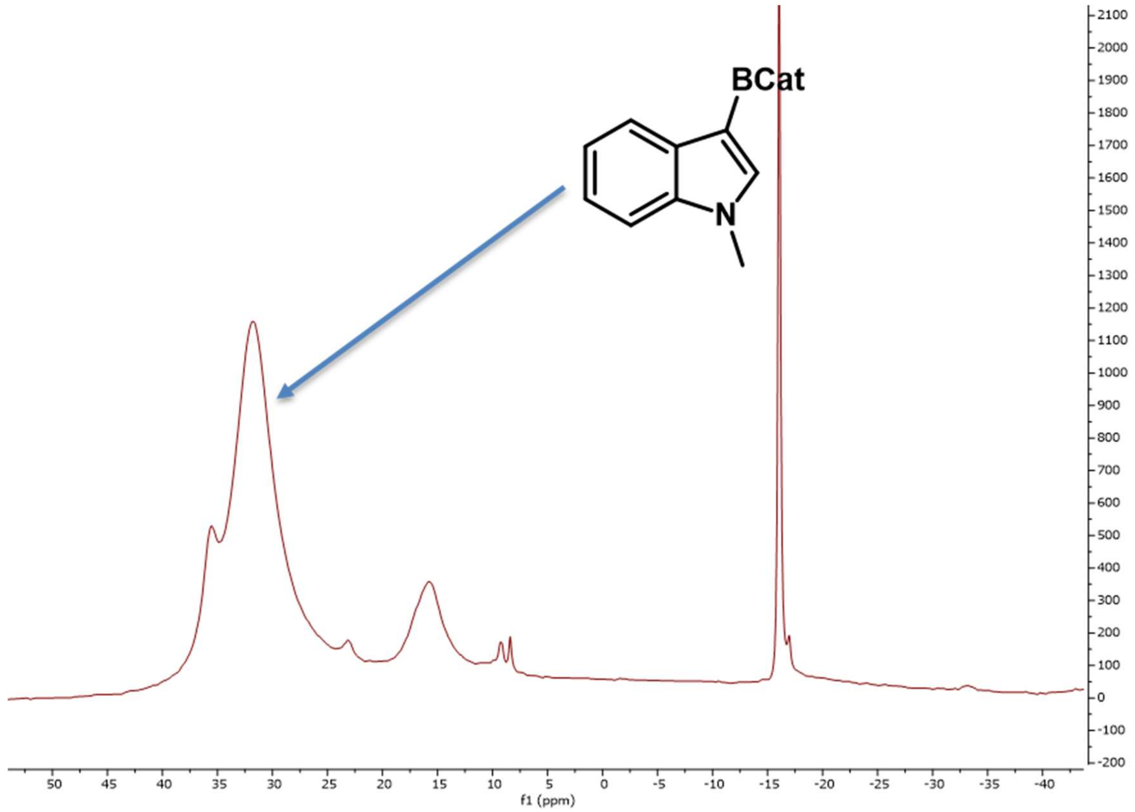

Figure S20: Borylation of *N*-methylindole in PhCl after 10 mins at 80°C as observed by ^{11}B NMR spectroscopy.

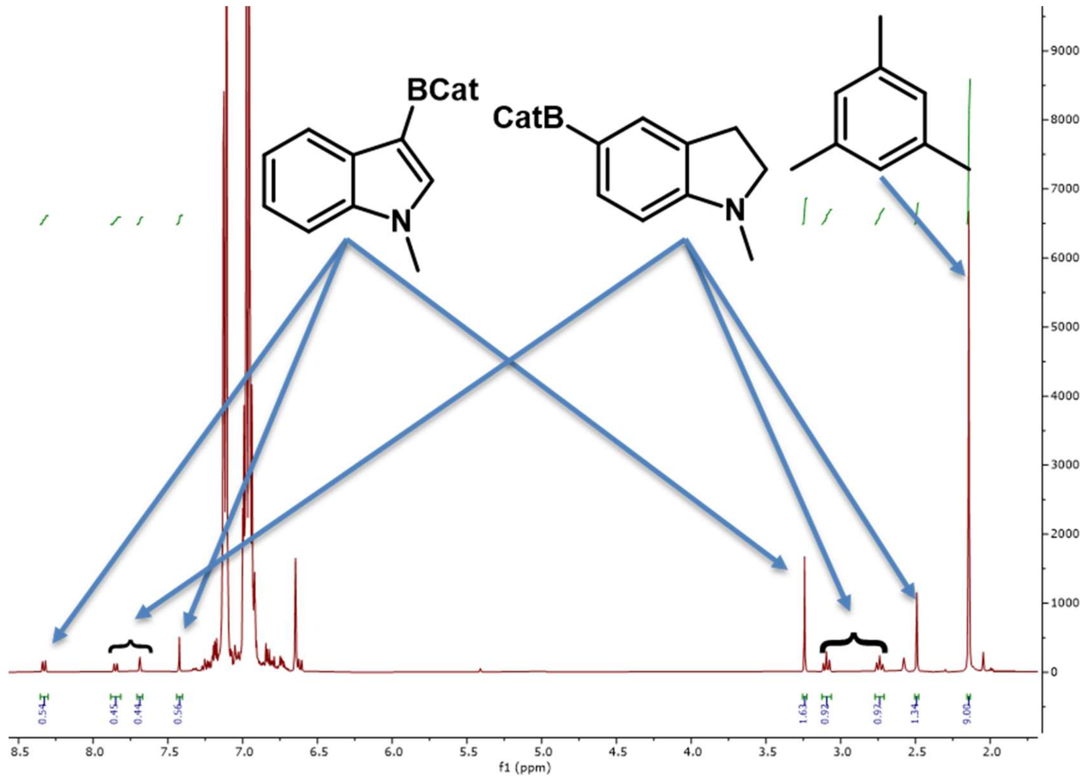

Figure S21: Borylation of *N*-methylindole in PhCl after 1 hr at 80°C with mesitylene internal standard as observed by ^{1}H NMR spectroscopy.

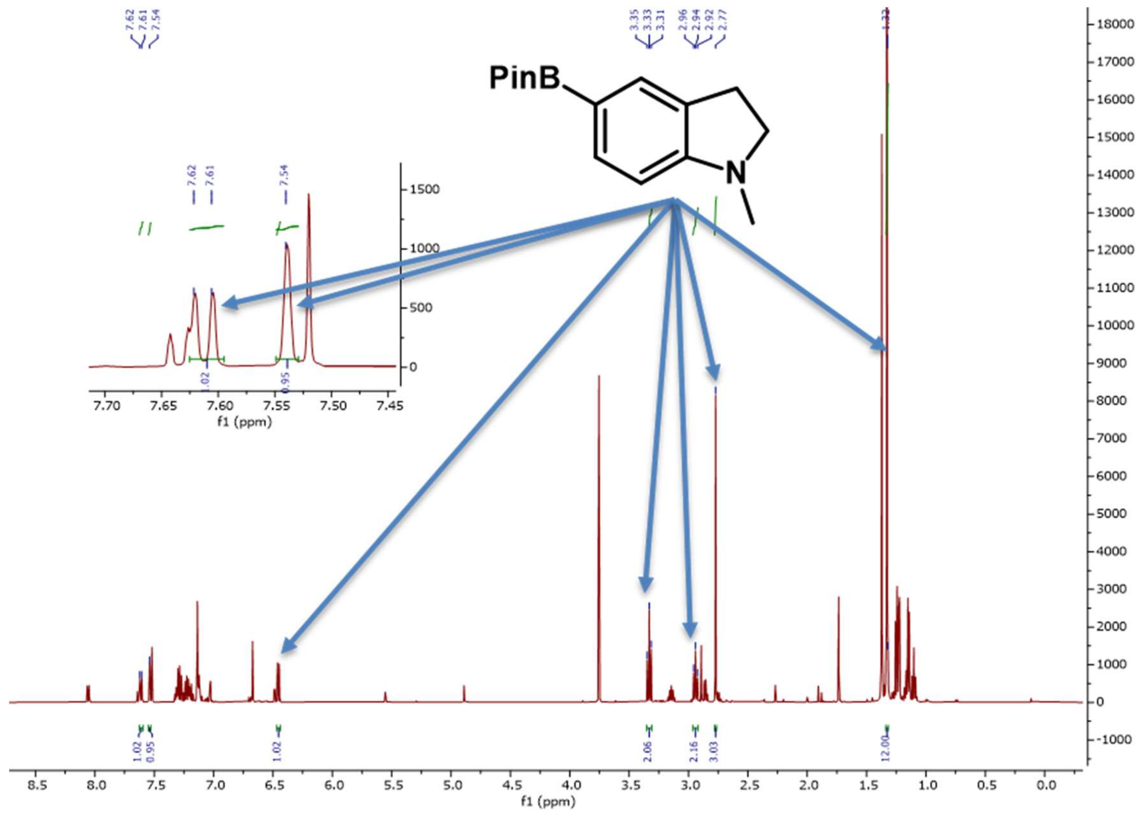

Figure S22: ^{1}H NMR Spectrum ($CDCl_3$) of crude BPin reaction mixture after filtration highlighting formation of *N*-methyl-5-(4,4,5,5-tetramethyl-1,3,2-dioxaborolan-2-yl)-indoline.

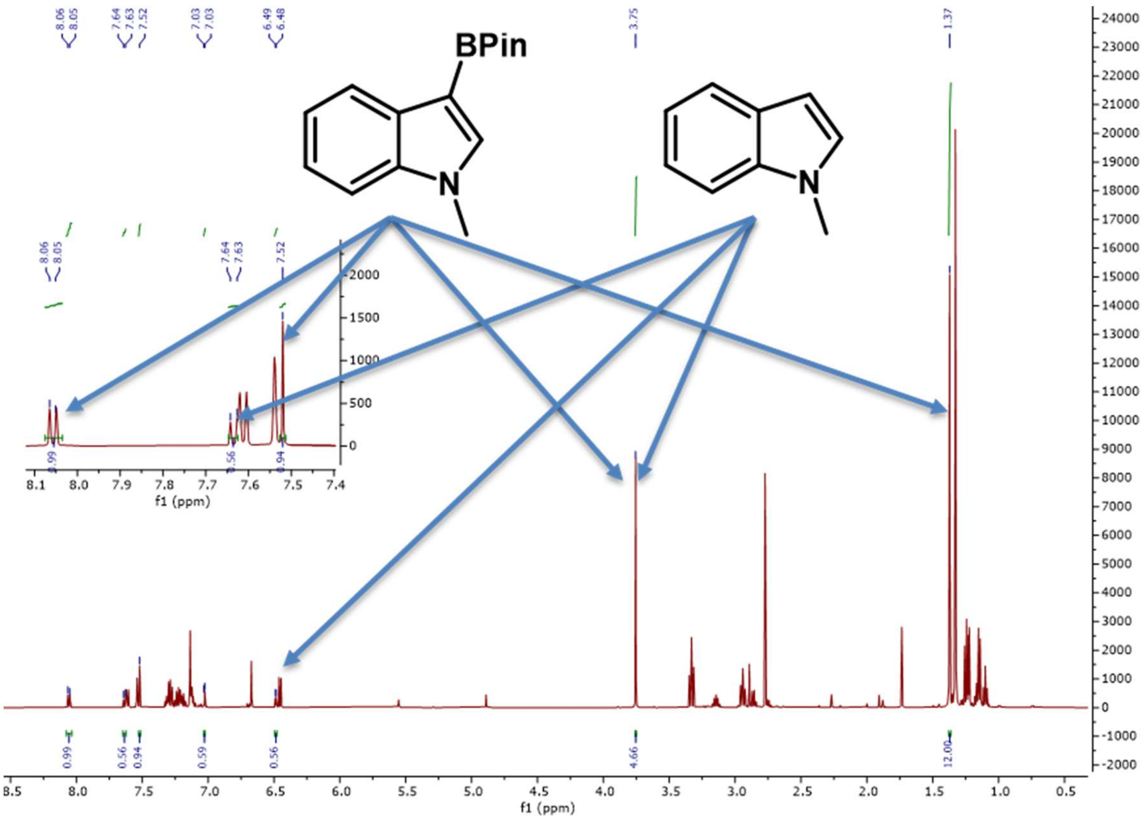

Figure S23: ^{1}H NMR Spectrum ($CDCl_3$) of crude BPin reaction mixture after partial work-up/filtration highlighting formation of *N*-methyl-3-(4,4,5,5-tetramethyl-1,3,2-dioxaborolan-2-yl)-indole and proto-deborylation product.

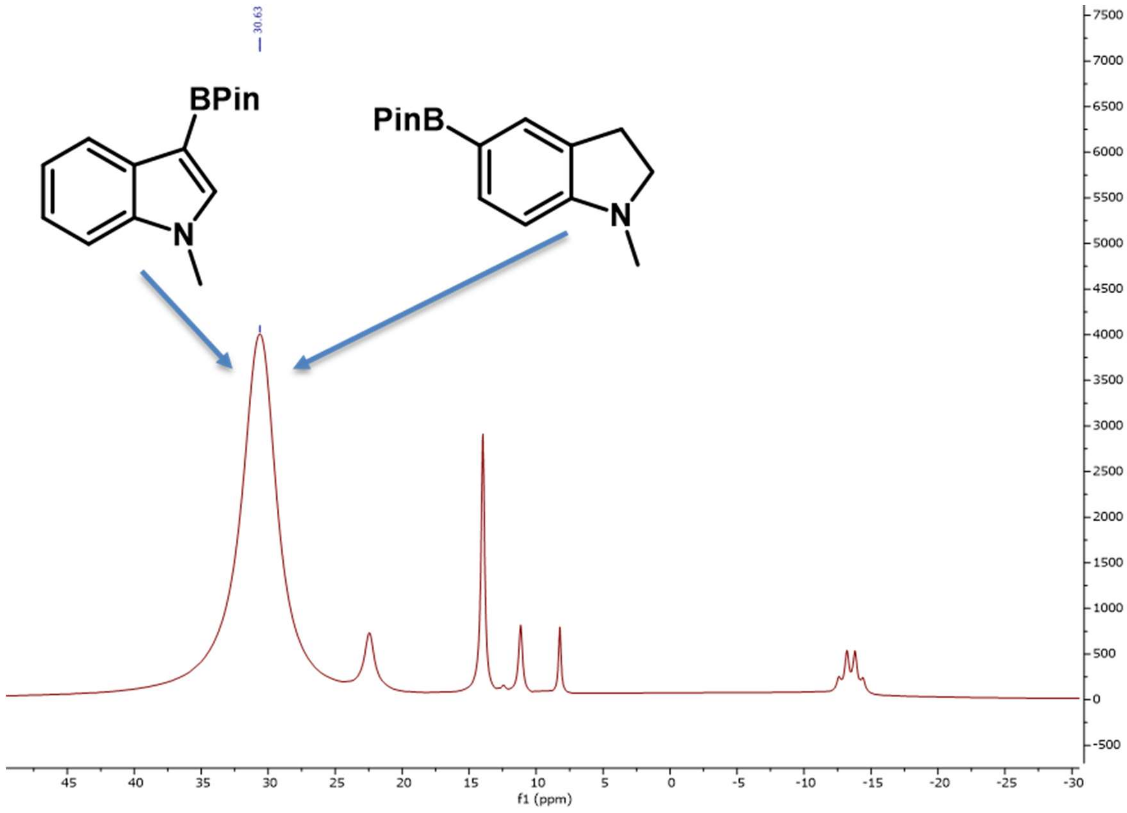

Figure S24: ^{11}B NMR Spectrum ($CDCl_3$) of crude BPin reaction mixture after partial work-up/filtration.

Borylation of *N,N*-dimethylaniline

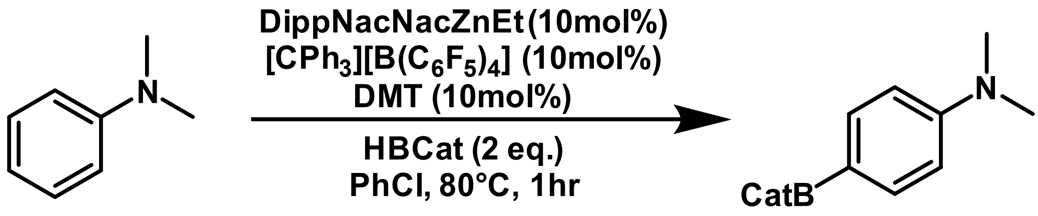

Yield: 99% by integration against an internal standard.

Product was synthesised according to general procedure A. Conversion was determined *in-situ* by comparison with mesitylene internal standard (70 µL, 0.5 mmol). Conversion to the boronic acid pinacol ester then was achieved by drying of the reaction mixture *in vacuo* before dissolving in DCM (0.5 mL) and reacting with 1M solution of pinacol in NEt_3 (0.75 mL, 0.75 mmol) for 18 hrs at room temperature. The reaction mixture was then extracted using (ca. 5 mL) a 9:1 mixture of pentane:ethyl acetate and filtered through a (ca. 5 cm) silica plug, further (ca. 5 mL) pentane:ethyl acetate solution was used to ensure the product eluted from the plug. The eluent was then dried *in vacuo* and redissolved in $CDCl_3$ allowing confirmation of the initial product as *N,N*-dimethyl-4-(1,3,2-benzodioxaborole)-aniline by comparison to previously reported literature.[14]

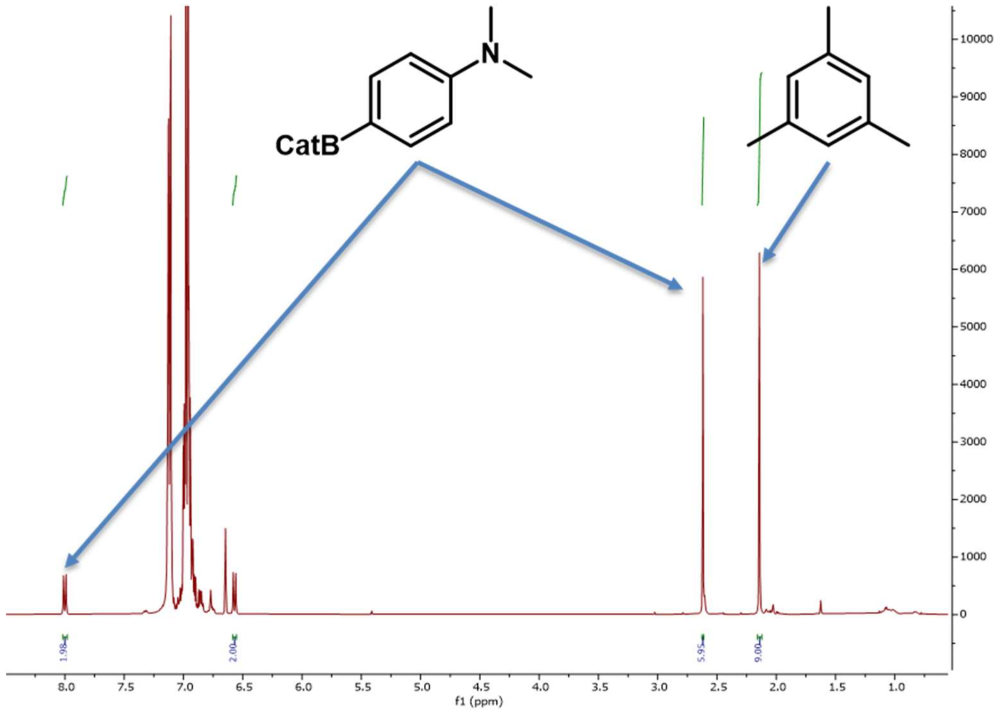

Figure S25: Borylation of *N,N*-dimethylaniline in PhCl with mesitylene internal standard as observed by ^{1}H NMR spectroscopy.

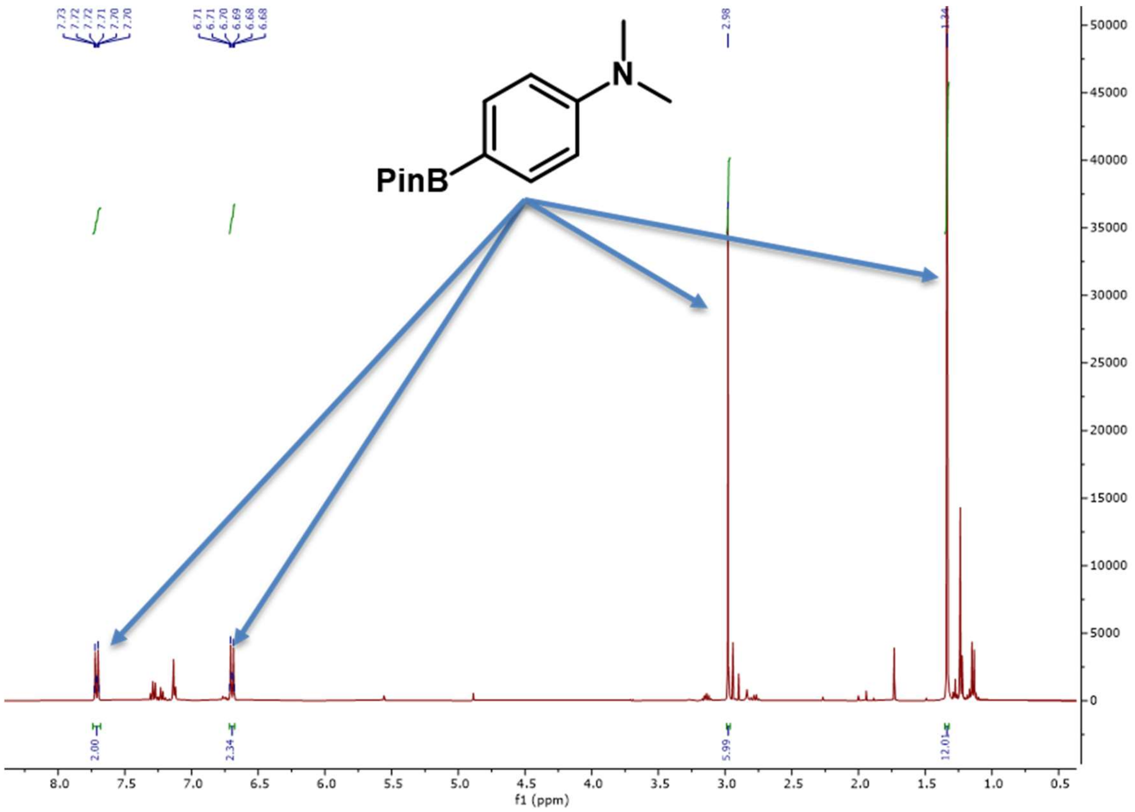

Figure S26: ^{1}H NMR Spectrum ($CDCl_3$) of crude BPin reaction mixture after filtration.

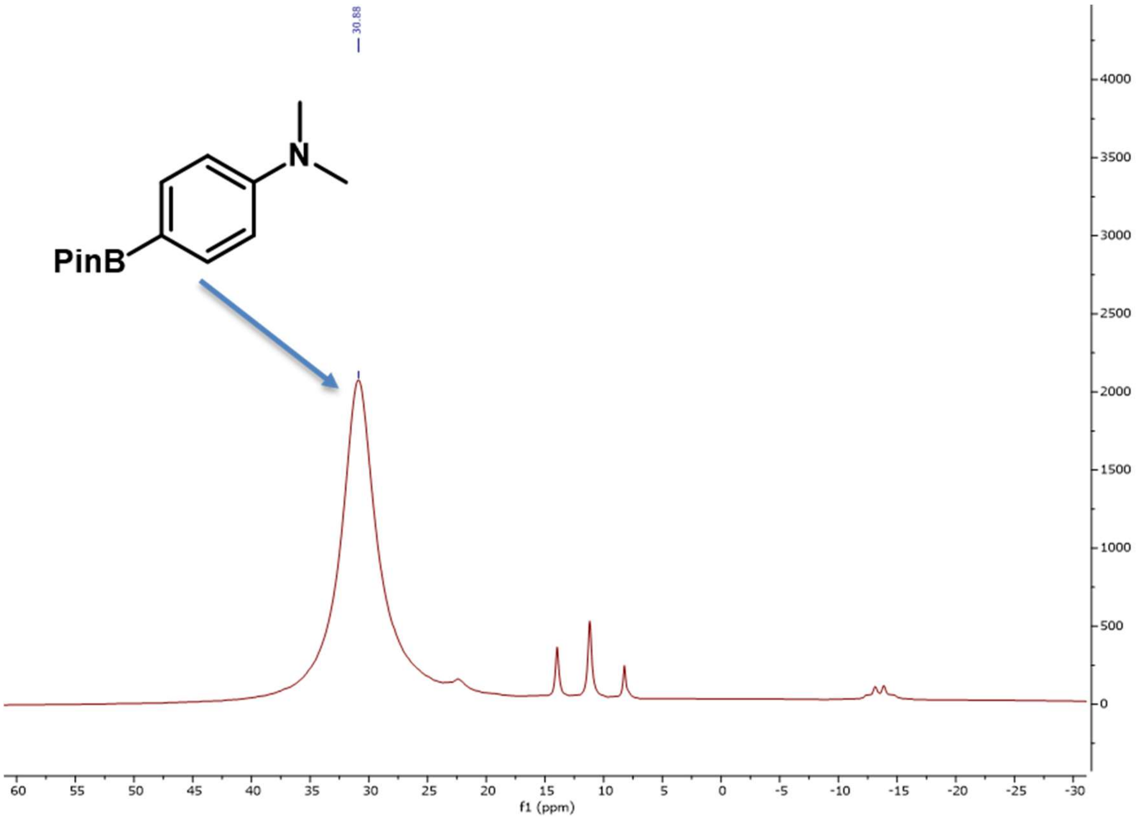

Figure S27: ^{11}B NMR Spectrum ($CDCl_3$) of crude BPin reaction mixture after filtration.

Borylation of Furan

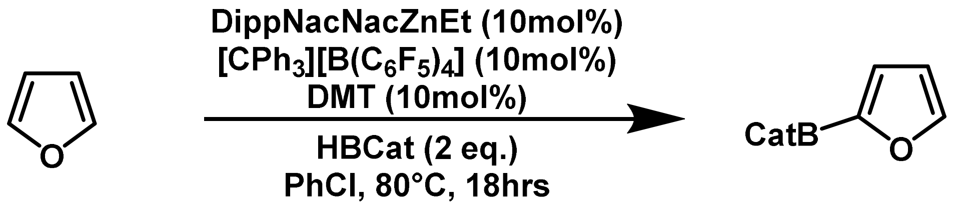

Yield: 94% (ca. 85:9 ratio of 2-BCat:3-BCat Furan) by integration against an internal standard.

Product was synthesised according to general procedure A. Conversion was examined *in-situ* by comparison with mesitylene internal standard (70 µL, 0.5 mmol). Conversion to the boronic acid pinacol ester was achieved by drying of the reaction mixture *in vacuo* before dissolving in DCM (0.5 mL) and reacting with 1M solution of pinacol in NEt_3 (0.75 mL, 0.75 mmol) for 18 hrs at room temperature. The reaction mixture was then extracted using (ca. 5mL) a 9:1 mixture of pentane:ethyl acetate and filtered through a (ca. 5cm) silica plug, further (ca. 5mL) pentane:ethyl acetate solution was used to ensure the product passed through the plug. The eluent was then dried *in vacuo* and redissolved in $CDCl_3$ allowing confirmation of the initial major product as 2-(1,3,2-benzodioxaborole)-furan by comparison to previously reported literature.[10] However alongside the 2-borylated isomer other borylated isomers were observed, possibly including the 2,5- and 2,4-diborylated isomers, but the 2 borylated isomer was the dominant product (85% conversion).

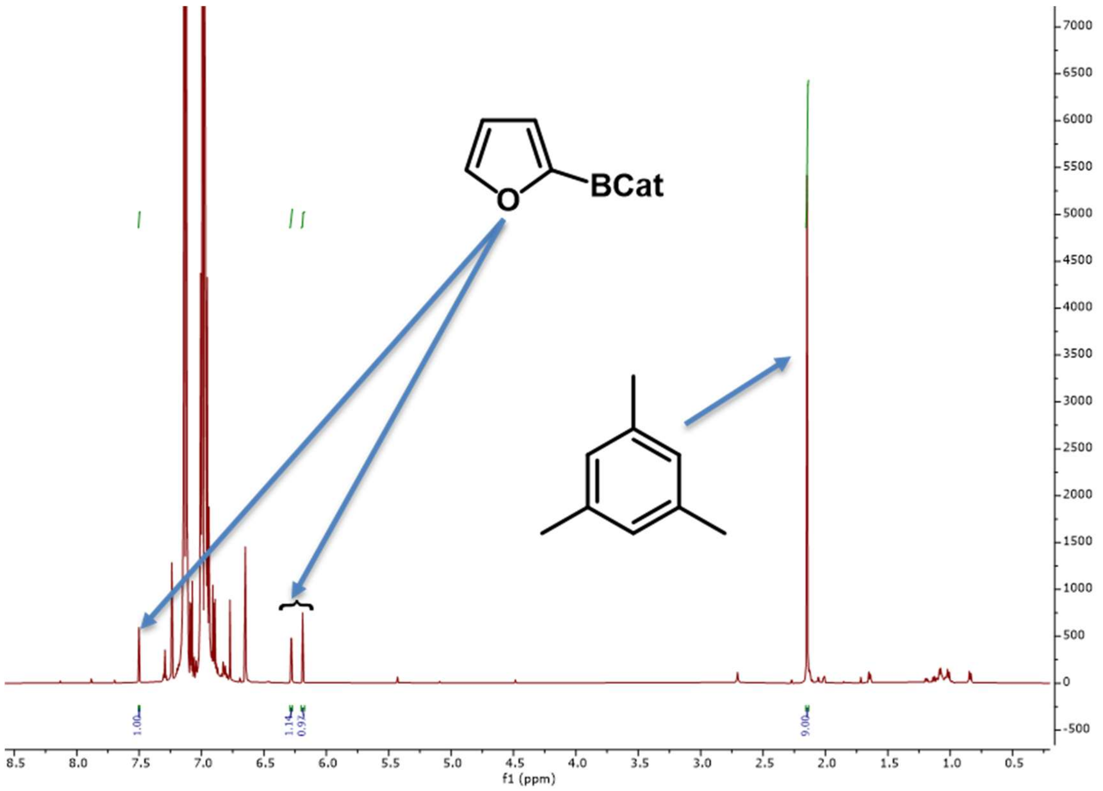

Figure S28: Borylation of Furan in PhCl with mesitylene internal standard as observed by ^{1}H NMR spectroscopy.

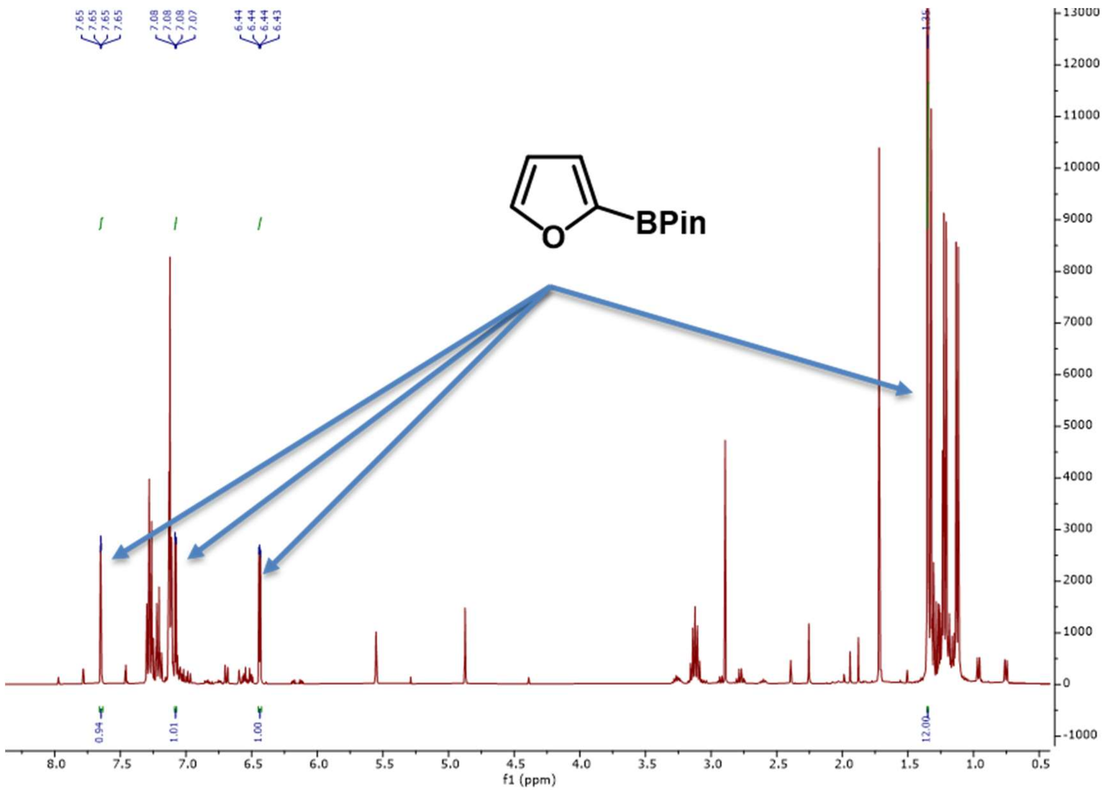

Figure S29: ^{1}H NMR Spectrum ($CDCl_3$) of crude BPin reaction mixture after filtration highlighting major 2-(1,3,2-benzodioxaborole)-furan.

Borylation of *N*-methylpyrrole

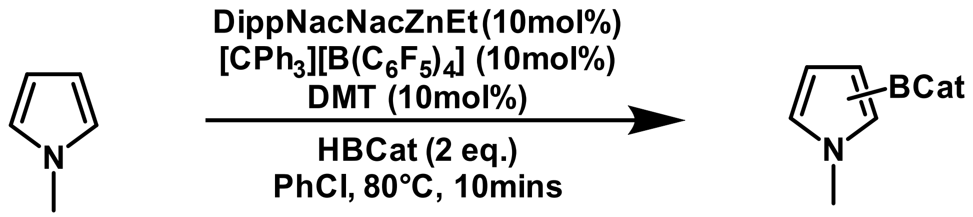

Yield: 85% (56:29 2-:3-BCat pyrrole) by integration against an internal standard.

Product was synthesised according to general procedure A. Conversion was examined *in-situ* by comparison with mesitylene internal standard (70 µL, 0.5 mmol). Products were confirmed as 2- and 3-(1,3,2-benzodioxaborole)-*N*-methylpyrrole by comparison to previously reported literature.[15]

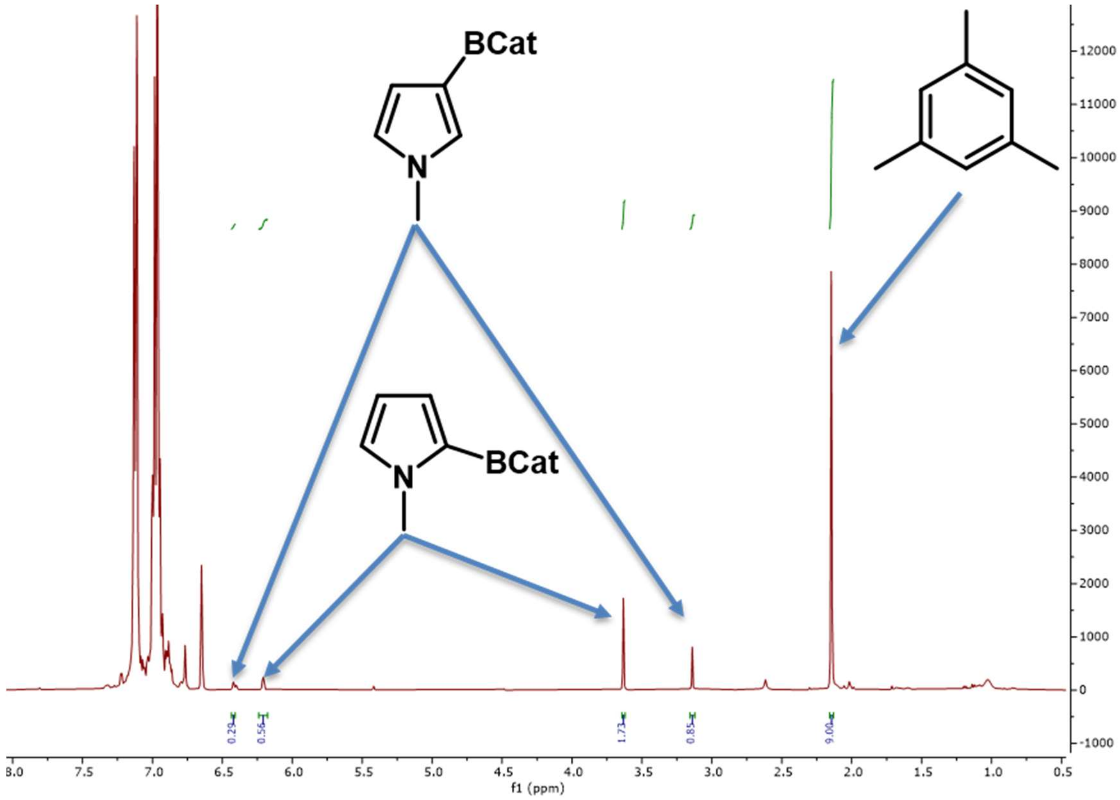

Figure S30: Borylation of *N*-methylpyrrole in PhCl with mesitylene internal standard as observed by ^{1}H NMR spectroscopy.

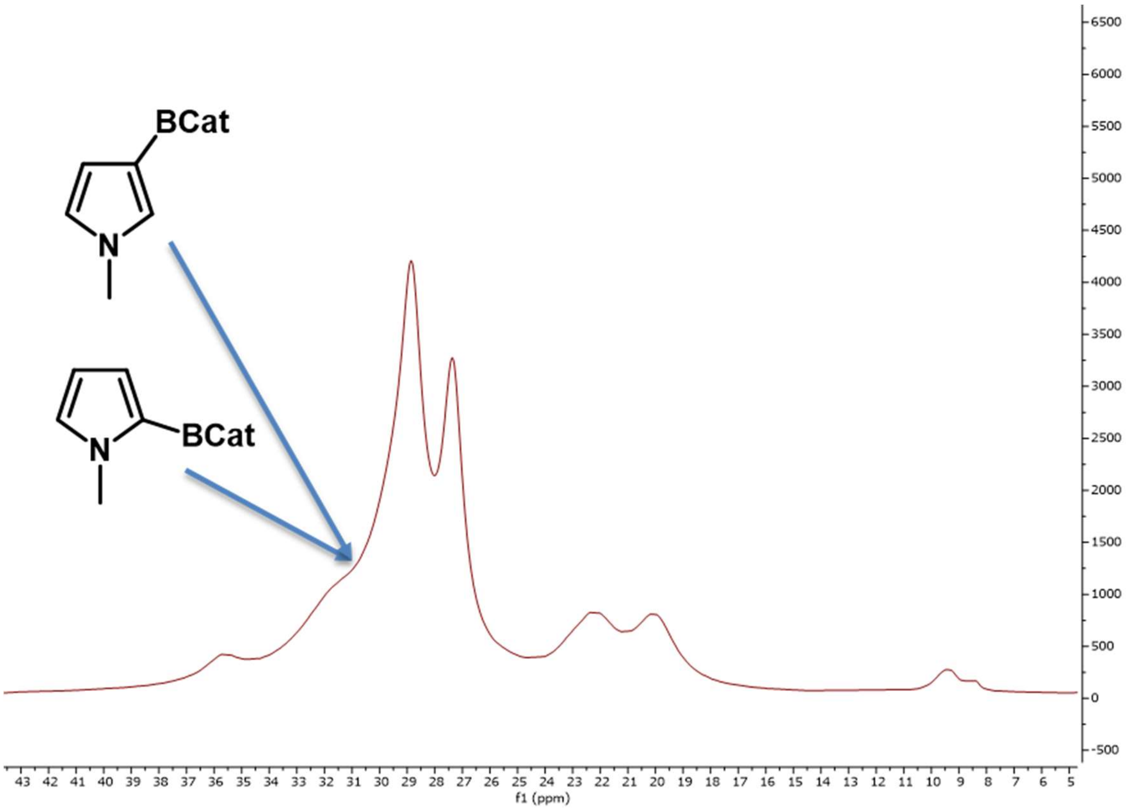

Figure S31: Borylation of *N*-methylpyrrole in PhCl with mesitylene internal standard as observed by ^{11}B NMR spectroscopy.

Borylation of benzothiophene

With *N,N*-dimethyl-4-toluidine

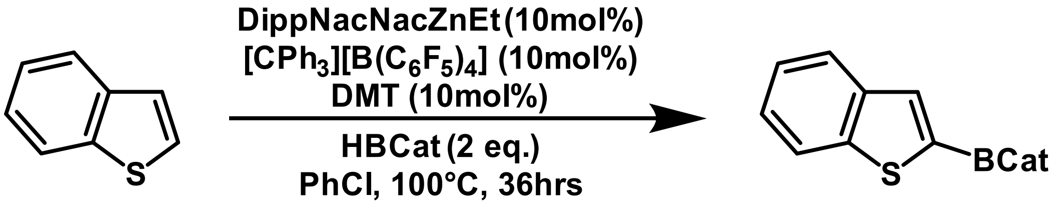

Yield: 37% by integration against an internal standard.

Product was synthesised according to general procedure A. Conversion was examined relative to CH_2Br_2 internal standard (35 µL, 0.5 mmol). Conversion to the boronic acid pinacol ester was achieved by drying of the reaction mixture *in vacuo* before dissolving in DCM (0.5 mL) and reacting with 1M solution of pinacol in NEt_3 (0.75 mL, 0.75 mmol) for 18 hrs at room temperature. The reaction mixture was then extracted using (ca. 5mL) a 9:1 mixture of

pentane:ethyl acetate and filtered through a (ca. 5cm) silica plug, further (ca. 5mL) pentane:ethyl acetate solution was used to ensure the product eluted from the plug. The eluent was then dried *in vacuo* and redissolved in $CDCl_3$ allowing confirmation of the initial product as 2-bromo-5-(1,3,2-benzodioxaborole)-thiophene by comparison to previously reported literature.[16]

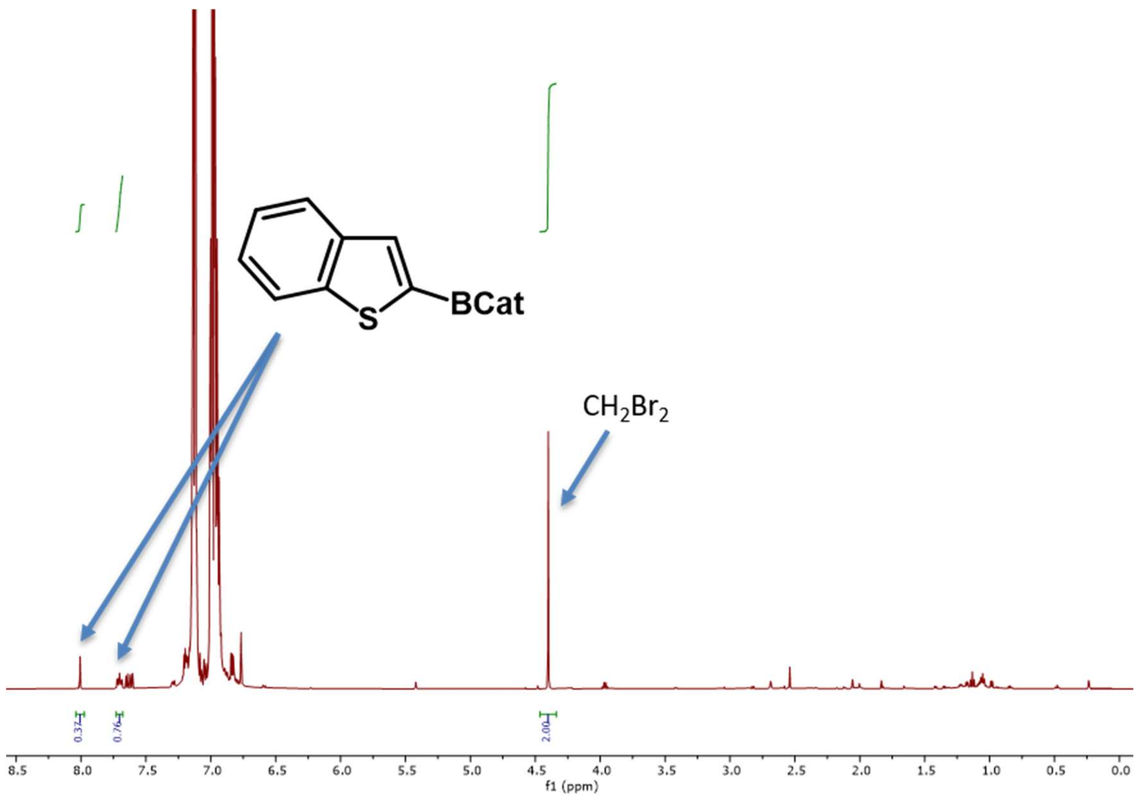

Figure S32: Borylation of benzothiophene in PhCl with CH_2Br_2 internal standard as observed by ^1H NMR spectroscopy.

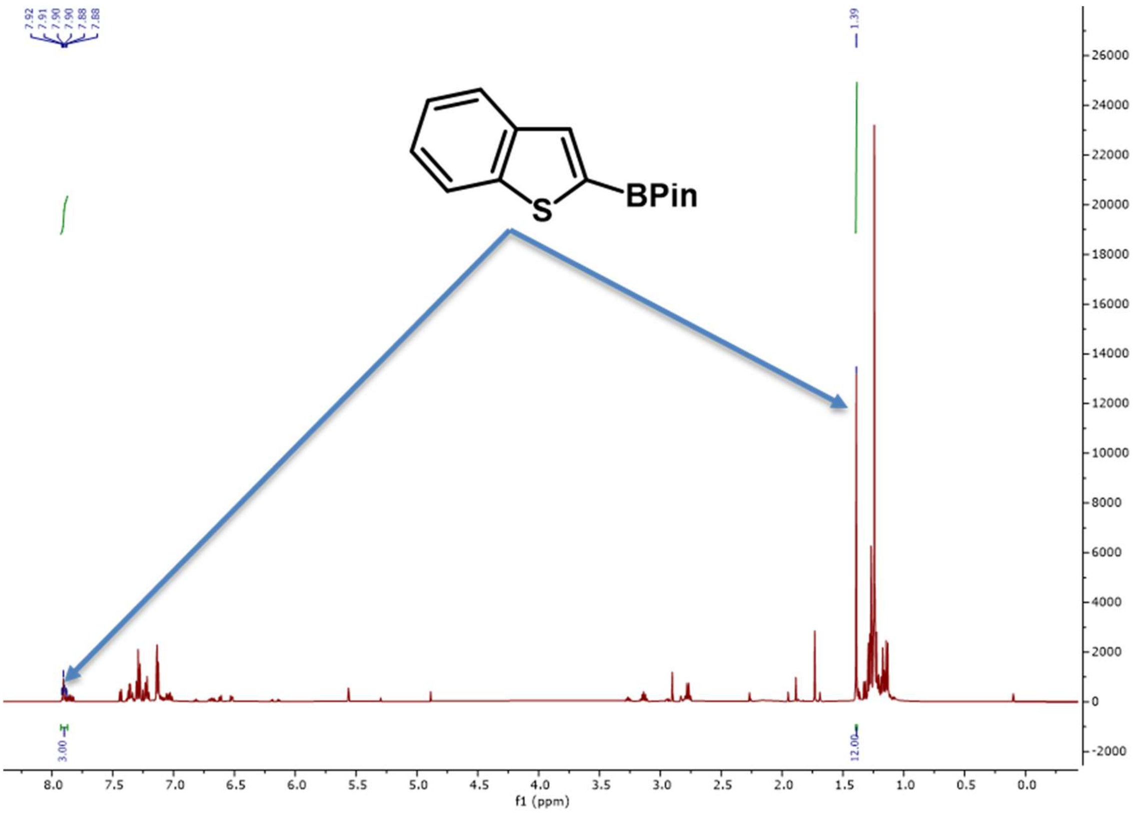

Figure S33: ^{1}H NMR Spectrum ($CDCl_3$) of crude BPin reaction mixture after filtration.

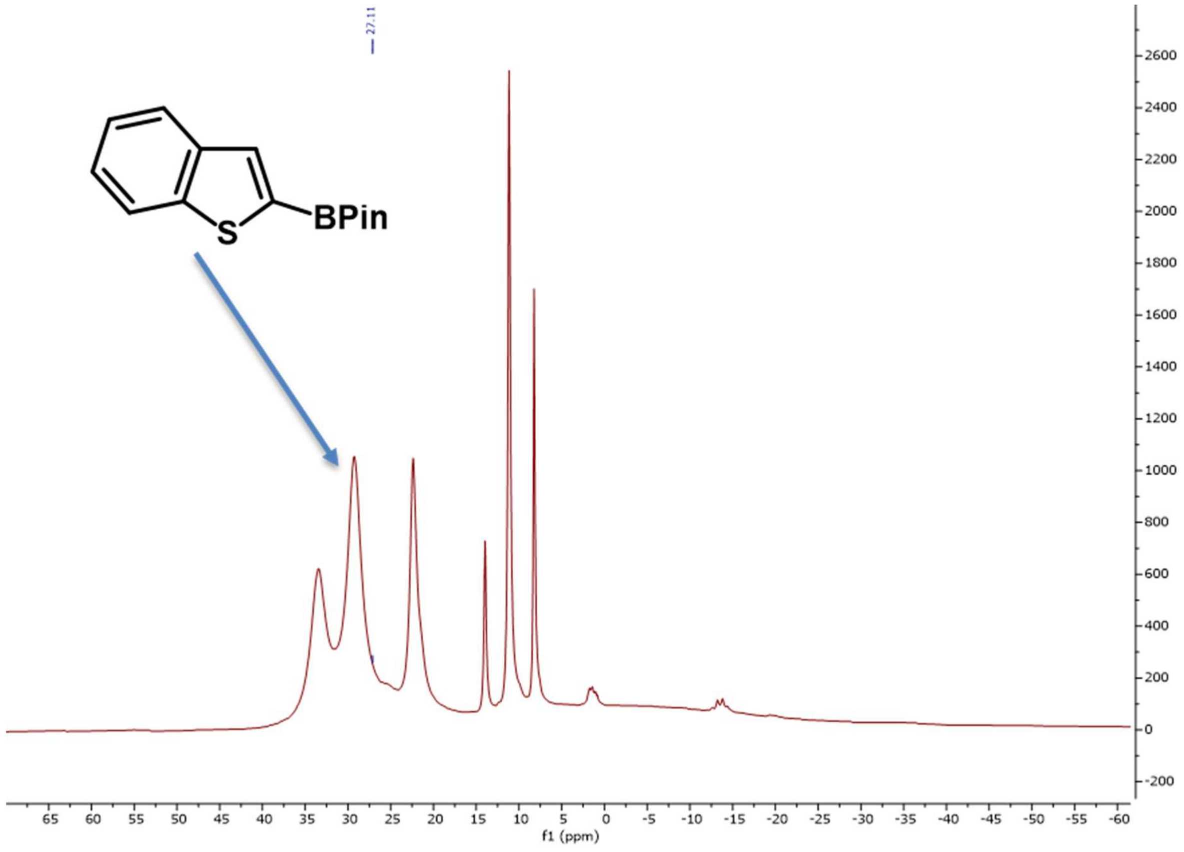

Figure S34: ^{11}B NMR Spectrum ($CDCl_3$) of crude BPin reaction mixture after filtration.

With *N,N*-dimethyl-2,4-dibromoaniline

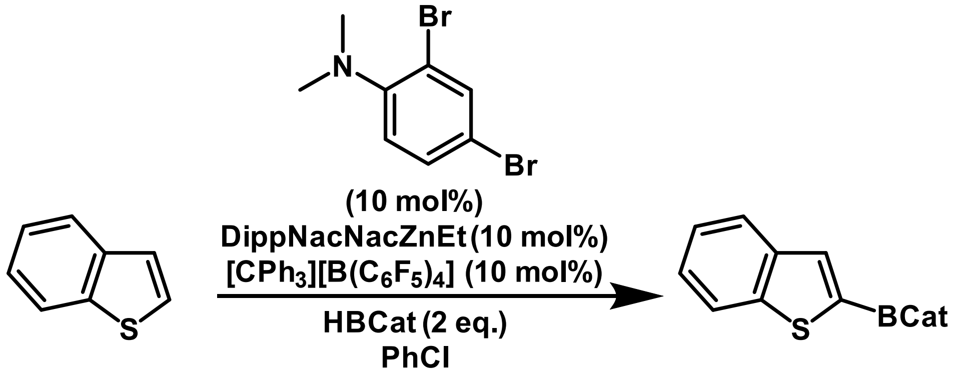

Yield: 17% (18 hours, 100°C), 26% (18 hours, 80°C) or 29% (36 hours, 80°C) by integration against an internal standard.

Product was synthesised according to general procedure A, however, *N,N*-dimethyl-2,4-dibromoaniline was used in place of *N,N*-dimethyl-4-toluidine, temperatures and reaction times were also altered as noted in figure titles. Addition of an internal standard, either dibromomethane (35 μL, 0.50 mmol) or mesitylene (70 μL, 0.50mmol), was done at the end of the reaction and used to determine *in-situ* conversions by integration of diagnostic ^{1}H resonances.

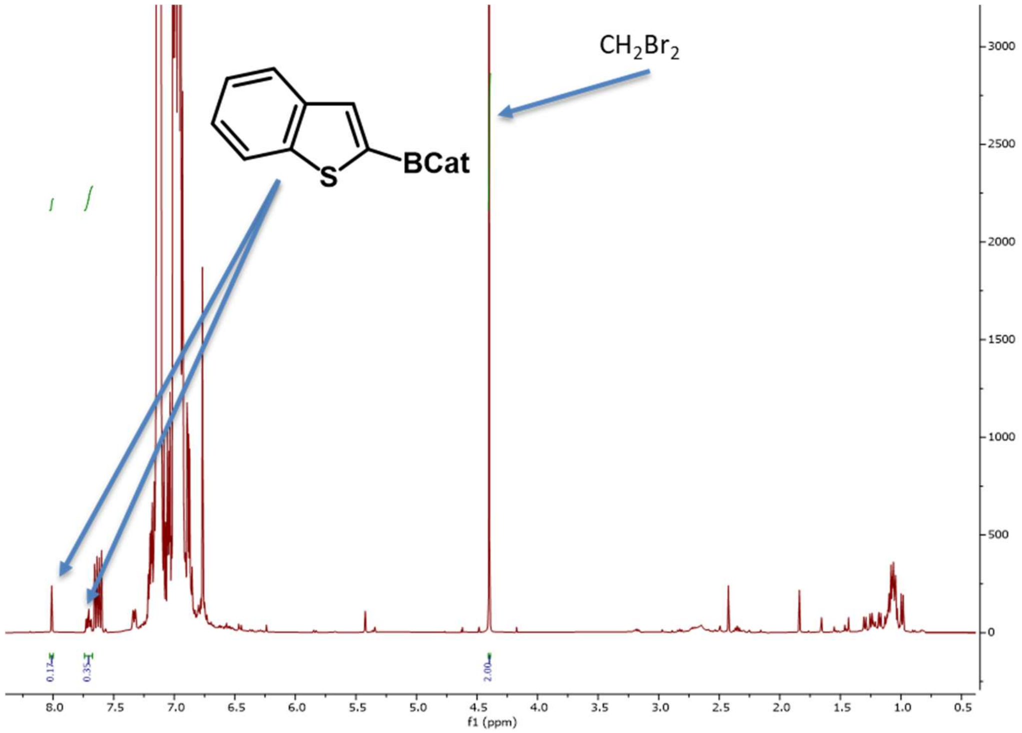

Figure S35: Borylation of benzothiophene in PhCl after 18 hours at 100°C with dibromomethane internal standard as observed by ^{1}H NMR spectroscopy.

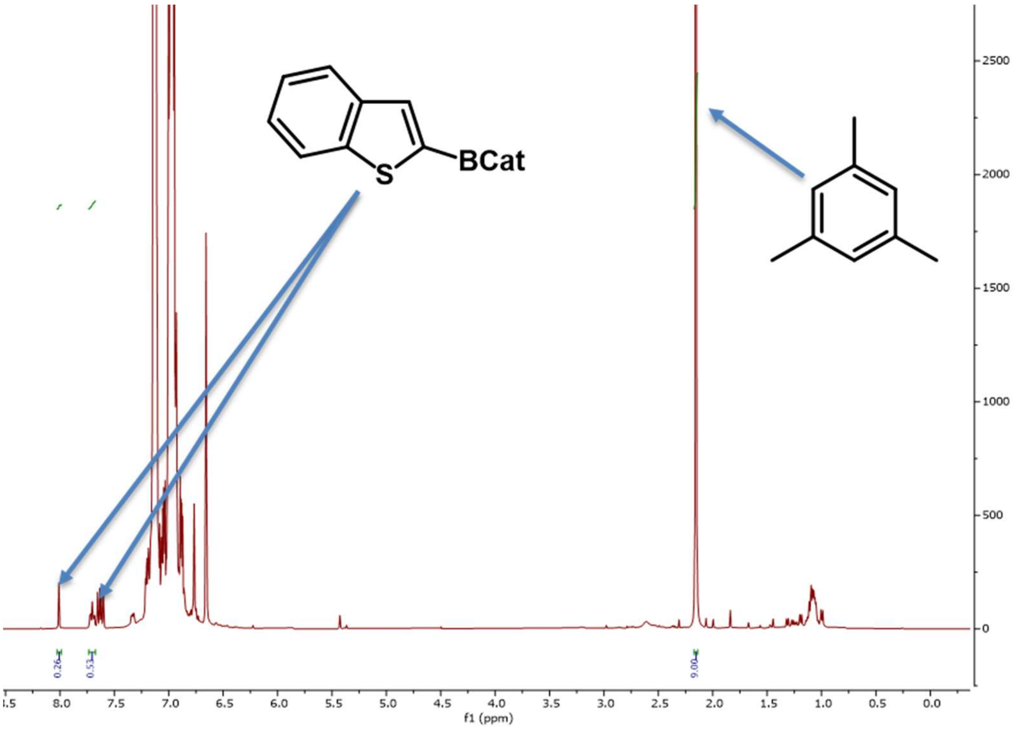

Figure S36: Borylation of benzothiophene in PhCl after 18 hours at 80°C with mesitylene internal standard as observed by ^{1}H NMR spectroscopy.

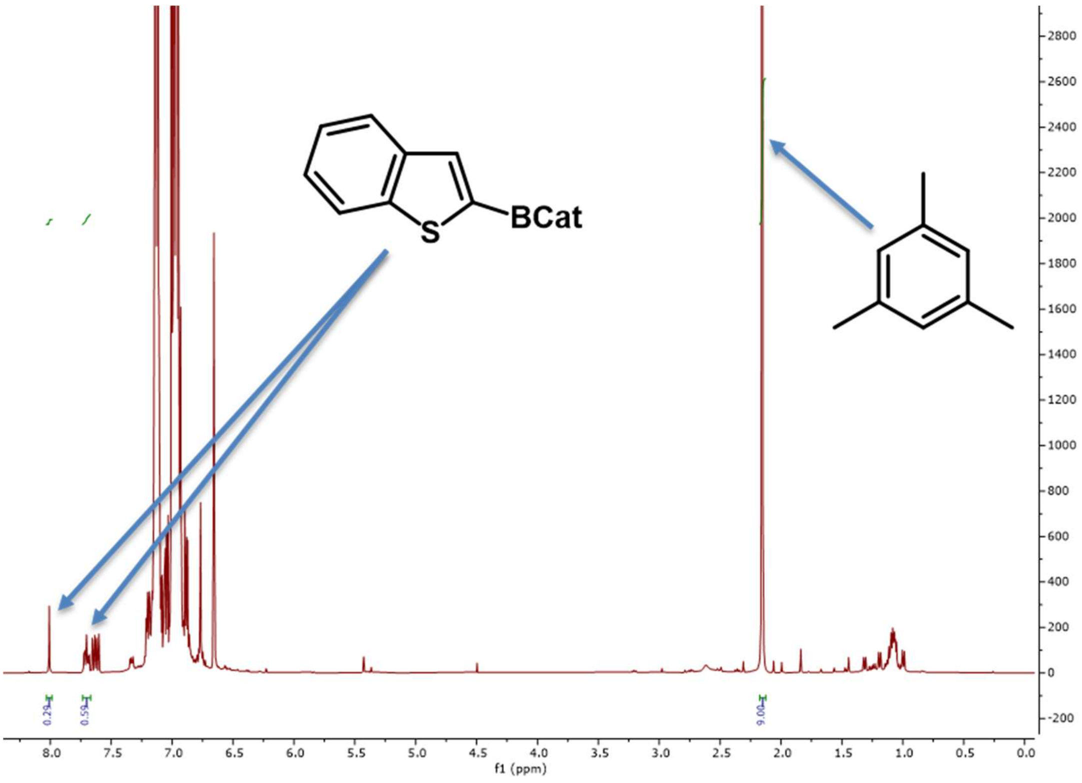

Figure S37: Borylation of benzothiophene in PhCl after 36 hours at 80°C with mesitylene internal standard as observed by ^{1}H NMR spectroscopy.

S5. Variation of Base

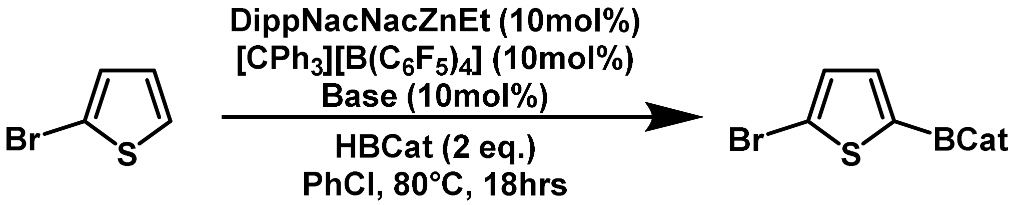

General procedure B: A J Young's NMR tube was charged with NacNacZnEt (26 mg, 0.05 mmol) and $[CPh_3][B(C_6F_5)_4]$ (46 mg, 0.05 mmol) before dissolution in PhCl (1 mL) and addition of a base (1.00 mmol), catecholborane (107 µL, 1.00mmol) and 2-bromothiophene (49 µL, 0.50 mmol). The reaction mixture was heated for 18 hours at 80°C and monitored by ^1H and ^{11}B NMR spectroscopy. Addition of dibromomethane (35 µL, 0.50 mmol) as an internal standard was done at the end of the reaction and used to determine *in-situ* yields by integration of diagnostic ^1H resonances.

Table S2 – Borylation scope varying bases using general procedure B.

Base	Yield	Base	Yield
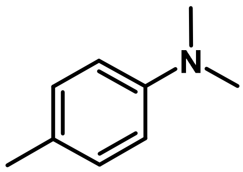	44%	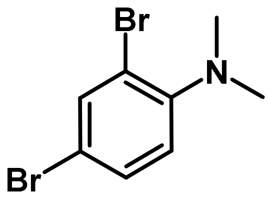	77%
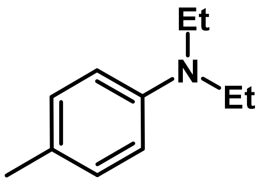	65%	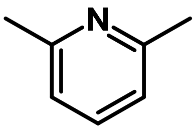	7%
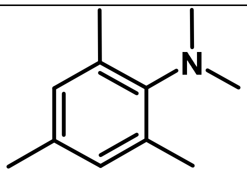	14%	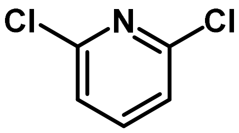	12%
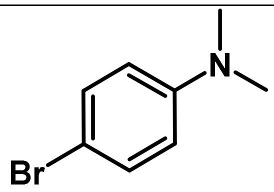	56%		

Varying conditions with *N,N*-dimethyl-2,4-dibromoanline

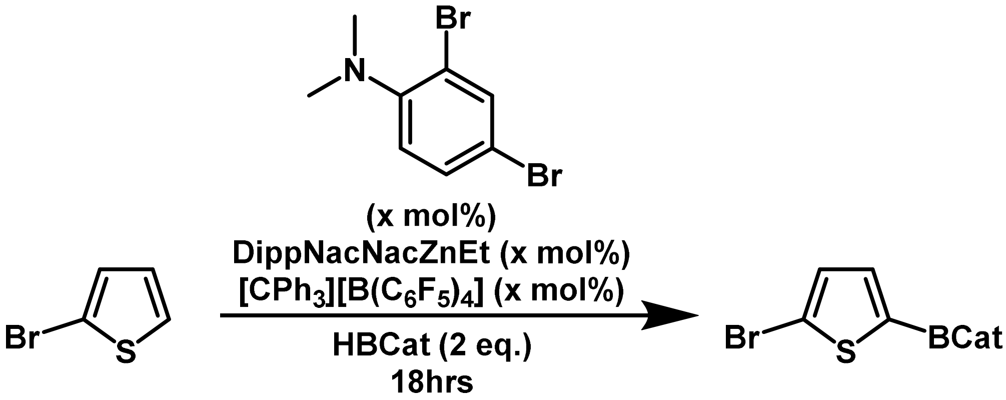

General procedure C: A J Young's NMR tube was charged with equimolar amounts of NacNacZnEt and $[CPh_3][B(C_6F_5)_4]$ before dissolution in PhCl and addition of one equivalent (relative to catalyst) of the base, catecholborane (107 µL, 1.00mmol) and 2-bromothiophene (49 µL,0.50 mmol). The reaction mixture was heated at 80°C (unless otherwise stated) for 18 hours and monitored by ^{1}H and ^{11}B NMR spectroscopy. Addition of dibromomethane (35 µL, 0.50 mmol) as an internal standard was done at the end of the reaction and used to determine *in-situ* yields by integration of diagnostic ^{1}H resonances.

Table S3 – Borylation using general procedure C.

Variation in procedure	Yield
Catalyst loading decreased to 5mol%	39%
Solvent changed to C_6D_6	44%
Temperature raised to 100°C	60%
Reaction concentration doubled	78%

S6. Stoichiometric reactions with NacNacZnEt

Without stabilisation

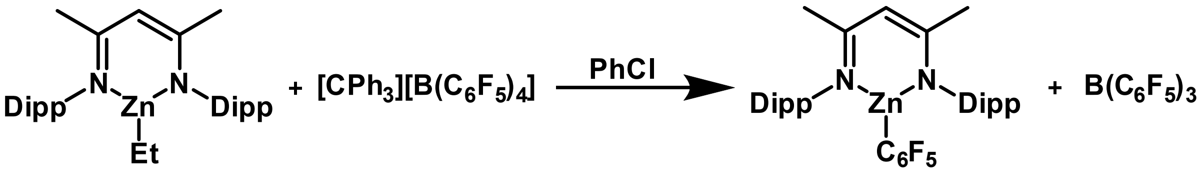

A J Young's NMR tube was charged with NacNacZnEt (26 mg, 0.05 mmol) and $[CPh_3][B(C_6F_5)_4]$ (46 mg, 0.05 mmol) before dissolution in PhCl (1 mL). The reaction was then monitored by ^1H and ^{19}F NMR spectroscopy, which confirmed the formation of the anion decomposition products shown.[4,17]

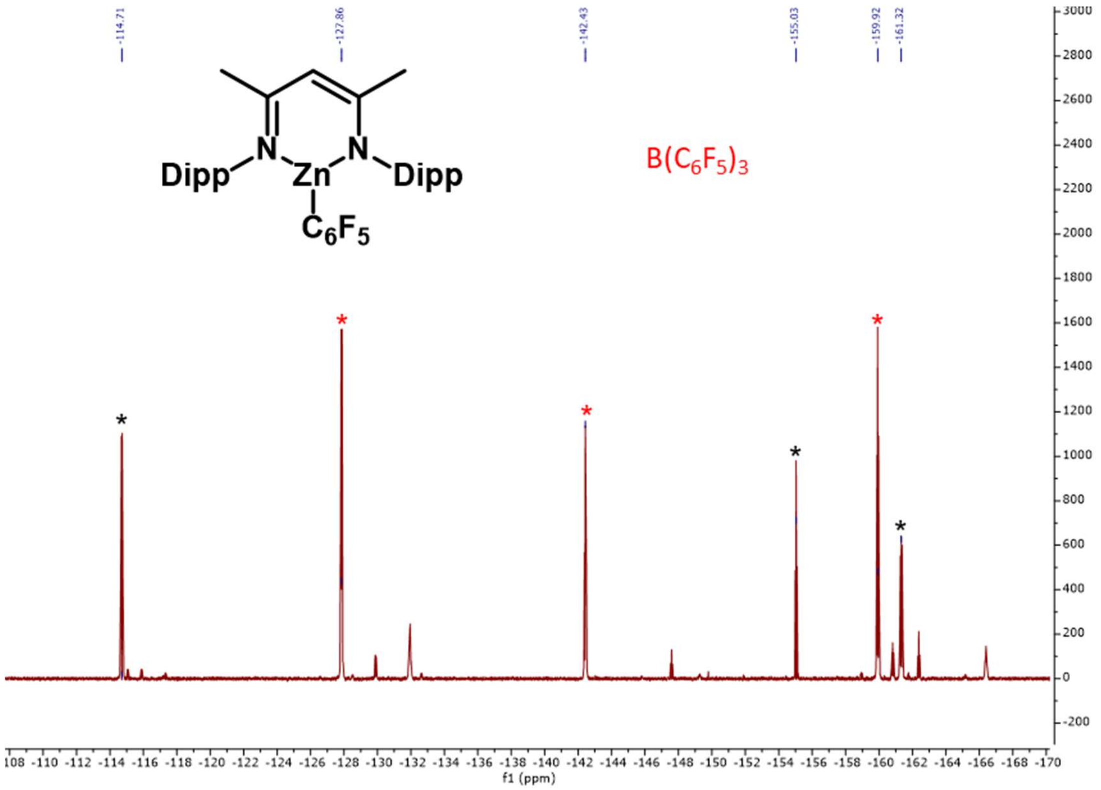

Figure S38: Reaction of NacNacZnEt and $[CPh_3][B(C_6F_5)_4]$ in PhCl after 18 hours at room temperature as observed by ^{19}F NMR spectroscopy.

Attempted stabilisation by borane coordination

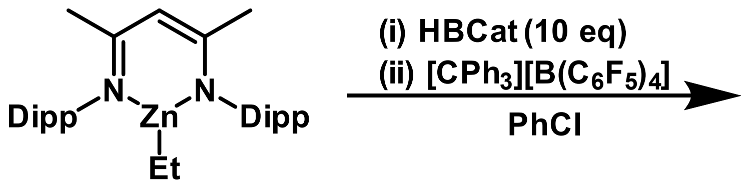

A J Young's NMR tube was charged with NacNacZnEt (26 mg, 0.05 mmol) which was dissolved in PhCl (0.5 mL) before addition of catechol borane (10 eqv., 53 µL, 0.5 mmol). ^{1}H and ^{11}B NMR spectroscopy was conducted on the sample before addition of $[CPh_3][B(C_6F_5)_4]$ (46 mg, 0.05 mmol) dissolved in PhCl (0.5 mL). This revealed that there is minimal metathesis between the Zn-Et and CatBH under these conditions.

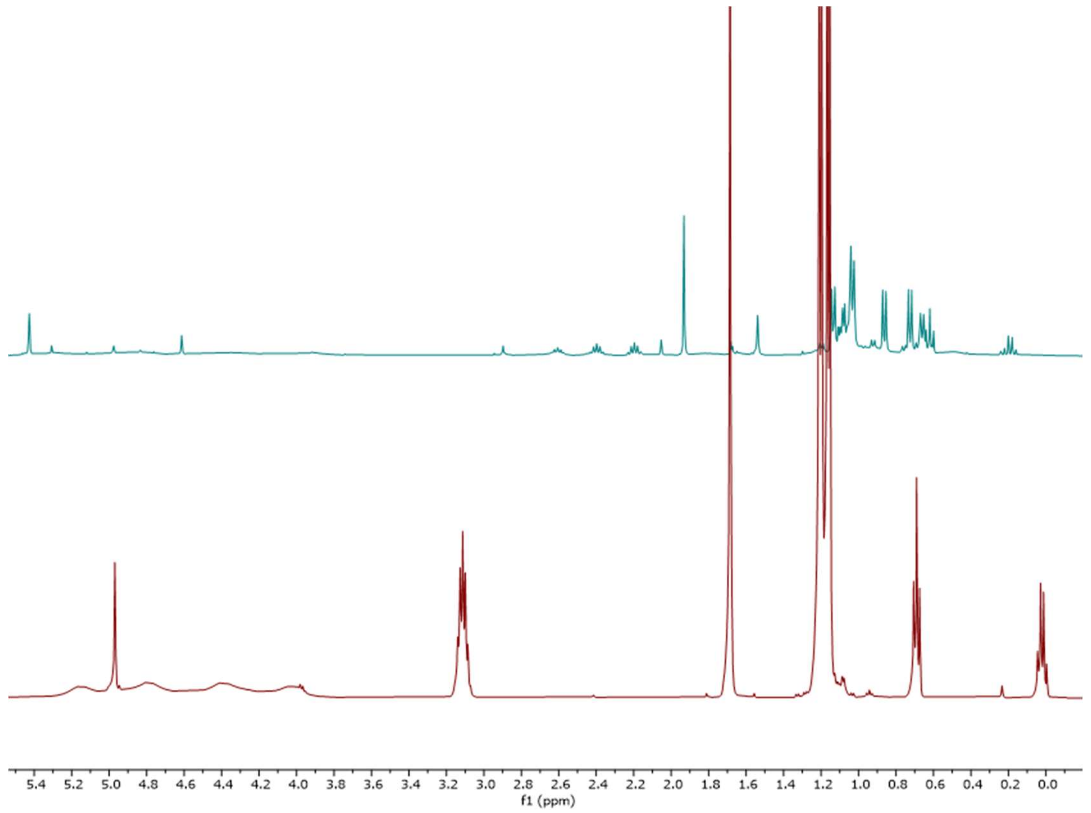

Figure S39: (Red): NacNacZnEt + HBCat (10 eq) in PhCl as observed by ^{1}H NMR spectroscopy. (Teal): Reaction mixture after addition of $[CPh_3][B(C_6F_5)_4]$ in PhCl as observed by ^{1}H NMR spectroscopy.

Attempted stabilisation by exogenous base coordination

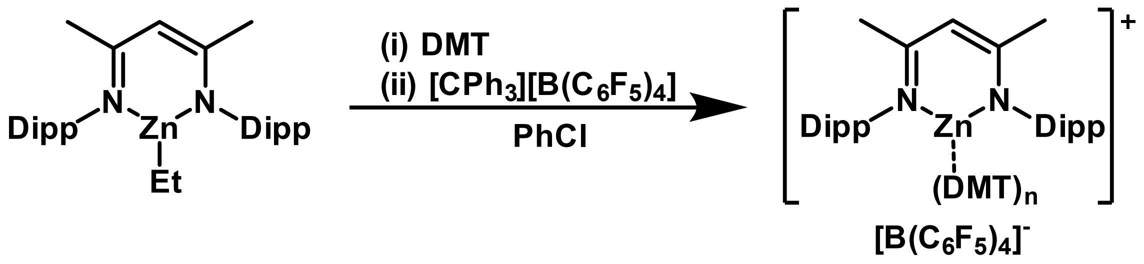

A J Young's NMR tube was charged with NacNacZnEt (26 mg, 0.05 mmol) and dissolved in PhCl (0.5 mL) before addition of *N,N*-dimethyl-4-toluidine (7.2 µL, 0.05 mmol). ^{1}H NMR spectroscopy was conducted on the sample before addition of $[CPh_3][B(C_6F_5)_4]$ (46 mg, 0.05 mmol) dissolved in PhCl (0.5 mL).

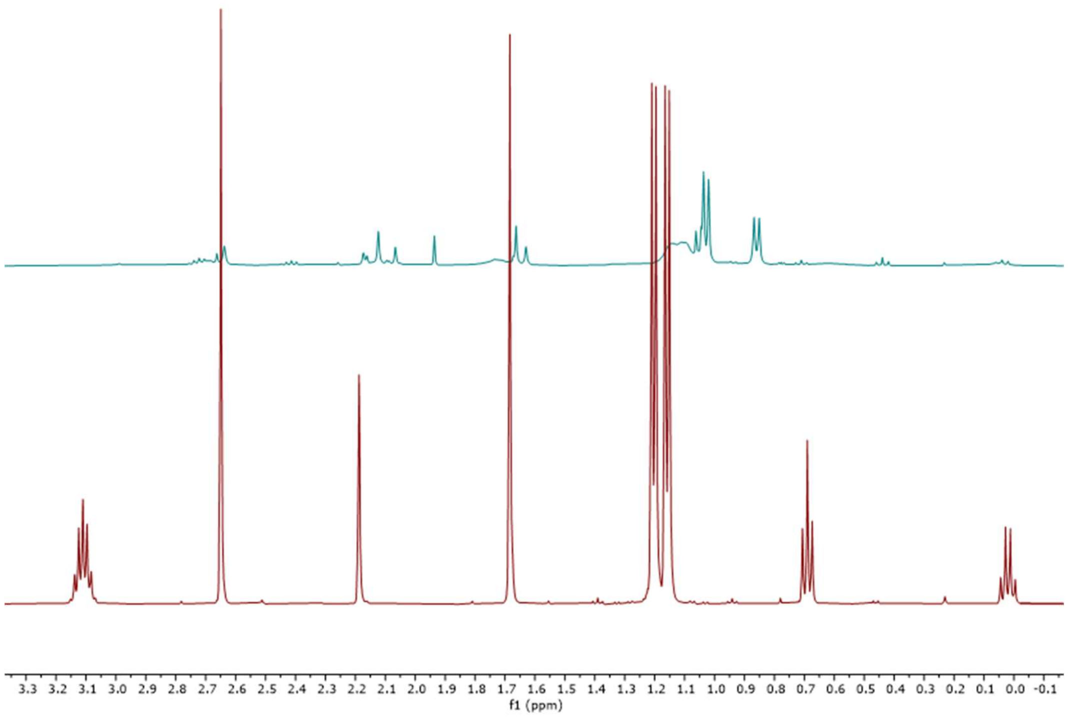

Figure S40: (Red): NacNacZnEt + *N,N*-dimethyl-4-toluidine (1 eq) in PhCl as observed by ^{1}H NMR spectroscopy. (Teal): Reaction mixture after addition of $[CPh_3][B(C_6F_5)_4]$ in PhCl as observed by ^{1}H NMR spectroscopy.

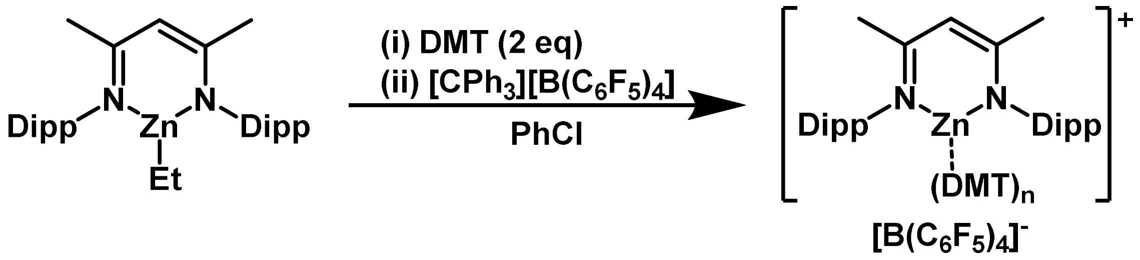

A J Young's NMR tube was charged with NacNacZnEt (26 mg, 0.05 mmol) and dissolved in PhCl (0.5 mL) before addition of *N,N*-dimethyl-4-toluidine (2 equiv., 14.4 µL, 0.1 mmol). ^{1}H NMR spectroscopy was conducted on the sample before addition of $[CPh_3][B(C_6F_5)_4]$ (46 mg, 0.05 mmol) dissolved in PhCl (0.5 mL).

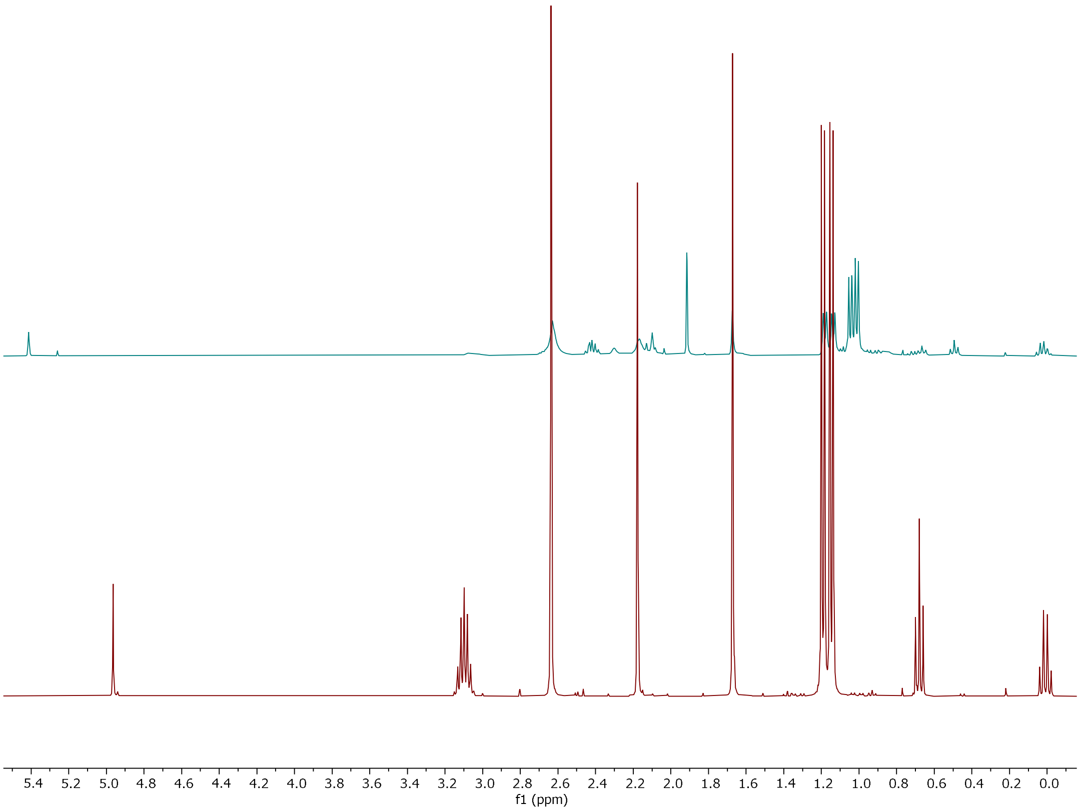

Figure S41: (Red): NacNacZnEt + *N,N*-dimethyl-4-toluidine (2 eq) in PhCl as observed by ^{1}H NMR spectroscopy. (Teal): Reaction mixture after addition of $[CPh_3][B(C_6F_5)_4]$ in PhCl as observed by ^{1}H NMR spectroscopy.

S7. Generation of catalyst 1 from NacNacZnH Vs from NacNacZnEt

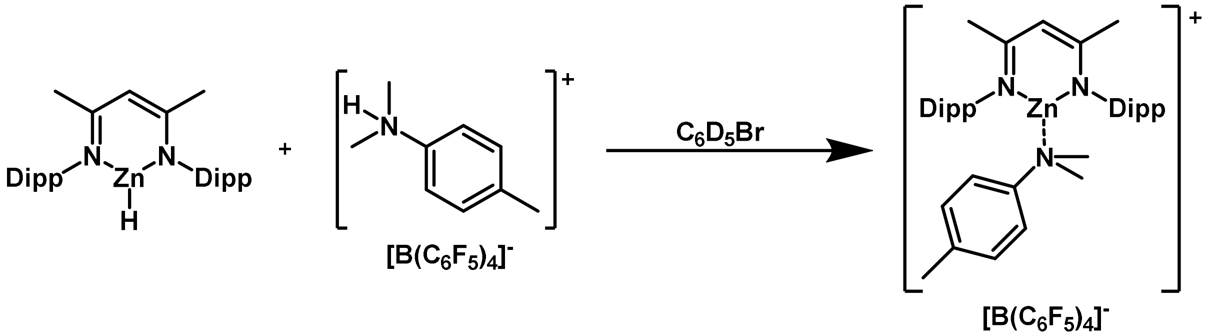

A J Young's NMR tube was charged with NacNacZnH (24 mg, 0.05 mmol) and [DMTH][$B(C_6F_5)_4$] and dissolved in C_6D_5Br (1 mL) causing rapid evolution of gas. The reaction was then monitored by ^1H, $^{13}C\{^1H\}$, ^{11}B and ^{19}F NMR spectroscopy.

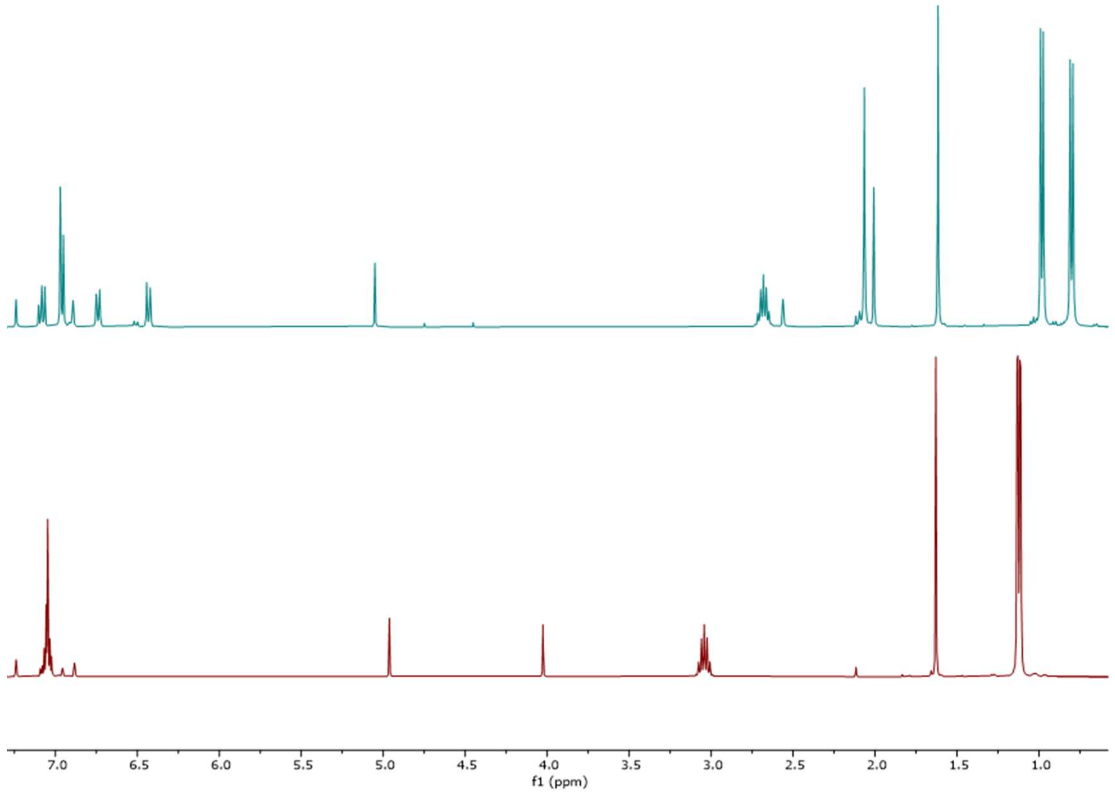

Figure S42: (Red): NacNacZnH in C_6D_5Br as observed by ^1H NMR spectroscopy. (Teal): Reaction between NacNacZnH and [DMTH][$B(C_6F_5)_4$] in C_6D_5Br as observed by ^1H NMR spectroscopy.

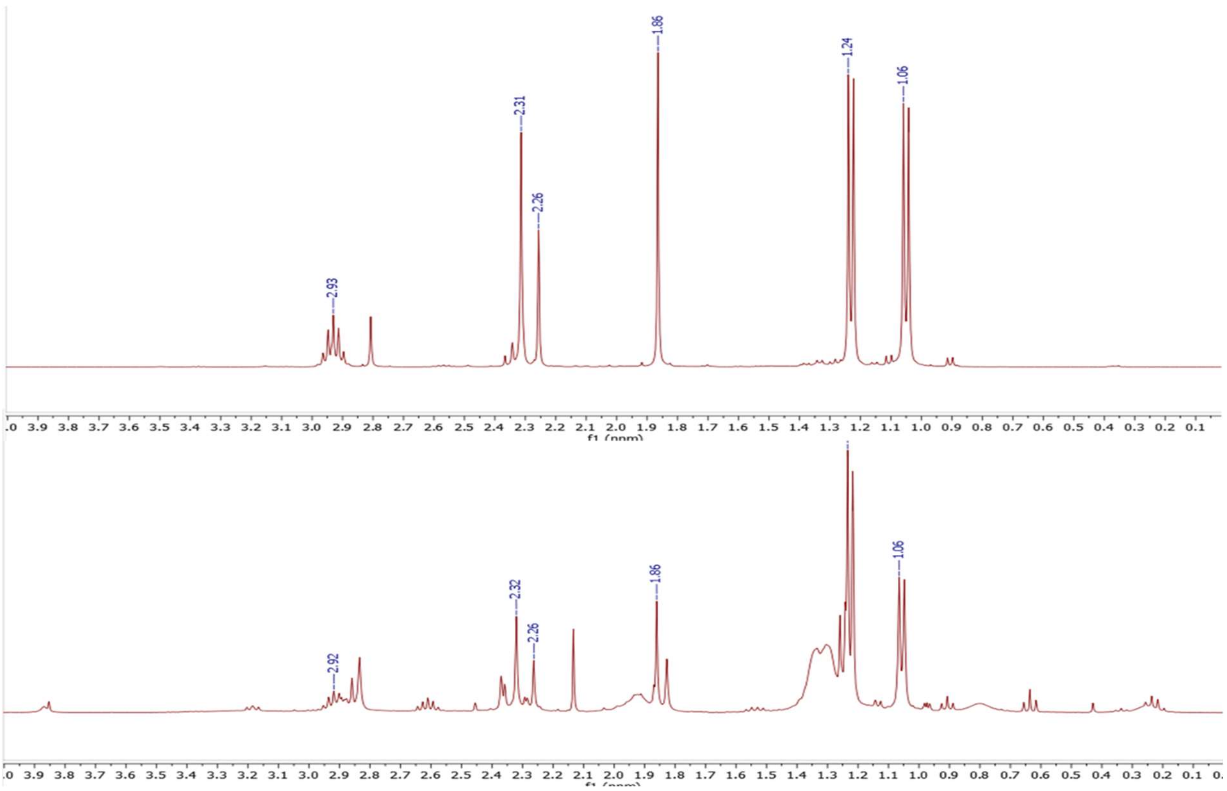

Figure S43: (Top): Reaction between NacNacZnH and [DMTH][$B(C_6F_5)_4$] in PhCl as observed by ^1H NMR spectroscopy. (bottom): Reaction between NacNacZnEt, [CPh_3][$B(C_6F_5)_4$] and DMT (1 eq) in PhCl as observed by ^1H NMR spectroscopy.

S8. Thermal stability tests

NacNacZnH

A J Young's NMR tube was charged with NacNacZnH (24 mg, 0.05 mmol) before dissolution in C_6D_6. The reaction was then heated for 18 hours at 80°C and monitored by ^1H NMR spectroscopy.

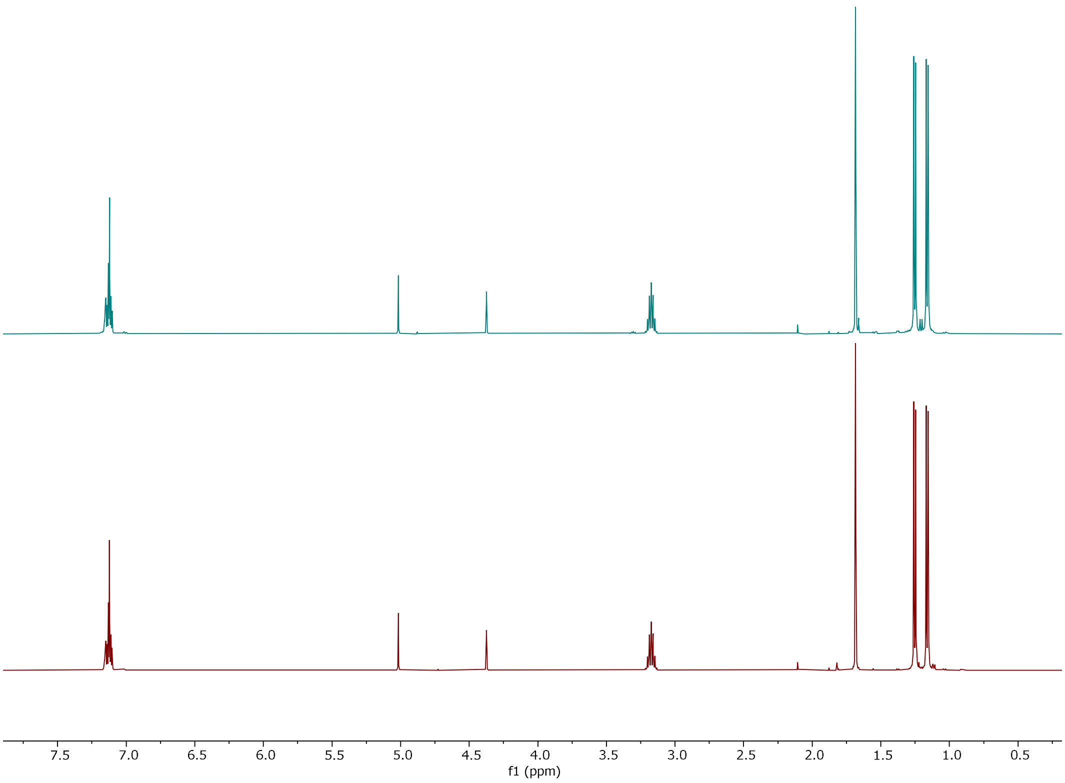

Figure S44: (Red): NacNacZnH in C_6D_6 as observed by ^1H NMR spectroscopy. (Teal): NacNacZnH in C_6D_6 after heating for 18hrs at 80°C as observed by ^1H NMR spectroscopy.

[NacNacZn(DMT)][B(C_6F_5)$_4$]

A J Young's NMR tube was charged with NacNacZnH (24 mg, 0.05 mmol) and [DMTH][B(C_6F_5)$_4$] and dissolved in C_6D_5Br (1 mL) causing rapid evolution of gas. The reaction mixture was then heated for 18 hours at 80°C and monitored by ^{1}H, ^{11}B and ^{19}F NMR spectroscopy.

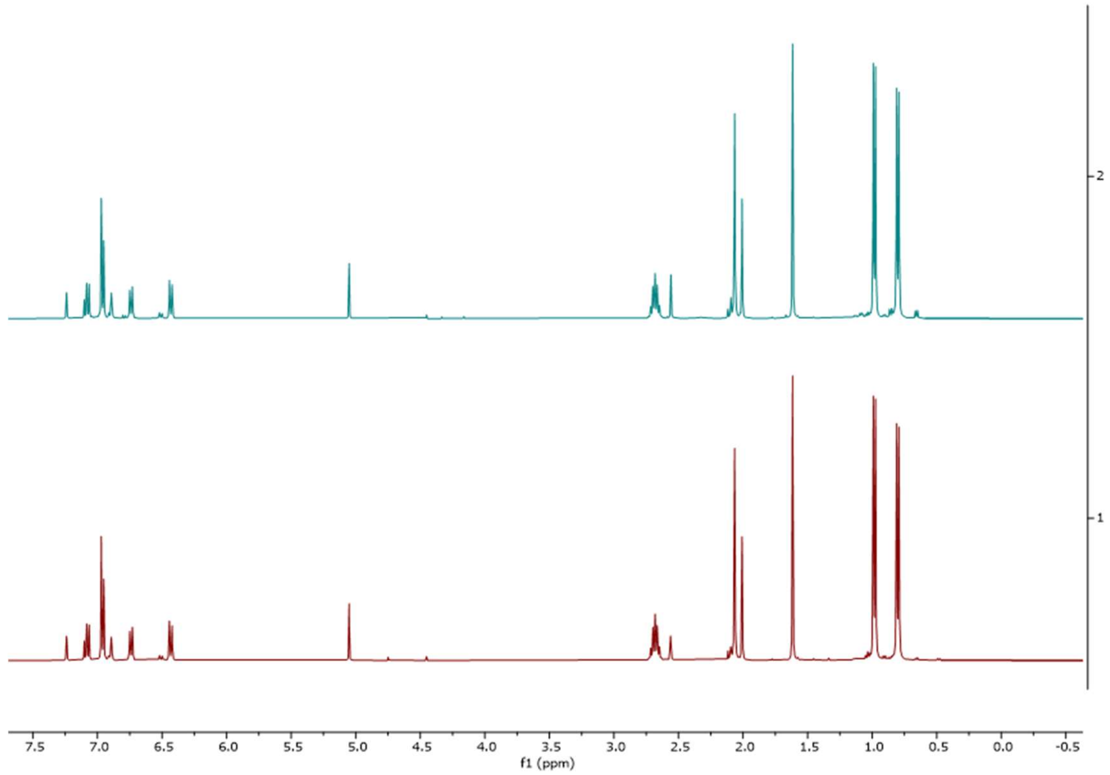

Figure S45: (Red): NacNacZnH + [DMTH][B(C_6F_5)$_4$] in C_6D_5Br as observed by ^{1}H NMR spectroscopy. (Teal): NacNacZnH + [DMTH][B(C_6F_5)$_4$] in C_6D_5Br after heating for 18hrs at 80°C as observed by ^{1}H NMR spectroscopy.

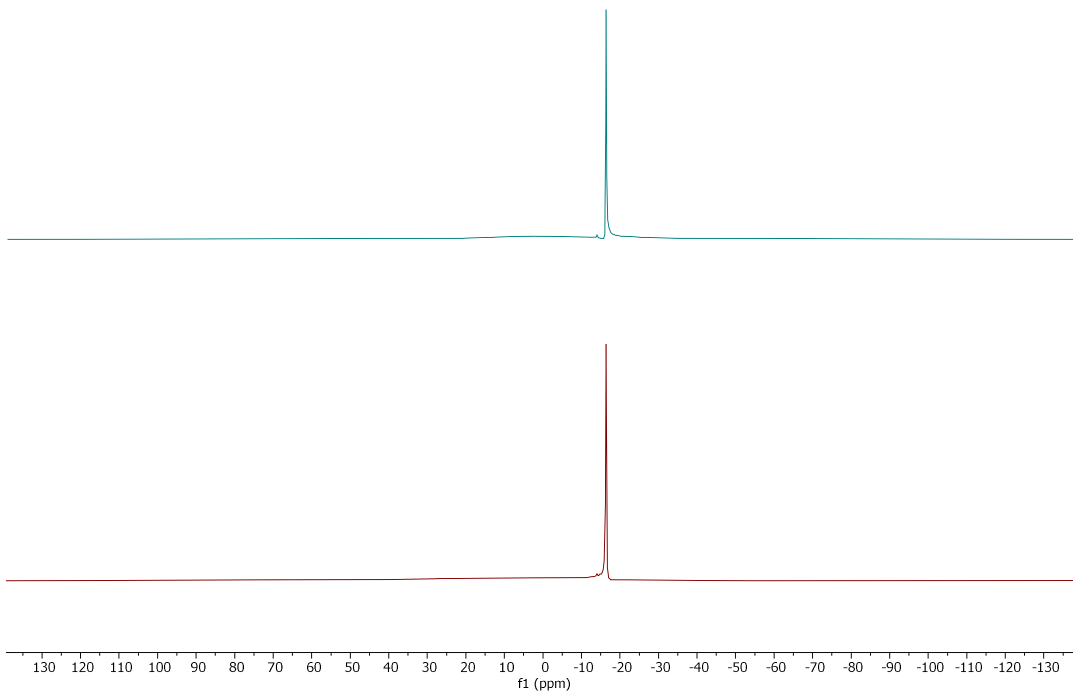

Figure S46: (Red): NacNacZnH + [DMTH][B(C_6F_5)$_4$] in C_6D_5Br as observed by ^{11}B NMR spectroscopy. (Teal): NacNacZnH + [DMTH][B(C_6F_5)$_4$] in C_6D_5Br after heating for 18hrs at 80°C as observed by ^{11}B NMR spectroscopy.

S9. Control reactions

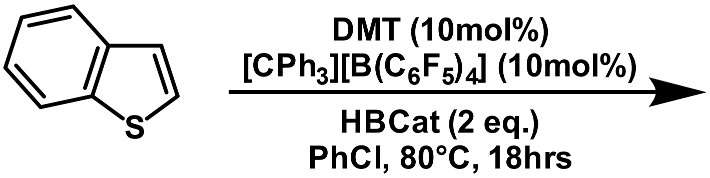

A J Young's NMR tube was charged with $[CPh_3][B(C_6F_5)_4]$ (41 mg, 0.05 mmol) and dissolved in PhCl (1 mL), before addition of *N*,*N*-dimethyl-4-toluidine (7.2 μL, 0.05 mmol), benzothiophene (0.5 mmol) and catecholborane (107 μL, 1.00mmol). The reaction mixture was heated for 18 hours at 80°C, then a further 18 hours at 100°C, then a further 18 hours at 120°C whilst monitoring by ^{1}H and ^{11}B NMR spectroscopy.

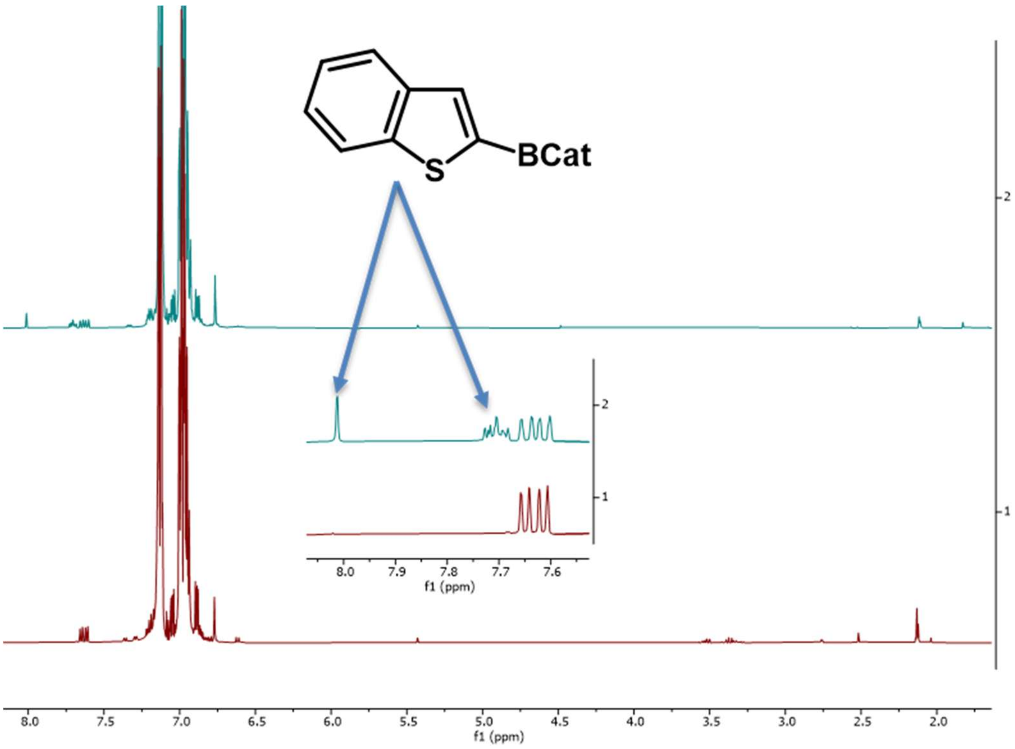

Figure S47: (Red): Control reaction under standard conditions but **without** the presence of NacNacZnEt as observed by ^{1}H NMR spectroscopy after 18 hours at 80°C, 18 hours at 100°C and 18 hours at 120°C. No borylation is observed at any of these temperatures. (Teal): Borylation of benzothiophene using standard catalytic system (i.e. with NacNAcZn) as observed by ^{1}H NMR spectroscopy after 36 hours at 100°C.

[(NacNac)Zn(DMT)][B(C_6F_5)$_4$] + 2-Bromo thiophene

No reaction was observed by NMR spectroscopy on heating an equimolar solution of 2-bromo thiophene and compound **1** at 80°C for 18 h in chlorobenzene.

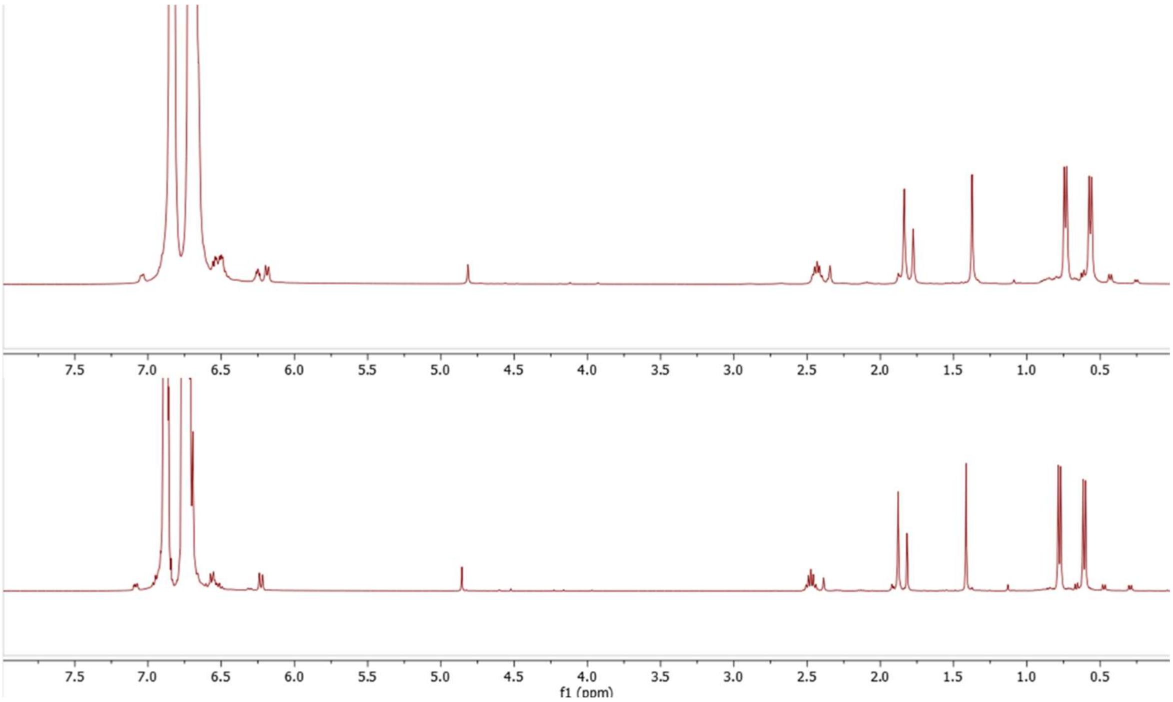

Figure S48: bottom, in-situ formed compound **1** (from [HDMT][(B(C_6F_5)$_4$] and NacNacZnH). Top after addition of 2-bromo-thiophene and heating at 80°C for 18 h.

S10. Computational details: Mechanistic Studies

All geometry optimizations were run with Gaussian16 (Revision A.03)[18] using the BP86 functional.[19,20] Zn centers were described with the Stuttgart RECPs and associated basis sets[21] and 6-31G** basis sets were used for all other atoms.[22,23] Stationary points were characterized with analytical frequency calculations. Transition states (one negative frequency) were characterized via IRC calculations and subsequent geometry optimizations to confirm the adjacent minima. Final free energies were computed using the triple-ζ basis set Def2-TZVP[24,25] and include corrections for dispersion using the D3BJ method[26] and solvation with chlorobenzene as determined by the experimental conditions.[27] Computed geometries and energies are shown below and all geometries are also supplied as a separate XYZ file.

Alternative mechanism

An alternative mechanism for the borylation of *N*-methylindole was considered where the latter is activated by the $[ZnNacNac]^+$ cation to then undergo metathesis with HBcat. The C–H activation step is kinetically accessible but endergonic, while the metathesis is inaccessible due to a high barrier of 26.6 kcal/mol, **TS(L-M)**.

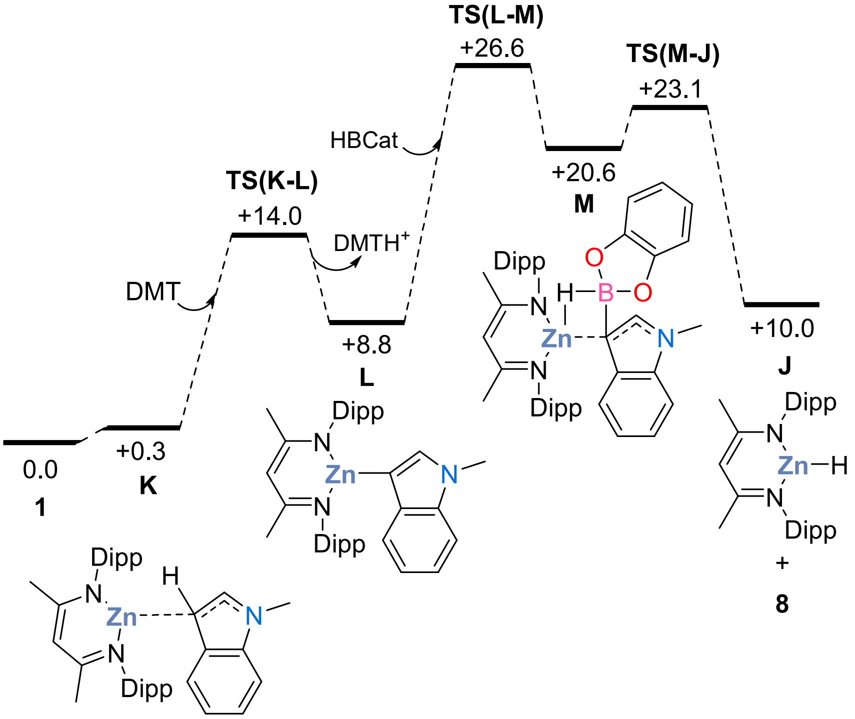

Figure S49: Free energy diagram (kcal/mol) of computed alternative mechanism of C–H activation followed by metathesis.

Computed Structures (Å) and Energies (atomic units)

H_2

```
BP86 energy = -1.17646508218
Enthalpy 0K = -1.166532
Enthalpy 298K = -1.163228
Free energy 298K = -1.178038
Low freq. = 4360.0292 cm-1

H   0.00000  0.00000  0.37512
H   0.00000  0.00000 -0.37512
```

DMT

```
BP86 energy = -405.527073415
Enthalpy 0K = -405.331924
Enthalpy 298K = -405.320122
Free energy 298K = -405.369146
Low freq. = 23.1079 cm-1
Second freq. = 74.9544 cm-1

H   2.49389  2.00877 -0.66887
H   3.86061 -1.06694 -0.03461
H   3.86061  1.06694 -0.03461
C   2.78108 -1.24760  0.07892
C   2.78108  1.24760  0.07892
H   2.60460 -1.67891  1.08807
H   2.60460  1.67891  1.08807
N   2.06377 -0.00000 -0.14810
H   2.49389 -2.00877 -0.66887
C  -0.07465  1.21214 -0.04770
C   0.66649  0.00000 -0.07610
C  -1.47430  1.19931 -0.01789
C  -2.21235  0.00000 -0.00142
C  -0.07465 -1.21214 -0.04770
C  -1.47430 -1.19931 -0.01789
H   0.44100 -2.17543 -0.05357
H  -2.00721 -2.15809 -0.00259
C  -3.72480 -0.00000  0.06886
H  -4.15280  0.89112 -0.42120
H  -4.08831 -0.00001  1.11423
H  -4.15280 -0.89112 -0.42121
H  -2.00721  2.15809 -0.00259
H   0.44100  2.17543 -0.05357
```

$DMTH^+$

```
BP86 energy = -405.904236272
Enthalpy 0K = -405.694617
Enthalpy 298K = -405.682908
Free energy 298K = -405.731509
Low freq. = 30.7497 cm-1
Second freq. = 49.6067 cm-1

H   2.23713  0.24122  2.13113
H   3.79776  0.04233 -1.22883
H   3.79525  0.01922  1.23768
C   2.72400 -0.19268 -1.25568
C   2.72142 -0.21607  1.25816
H   2.57427 -1.27959 -1.28976
H   2.57167 -1.30343  1.27184
N   2.07329  0.34335  0.00572
H   2.24124  0.28067 -2.12091
C  -0.20270  1.34322  0.00500
C   0.58271  0.18558  0.00277
C  -1.59905  1.21120 -0.00243
C  -2.21418 -0.05827 -0.00988
C   0.01343 -1.09441 -0.00770
C  -1.38353 -1.20162 -0.01495
H   0.62710 -2.00088 -0.01395
H  -1.83749 -2.19759 -0.02677
C  -3.71787 -0.19614  0.00900
H  -4.21376  0.71129 -0.36845
H  -4.07787 -0.36615  1.03988
H  -4.05207 -1.05303 -0.59753
H  -2.21832  2.11355 -0.00422
H   0.25259  2.34139  0.00870
H   2.24410  1.36269  0.01541
```

CatBH

```
BP86 energy = -407.025874049
Enthalpy 0K = -406.926244
Enthalpy 298K = -406.919105
Free energy 298K = -406.956554
Low freq. = 217.6737 cm-1
Second freq. = 237.0860 cm-1

C  -0.90636 -1.44324  0.00001
C   0.27389 -0.70242  0.00007
C  -2.10785 -0.70457  0.00001
C  -2.10785  0.70457  0.00001
C   0.27389  0.70242  0.00007
C  -0.90636  1.44324  0.00001
H  -0.89420  2.53609 -0.00006
H  -3.06156 -1.24109 -0.00005
H  -0.89420 -2.53608 -0.00006
H  -3.06156  1.24109 -0.00005
O   1.58786  1.15813 -0.00020
O   1.58786 -1.15813 -0.00020
B   2.36637 -0.00000  0.00057
H   3.55761  0.00000 -0.00050
```

N-MeIndole

BP86 energy = -403.126828338
Enthalpy 0K = -402.973711
Enthalpy 298K = -402.964508
Free energy 298K = -403.006904
Low freq. = 59.8240 cm-1
Second freq. = 130.8868 cm-1

```
C   0.66092 -1.48224 -0.00001
C  -0.15263 -0.33493 -0.00002
C   2.04743 -1.28877 -0.00001
C   2.60699  0.01342  0.00001
C   0.39010  0.99395  0.00001
C   1.79451  1.15145  0.00002
H   2.23842  2.15291  0.00003
H   2.71079 -2.15985 -0.00001
H   0.23145 -2.48946 -0.00001
H   3.69616  0.12668  0.00002
C  -0.72870  1.89969  0.00001
N  -1.53724 -0.22524 -0.00005
C  -1.87267  1.12566 -0.00002
C  -2.46850 -1.34005  0.00005
H  -2.92202  1.42065 -0.00001
H  -0.69411  2.98821  0.00000
H  -2.33372 -1.97139 -0.89653
H  -3.49673 -0.94927 -0.00037
H  -2.33422 -1.97090  0.89705
```

8

BP86 energy = -808.984958471
Enthalpy 0K = -808.750007
Enthalpy 298K = -808.734026
Free energy 298K = -808.793221
Low freq. = 36.6860 cm-1
Second freq. = 43.1837 cm-1

```
C   4.30987 -0.46543  0.00008
C   3.07085  0.19728  0.00029
C   4.28477 -1.86582 -0.00030
C   3.05997 -2.57671 -0.00043
C   1.82117 -0.49718  0.00005
C   1.83035 -1.90843 -0.00026
H   0.88657 -2.46196 -0.00040
H   5.22946 -2.41917 -0.00047
H   5.25695  0.08371  0.00016
H   3.07894 -3.67155 -0.00066
C   0.76501  0.50359  0.00028
N   2.79071  1.56473  0.00089
C   1.42199  1.73407  0.00059
C   3.78840  2.62286 -0.00078
H   1.00007  2.73956  0.00104
H   4.43034  2.55900  0.89530
H   3.27894  3.59739 -0.00029
H   4.42785  2.55847 -0.89861
B  -0.74655  0.30633  0.00025
C  -2.92623  0.73396 -0.00011
C  -2.74616 -0.66124  0.00026
C  -4.19343  1.31321 -0.00041
C  -3.82365 -1.54428  0.00032
C  -5.29224  0.42792 -0.00048
C  -5.11168 -0.96801 -0.00013
O  -1.39179 -0.94860  0.00050
O  -1.69032  1.35707 -0.00004
H  -4.32231  2.39882 -0.00067
H  -3.67136 -2.62681  0.00058
H  -5.98797 -1.62368 -0.00018
H  -6.30660  0.83892 -0.00079
```

[ZnNacNac]$^{+}$

BP86 energy = -1466.34166089
Enthalpy 0K = -1465.723926
Enthalpy 298K = -1465.686349
Free energy 298K = -1465.792213
Low freq. = 17.5892 cm-1
Second freq. = 24.2735 cm-1

```
Zn -0.00002  0.00010 -0.47246
N   1.56740  0.16475  0.55914
N  -1.56744 -0.16463  0.55914
C  -1.28782 -0.22041  1.89054
C  -0.00004  0.00025  2.45039
C   1.28776  0.22077  1.89053
H  -0.00005  0.00036  3.54264
C  -2.41934 -0.52928  2.84891
H  -3.06826 -1.32221  2.44357
H  -3.05757  0.35679  3.00858
H  -2.02706 -0.84695  3.82518
C  -2.88142 -0.36411  0.00003
C  -3.10779 -1.56577 -0.73305
C  -3.87153  0.65038  0.09532
C  -5.11838  0.40304 -0.51484
C  -5.37350 -0.78646 -1.20502
C  -4.36952 -1.75836 -1.32133
C  -3.60229  2.01101  0.74230
H  -5.90122  1.16562 -0.45326
H  -6.35272 -0.95485 -1.66358
H  -4.57591 -2.68025 -1.87230
C  -1.98026 -2.58651 -0.91110
C  -3.39135  3.08476 -0.35322
H  -2.56641  2.81237 -1.03507
H  -3.15376  4.06195  0.10073
H  -4.30018  3.20733 -0.96680
C  -4.71553  2.44587  1.72084
```

```
H  -2.66074  1.94299  1.31489
H  -5.66788  2.63655  1.19781
H  -4.43069  3.38202  2.22942
H  -4.91002  1.68231  2.49300
C  -1.05427 -2.17064 -2.07977
H  -0.16051 -2.81120 -2.15971
H  -0.69199 -1.09951 -2.01002
H  -1.59083 -2.17825 -3.04420
C  -2.45793 -4.03471 -1.14250
H  -1.37781 -2.58913  0.01653
H  -2.97425 -4.15298 -2.11009
H  -3.14856 -4.35326 -0.34532
H  -1.59801 -4.72471 -1.14436
C   2.41929  0.52974  2.84884
H   2.02705  0.84728  3.82517
H   3.06806  1.32280  2.44351
H   3.05769 -0.35624  3.00838
H   1.59111  2.17823 -3.04422
H   1.59873  4.72484 -1.14440
H   0.16096  2.81169 -2.15981
C   2.45852  4.03469 -1.14255
C   1.05452  2.17087 -2.07981
H   3.14922  4.35311 -0.34538
H   0.69188  1.09987 -2.00991
C   1.98060  2.58657 -0.91115
H   1.37814  2.58928  0.01648
H   5.66750 -2.63683  1.19809
H   2.97486  4.15287 -2.11014
C   4.71517 -2.44599  1.72111
H   4.90974 -1.68237  2.49318
C   2.88142  0.36405  0.00004
C   3.60198 -2.01111  0.74252
C   3.10797  1.56565 -0.73309
C   3.87139 -0.65057  0.09540
C   5.11827 -0.40344 -0.51478
C   4.36971  1.75802 -1.32138
C   5.37356  0.78597 -1.20504
H   5.90101 -1.16613 -0.45315
H   4.57624  2.67985 -1.87240
H   2.66044 -1.94291  1.31510
H   6.35279  0.95421 -1.66361
H   4.43022 -3.38205  2.22978
H   4.29973 -3.20768 -0.96646
C   3.39091 -3.08494 -0.35289
H   3.15320 -4.06205  0.10116
H   2.56600 -2.81253 -1.03477
```

1

BP86 energy = -1871.91973189
Enthalpy 0K = -1871.101997
Enthalpy 298K = -1871.052578
Free energy 298K = -1871.185993
Low freq. = 9.7112 cm-1
Second freq. = 15.0865 cm-1

```
Zn  0.29557 -0.22521 -0.16765
N   2.02345 -0.55464  0.65012
N  -0.92067 -1.37671  0.79338
C  -0.40332 -2.14668  1.78546
C   0.96171 -2.16891  2.15299
C   2.07578 -1.44202  1.67524
H   1.19131 -2.85170  2.97395
C  -1.33146 -3.02818  2.60145
H  -2.17730 -3.40391  2.00776
H  -1.75669 -2.45415  3.44381
H  -0.77992 -3.87952  3.02676
C  -2.34342 -1.38123  0.53210
C  -2.83136 -2.11106 -0.59311
C  -3.21307 -0.61109  1.35452
C  -4.58322 -0.58811  1.02511
C  -5.08310 -1.29823 -0.07193
C  -4.21173 -2.05176 -0.87036
C  -2.71722  0.20057  2.55561
H  -5.27036 -0.00353  1.64570
H  -6.15281 -1.27261 -0.30305
H  -4.61380 -2.60834 -1.72183
C  -1.90406 -2.96739 -1.46340
C  -2.73078  1.71759  2.25467
H  -2.11265  1.97015  1.37673
H  -2.34939  2.28432  3.12147
H  -3.75603  2.06952  2.04738
C  -3.52755 -0.09681  3.83836
H  -1.66871 -0.08322  2.75169
H  -4.56799  0.26116  3.75531
H  -3.07423  0.41725  4.70267
H  -3.56724 -1.17582  4.06330
C  -2.36571 -3.08087 -2.93172
H  -1.57728 -3.55438 -3.54029
H  -2.60548 -2.10072 -3.37984
H  -3.26455 -3.71348 -3.02805
C  -1.71032 -4.38472 -0.87053
H  -0.90548 -2.48295 -1.45858
H  -2.68055 -4.90435 -0.78968
H  -1.25402 -4.35638  0.13091
H  -1.05514 -4.98851 -1.52163
C   3.39130 -1.66111  2.39878
H   3.54479 -0.86723  3.15178
H   4.25215 -1.62226  1.71506
H   3.39072 -2.62543  2.92715
H   2.67619  3.93015  1.36090
H   3.24842  2.46715  4.19368
H   1.52135  3.51880  2.65604
C   3.82686  2.18807  3.29690
C   2.03639  3.12538  1.76301
H   4.46011  1.32226  3.55290
```

```
H   1.26066  2.90070  1.00836
C   2.87280  1.87444  2.12177
H   2.16738  1.09792  2.46558
H   5.37257 -2.71825 -1.27291
H   4.49850  3.03224  3.06526
C   4.28812 -2.85341 -1.11869
H   4.11752 -3.02183 -0.04381
C   3.21746  0.14226  0.22408
C   3.50804 -1.62422 -1.64563
C   3.61791  1.32969  0.89849
C   3.92306 -0.33992 -0.91825
C   5.04303  0.39189 -1.36038
C   4.73673  2.02771  0.40049
C   5.44719  1.56709 -0.71384
H   5.60808  0.03707 -2.22781
H   5.06097  2.94537  0.90233
H   2.43754 -1.80109 -1.41899
H   6.31890  2.12051 -1.07692
H   3.97990 -3.76684 -1.65565
H   4.71357 -1.50073 -3.49070
C   3.65548 -1.53365 -3.18048
H   3.21186 -2.42461 -3.65619
H   3.16330 -0.63907 -3.60142
H  -1.13264 -0.43687 -2.87703
H   1.62514  2.31626 -1.15703
H  -4.23595  5.56267 -0.54582
H  -5.33217  4.15452 -0.48198
N   0.03144  1.02273 -1.80090
C  -1.11104  1.95429 -1.56968
C  -0.91050  3.33438 -1.38519
C  -2.42364  1.44400 -1.54670
C  -3.51079  2.30870 -1.37185
C  -2.01342  4.18434 -1.19921
C  -3.33327  3.69770 -1.20348
C  -0.22880  0.16769 -3.01583
C   1.31828  1.75475 -2.04941
C  -4.51644  4.62639 -1.05368
H   0.08750  3.77527 -1.38925
H  -2.61979  0.37234 -1.64546
H   2.10390  1.02059 -2.27114
H   0.63182 -0.50173 -3.16854
H  -1.83234  5.25535 -1.05870
H  -4.51945  1.88345 -1.35793
H  -0.35609  0.81434 -3.90159
H   1.20047  2.44274 -2.90496
H  -4.92867  4.89888 -2.04226
```

D

BP86 energy = -1873.39867393
Enthalpy 0K = -1872.679460
Enthalpy 298K = -1872.633003
Free energy 298K = -1872.764865

Low freq. = 3.4862 cm-1
Second freq. = 12.0956 cm-1

```
Zn  0.16852 -0.18624 -0.00346
N   1.85303 -1.07626  0.00310
N  -1.20290 -1.50194  0.00045
C  -0.76772 -2.79168  0.00457
C   0.59288 -3.18158  0.00745
C   1.79628 -2.43258  0.00749
H   0.74371 -4.26253  0.01080
C  -1.80961 -3.89101  0.00655
H  -2.46628 -3.81116 -0.87633
H  -2.46383 -3.80988  0.89115
H  -1.33861 -4.88339  0.00677
C  -2.61740 -1.19148 -0.00225
C  -3.28226 -1.01081 -1.24792
C  -3.28706 -1.00475  1.23966
C  -4.63652 -0.59968  1.20476
C  -5.30435 -0.39594 -0.00961
C  -4.63165 -0.60574 -1.22051
C  -2.58221 -1.20122  2.58431
H  -5.17297 -0.44619  2.14668
H  -6.35392 -0.08505 -0.01253
H  -5.16465 -0.45710 -2.16504
C  -2.56856 -1.21193 -2.58727
C  -2.24642  0.16139  3.23554
H  -1.61480  0.78849  2.58005
H  -1.70900  0.01745  4.18818
H  -3.16479  0.73554  3.44767
C  -3.39639 -2.08192  3.55790
H  -1.62717 -1.72150  2.39210
H  -4.33381 -1.59150  3.87079
H  -2.81058 -2.27946  4.47129
H  -3.66173 -3.05262  3.10668
C  -2.18652  0.14880 -3.21671
H  -1.64232  0.00247 -4.16512
H  -1.54449  0.74924 -2.54605
H  -3.08588  0.75178 -3.43022
C  -3.39364 -2.05702 -3.58249
H  -1.63060 -1.76088 -2.39049
H  -4.30943 -1.53457 -3.90734
H  -3.69661 -3.02293 -3.14501
H  -2.79863 -2.26436 -4.48770
C   3.10113 -3.20092  0.01299
H   2.92443 -4.28514  0.01554
H   3.70604 -2.93822  0.89739
H   3.71075 -2.94305 -0.86958
H   2.99449  1.64078  3.41823
H   3.62074 -1.33485  4.48764
H   1.86241  0.49596  4.18875
C   4.12179 -0.96074  3.57902
C   2.31645  0.79110  3.22751
H   4.69253 -1.79400  3.13630
```

```
H   1.50422  1.15777  2.57235
C   3.07830 -0.39513  2.59038
H   2.34191 -1.19575  2.39864
H   4.86536 -0.20875 -3.88385
H   4.84594 -0.19223  3.89846
C   4.14390 -0.97906 -3.56267
H   4.71711 -1.80709 -3.11336
C   3.11933 -0.37269  0.00459
C   3.09402 -0.41333 -2.58097
C   3.69569  0.00730  1.24816
C   3.70363 -0.00160 -1.23800
C   4.86592  0.79400 -1.20621
C   4.85817  0.80274  1.21807
C   5.43628  1.20118  0.00635
H   5.33317  1.09723 -2.14864
H   5.31932  1.11275  2.16133
H   2.36025 -1.21619 -2.38845
H   6.33843  1.82087  0.00698
H   3.64796 -1.36054 -4.47102
H   3.00483  1.61820 -3.41876
C   2.32965  0.76651 -3.22687
H   1.88080  0.46486 -4.18854
H   1.51310  1.13272 -2.57681
B   1.20743  2.72802 -0.00994
C  -1.07397  2.73412 -0.00432
C  -0.58247  4.04200 -0.00791
C  -2.42749  2.42545 -0.00041
C  -1.43515  5.14241 -0.00781
C  -2.81494  4.85873 -0.00387
C  -3.29788  3.53630 -0.00024
O   0.81350  4.03796 -0.01137
O   0.05987  1.85718 -0.00549
H  -2.80471  1.39951  0.00248
H  -1.04545  6.16293 -0.01061
H  -4.37585  3.35453  0.00278
H  -3.52664  5.68899 -0.00362
H   2.31756  2.31301 -0.01212
```

TS(D-E1)

```
BP86 energy = -2276.52790095
Enthalpy 0K = -2275.654909
Enthalpy 298K = -2275.598841
Free energy 298K = -2275.752008
Low freq. = -15.0619 cm-1
Second freq. = 12.2188 cm-1

Zn -1.06744  0.47024  0.09857
N  -1.30731  2.35481  0.28000
N  -2.69551 -0.38257  0.59756
C  -3.67836  0.45244  1.03246
C  -3.57383  1.86282  1.07803
C  -2.52753  2.75032  0.72324
H  -4.47354  2.36180  1.44271
C  -4.98564 -0.16162  1.48952
H  -4.80822 -0.96822  2.21962
H  -5.52297 -0.61861  0.64090
H  -5.63877  0.59534  1.94503
C  -2.92006 -1.81010  0.50161
C  -2.45677 -2.65562  1.54860
C  -3.53398 -2.33978 -0.67024
C  -3.64959 -3.74011 -0.77653
C  -3.18601 -4.59068  0.23551
C  -2.60065 -4.04830  1.38588
C  -4.03953 -1.45311 -1.81231
H  -4.11522 -4.17009 -1.66946
H  -3.29093 -5.67535  0.13180
H  -2.25301 -4.71698  2.18050
C  -1.82410 -2.10898  2.83040
C  -3.12388 -1.56534 -3.05417
H  -2.07824 -1.29676 -2.82278
H  -3.47866 -0.89879 -3.85863
H  -3.11720 -2.59586 -3.44941
C  -5.50400 -1.76749 -2.19459
H  -4.00554 -0.40371 -1.47101
H  -5.60536 -2.77694 -2.62823
H  -5.86260 -1.04843 -2.95019
H  -6.17944 -1.71194 -1.32424
C  -0.34396 -2.53707  2.96078
H   0.09933 -2.11365  3.87833
H   0.26506 -2.20322  2.10229
H  -0.25328 -3.63552  3.02350
C  -2.62296 -2.52943  4.08605
H  -1.85228 -1.00576  2.77804
H  -2.60664 -3.62362  4.22763
H  -3.67908 -2.21805  4.02304
H  -2.18665 -2.07062  4.98942
C  -2.79646  4.23495  0.85279
H  -3.84253  4.42618  1.12867
H  -2.57499  4.75591 -0.09352
H  -2.14359  4.68698  1.61886
H   0.52400  2.76246 -3.98751
H  -2.38477  4.08910 -4.03130
H  -1.03261  1.94585 -4.28749
C  -1.65095  4.44384 -3.28787
C  -0.33835  2.27907 -3.49739
H  -2.16506  5.14480 -2.60926
H   0.04130  1.37773 -2.98223
C  -1.04327  3.24767 -2.51957
H  -1.88128  2.70019 -2.05200
H   1.37560  5.12333  3.41197
H  -0.87803  5.01339 -3.83136
C   0.39646  4.61538  3.39086
H  -0.35690  5.36099  3.08526
C  -0.27473  3.31693 -0.04697
C   0.40760  3.38874  2.45076
```

```
C  -0.09894  3.70447 -1.40429
C   0.59116  3.78182  0.98216
C   1.66265  4.61795  0.61157
C   0.98995  4.54305 -1.71484
C   1.86956  4.99110 -0.72246
H   2.34578  4.98486  1.38465
H   1.14679  4.85299 -2.75340
H  -0.57153  2.88805  2.55049
H   2.71042  5.64005 -0.98645
H   0.16702  4.30231  4.42336
H   2.49677  2.82008  2.82759
C   1.48830  2.37668  2.89664
H   1.32699  2.06941  3.94425
H   1.48314  1.46830  2.26841
B   1.84037  0.48407 -0.91112
C   0.87468 -1.54575 -1.30358
C   2.06407 -1.41946 -2.02645
C   0.09015 -2.69120 -1.31409
C   2.53623 -2.45025 -2.83538
C   1.75325 -3.62110 -2.87482
C   0.56359 -3.74021 -2.13113
O   2.64301 -0.17513 -1.80233
O   0.68074 -0.32008 -0.59093
H  -0.82939 -2.78712 -0.73063
H   3.46500 -2.34364 -3.40025
H  -0.01528 -4.66635 -2.18102
H   2.08000 -4.45741 -3.49954
H   1.98451  1.57372 -0.46957
H   4.51961 -4.32119 -0.64170
C   6.03613 -1.45854 -0.51324
C   6.44518 -0.14094 -0.74815
C   5.01615 -1.66077  0.43434
C   5.85642  0.94612 -0.05505
C   3.47174 -2.53329  1.82779
C   4.79008 -4.17773  0.41954
C   4.39550 -0.57597  1.14106
C   4.84178  0.74232  0.88490
C   3.41020 -1.16253  2.01542
N   4.43554 -2.84278  0.87809
H   4.25019 -4.92102  1.02387
H   6.50424 -2.29642 -1.03969
H   7.24162  0.05225 -1.47349
H   6.21271  1.96169 -0.25481
H   2.91824 -3.33436  2.31708
H   2.78470 -0.64812  2.74375
H   4.40616  1.58950  1.42540
H   5.87290 -4.35327  0.53704
```

E1

```
BP86 energy = -2276.53620861
Enthalpy 0K = -2275.661335
Enthalpy 298K = -2275.605987
Free energy 298K = -2275.753962
Low freq. = 8.5542 cm-1
Second freq. = 17.2128 cm-1

Zn -0.88116  0.63166  0.02275
N  -0.42063  2.49627 -0.11924
N  -2.79599  0.58323  0.21567
C  -3.44292  1.77657  0.23473
C  -2.80728  3.03135  0.09107
C  -1.44676  3.38408 -0.07419
H  -3.48942  3.88308  0.11621
C  -4.94708  1.77840  0.42126
H  -5.23132  1.22188  1.32996
H  -5.44960  1.27585 -0.42259
H  -5.33472  2.80364  0.49718
C  -3.54065 -0.65133  0.33963
C  -3.66113 -1.25990  1.62089
C  -4.07914 -1.26152 -0.82831
C  -4.71407 -2.51107 -0.68621
C  -4.82309 -3.13795  0.56147
C  -4.30403 -2.51184  1.70210
C  -3.97198 -0.61953 -2.21442
H  -5.13234 -3.00006 -1.57219
H  -5.32387 -4.10766  0.64737
H  -4.40686 -2.99821  2.67805
C  -3.11235 -0.60629  2.89200
C  -2.94509 -1.36447 -3.09919
H  -1.94529 -1.39523 -2.63311
H  -2.85285 -0.87199 -4.08239
H  -3.25585 -2.40929 -3.27191
C  -5.33984 -0.53572 -2.92915
H  -3.60532  0.41342 -2.08192
H  -5.73564 -1.53628 -3.17379
H  -5.23961  0.01779 -3.87804
H  -6.09685 -0.02109 -2.31336
C  -1.87464 -1.36769  3.42251
H  -1.46990 -0.87511  4.32337
H  -1.07049 -1.41357  2.66654
H  -2.13535 -2.40537  3.69492
C  -4.18701 -0.47956  3.99519
H  -2.78762  0.41642  2.63082
H  -4.52081 -1.46721  4.35671
H  -5.07795  0.06202  3.63585
H  -3.78214  0.06914  4.86250
C  -1.12988  4.86240 -0.19248
H  -2.05056  5.45979 -0.24421
H  -0.52133  5.06518 -1.08861
H  -0.53719  5.20932  0.67139
H   2.50401  1.82069 -3.91400
H   0.10499  3.68850 -4.62859
H   0.90353  1.28610 -4.47447
C   0.68254  3.94045 -3.72283
C   1.49146  1.54012 -3.57571
```

```
H   0.17058  4.77663 -3.21752
H   1.59032  0.63218 -2.95851
C   0.81693  2.70029 -2.80733
H  -0.20464  2.37753 -2.53638
H   2.47302  4.29764  3.58943
H   1.67223  4.30469 -4.04825
C   1.41906  4.39740  3.27792
H   1.30089  5.38619  2.80434
C   0.94237  2.97346 -0.23252
C   0.98150  3.25718  2.33428
C   1.55937  3.03761 -1.51180
C   1.64762  3.31971  0.95553
C   2.99468  3.71091  0.83374
C   2.90797  3.44227 -1.57366
C   3.62426  3.77113 -0.41763
H   3.55600  3.98999  1.73096
H   3.40153  3.50330 -2.54937
H  -0.10862  3.35629  2.18756
H   4.66865  4.09158 -0.49099
H   0.80837  4.38347  4.19629
H   2.30045  1.70632  3.15662
C   1.22104  1.88441  3.00530
H   0.72595  1.83387  3.99032
H   0.82313  1.05786  2.38733
B   1.84258 -0.70193 -0.26751
C   0.06891 -2.20306 -0.49846
C   1.12161 -2.53034 -1.37767
C  -1.01832 -3.04206 -0.27347
C   1.09371 -3.73161 -2.09718
C  -0.00612 -4.58766 -1.89181
C  -1.03717 -4.25623 -0.99408
O   2.08942 -1.56918 -1.40600
O   0.34290 -0.93983  0.08356
H  -1.82429 -2.77752  0.41605
H   1.89766 -3.97710 -2.79637
H  -1.87651 -4.94187 -0.84962
H  -0.05580 -5.53179 -2.44290
H   2.09815  0.46615 -0.39407
H   3.74933 -5.14233 -0.18210
C   2.54628 -2.66227  1.36387
C   4.67056 -2.51227  0.60659
C   5.96022 -2.83267  0.16239
C   6.82010 -1.75437 -0.09092
C   4.21678 -1.18252  0.78643
C   2.78211 -1.25159  1.17267
C   6.39185 -0.41985  0.08480
C   5.08907 -0.11858  0.51151
C   3.67774 -4.83869  0.87487
N   3.61170 -3.38048  0.97120
H   2.76951 -5.27181  1.31509
H   6.28891 -3.86571  0.01829
H   7.84018 -1.95256 -0.43256
H   7.09197  0.39523 -0.12175
H   4.75849  0.91872  0.62120
H   1.64400 -3.16315  1.71522
H   4.56008 -5.20619  1.42166
H   2.38346 -0.56322  1.92901
```

TS(E1-E2)

BP86 energy = -2276.52467908
Enthalpy 0K = -2275.650306
Enthalpy 298K = -2275.595611
Free energy 298K = -2275.741372
Low freq. = -15.4486 cm-1
Second freq. = 11.4759 cm-1

```
Zn -1.05012  0.09800  0.06700
N  -2.14021  1.68643  0.10252
N  -2.27339 -1.37427  0.29330
C  -3.58392 -1.06465  0.46766
C  -4.10039  0.25098  0.45938
C  -3.47362  1.50803  0.28397
H  -5.18030  0.30783  0.60770
C  -4.57081 -2.19522  0.68124
H  -4.24576 -2.85355  1.50388
H  -4.64119 -2.83059 -0.21810
H  -5.57262 -1.80689  0.91102
C  -1.84962 -2.75719  0.26613
C  -1.34255 -3.34945  1.45695
C  -1.89061 -3.47577 -0.96221
C  -1.38112 -4.78910 -0.97515
C  -0.85671 -5.38198  0.18028
C  -0.84503 -4.66629  1.38444
C  -2.44714 -2.86702 -2.25249
H  -1.39853 -5.35795 -1.91060
H  -0.47011 -6.40559  0.14579
H  -0.45279 -5.14143  2.29024
C  -1.32351 -2.60648  2.79539
C  -1.31418 -2.53280 -3.25084
H  -0.57203 -1.84134 -2.81669
H  -1.72819 -2.06828 -4.16217
H  -0.77087 -3.44470 -3.55277
C  -3.50343 -3.77664 -2.92047
H  -2.94705 -1.91791 -1.99128
H  -3.05899 -4.71682 -3.28953
H  -3.95195 -3.26398 -3.78809
H  -4.31734 -4.04503 -2.22568
C   0.12334 -2.27573  3.23214
H   0.12213 -1.71019  4.18070
H   0.64586 -1.67533  2.46592
H   0.70800 -3.19897  3.39074
C  -2.06030 -3.38744  3.90690
H  -1.85584 -1.64904  2.65629
H  -1.55468 -4.34021  4.13989
H  -3.09770 -3.62414  3.61771
```

```
H  -2.09406 -2.79397  4.83650
C  -4.36940  2.73126  0.31906
H  -5.42899  2.44266  0.35479
H  -4.20144  3.37026 -0.56276
H  -4.14834  3.35550  1.20203
H  -0.14121  3.55229 -3.91273
H  -3.16732  2.94424 -4.28761
H  -0.81754  1.98807 -4.41757
C  -2.88815  3.52168 -3.38971
C  -0.53176  2.59026 -3.53795
H  -3.79492  3.64863 -2.77467
H   0.29450  2.07716 -3.01889
C  -1.75565  2.80397 -2.61739
H  -2.13605  1.80835 -2.32470
H  -1.24426  4.99191  3.78292
H  -2.56869  4.52427 -3.72258
C  -2.00799  4.22987  3.55145
H  -2.90239  4.75560  3.17780
C  -1.60630  3.02634 -0.03861
C  -1.49682  3.18519  2.53693
C  -1.38393  3.55786 -1.33812
C  -1.27038  3.75973  1.13486
C  -0.68879  5.03162  0.97629
C  -0.80576  4.83969 -1.43560
C  -0.45529  5.57077 -0.29582
H  -0.42270  5.61501  1.86345
H  -0.63017  5.27030 -2.42717
H  -2.26806  2.39848  2.46111
H  -0.00813  6.56490 -0.39640
H  -2.27369  3.73772  4.50228
H   0.61203  3.23795  3.14579
C  -0.21056  2.50599  3.06341
H  -0.37823  2.06311  4.06079
H   0.12123  1.70259  2.37993
B   1.68413  1.23188 -0.36645
C   1.57852 -1.04654 -0.84360
C   2.33211 -0.36725 -1.81740
C   1.52347 -2.43256 -0.75580
C   3.05679 -1.07909 -2.77653
C   3.00672 -2.48619 -2.71237
C   2.26299 -3.15092 -1.72182
O   2.24341  0.99538 -1.67593
O   0.94086 -0.08214 -0.01059
H   0.93330 -2.94500  0.00866
H   3.64151 -0.55394 -3.53574
H   2.24712 -4.24394 -1.69298
H   3.56661 -3.07149 -3.44805
H   0.97201  2.18865 -0.22305
H   4.21230 -2.32349  2.36692
C   3.02303  0.60549  1.94675
C   4.97288  0.05718  0.95367
C   6.19523 -0.55630  0.64922
C   6.92455 -0.00218 -0.41216
C   4.45258  1.16526  0.24066
C   3.12380  1.49575  0.81897
C   6.43558  1.11170 -1.13083
C   5.19949  1.69928 -0.82132
C   4.21828 -1.36977  2.91867
N   4.05415 -0.25363  1.98698
H   3.39224 -1.36186  3.64250
H   6.57203 -1.41769  1.20789
H   7.88963 -0.43893 -0.68482
H   7.03565  1.52275 -1.94835
H   4.82477  2.55103 -1.39521
H   2.22487  0.53573  2.68692
H   5.17481 -1.26625  3.45495
H   2.88276  2.55029  1.00830
```

E2

BP86 energy = -2276.52692786
Enthalpy 0K = -2275.651826
Enthalpy 298K = -2275.596679
Free energy 298K = -2275.743643
Low freq. = 6.4502 cm-1
Second freq. = 17.0983 cm-1

```
Zn  0.53840  0.70429  0.02228
N   2.46431  0.89342  0.06720
N  -0.14990  2.40801  0.61792
C   0.75791  3.36215  0.94811
C   2.15772  3.20263  0.84336
C   2.95268  2.10238  0.44632
H   2.73143  4.08600  1.13051
C   0.25296  4.69675  1.46039
H  -0.45765  4.55739  2.29163
H  -0.29247  5.24142  0.67106
H   1.08492  5.32671  1.80438
C  -1.56427  2.69952  0.71614
C  -2.27738  2.28640  1.87636
C  -2.22011  3.34591 -0.36968
C  -3.61274  3.53667 -0.27793
C  -4.33716  3.11587  0.84442
C  -3.66990  2.50215  1.91210
C  -1.47214  3.82099 -1.61932
H  -4.13834  4.02736 -1.10366
H  -5.41862  3.27856  0.89332
H  -4.23755  2.19286  2.79645
C  -1.58530  1.62911  3.07343
C  -1.76874  2.91351 -2.83598
H  -1.50324  1.86067 -2.64149
H  -1.20126  3.25647 -3.71816
H  -2.84141  2.93571 -3.09371
C  -1.78353  5.29602 -1.96183
H  -0.38960  3.75180 -1.41374
H  -2.83383  5.43067 -2.27222
```

```
H  -1.14957  5.63297 -2.79928
H  -1.60251  5.96844 -1.10597
C  -2.01790  0.15247  3.23434
H  -1.49284 -0.30975  4.08945
H  -1.79775 -0.43732  2.32699
H  -3.10218  0.07475  3.42620
C  -1.82884  2.41174  4.38392
H  -0.49778  1.63995  2.88067
H  -2.89500  2.40651  4.66832
H  -1.51443  3.46479  4.29344
H  -1.26148  1.95840  5.21481
C   4.45579  2.30554  0.49344
H   4.70314  3.37669  0.48112
H   4.96094  1.80693 -0.34702
H   4.87598  1.87353  1.41901
H   3.30045 -1.10603 -4.38372
H   3.96762  1.89742 -4.13722
H   2.17072  0.22878 -4.67944
C   4.33817  1.25934 -3.31675
C   2.55880 -0.44794 -3.89894
H   4.77187  1.91876 -2.54702
H   1.72532 -1.07521 -3.54538
C   3.19264  0.37922 -2.75840
H   2.41599  1.06138 -2.36505
H   5.36610 -1.62307  3.25269
H   5.15297  0.63253 -3.71876
C   4.92521 -0.61617  3.15595
H   5.69528  0.03965  2.71693
C   3.37761 -0.18928 -0.23598
C   3.63517 -0.63131  2.30520
C   3.68643 -0.48471 -1.59398
C   3.90675 -0.96629  0.83395
C   4.71467 -2.07423  0.51124
C   4.51526 -1.59407 -1.85437
C   5.01585 -2.39191 -0.81915
H   5.13163 -2.68708  1.31765
H   4.77219 -1.83518 -2.89090
H   3.19719  0.38052  2.35067
H   5.65446 -3.25123 -1.04755
H   4.70766 -0.25429  4.17519
H   2.96125 -2.63591  2.89205
C   2.59671 -1.59262  2.92683
H   2.40857 -1.33690  3.98424
H   1.63341 -1.54065  2.38822
B   0.17928 -2.01101 -1.17330
C  -1.76818 -0.71217 -1.19975
C  -1.58913 -1.35313 -2.43750
C  -2.93992 -0.05222 -0.85242
C  -2.58648 -1.30097 -3.41570
C  -3.77606 -0.61887 -3.09051
C  -3.95635 -0.01799 -1.83284
O  -0.38724 -2.00594 -2.51146
O  -0.59193 -0.90177 -0.41169
H  -3.07355  0.42532  0.12122
H  -2.44206 -1.78561 -4.38497
H  -4.89382  0.49476 -1.60166
H  -4.57826 -0.56591 -3.83285
H   1.37105 -1.87089 -1.07870
H  -1.23842 -3.02528  3.60172
C   0.28360 -3.70308  0.86911
C  -1.94176 -4.05390  1.01467
C  -3.22747 -4.30915  1.51210
C  -4.23024 -4.54068  0.56102
C  -1.63354 -3.99437 -0.36879
C  -0.19063 -3.66189 -0.48951
C  -3.94448 -4.51808 -0.82303
C  -2.65527 -4.24303 -1.30080
C  -0.64454 -3.85742  3.19098
N  -0.73465 -3.86209  1.72993
H   0.40587 -3.73859  3.48825
H  -3.44311 -4.34152  2.58380
H  -5.24901 -4.75357  0.89766
H  -4.74871 -4.72115 -1.53632
H  -2.44755 -4.22548 -2.37362
H   1.30195 -3.55952  1.23141
H  -1.03151 -4.80977  3.58687
H   0.42088 -4.18221 -1.24083
```

TS(E2-F1)

```
BP86 energy = -2682.05633641
Enthalpy 0K = -2680.988378
Enthalpy 298K = -2680.921440
Free energy 298K = -2681.095706
Low freq. = -646.9176 cm-1
Second freq. = 6.2078 cm-1

Zn -1.81855  0.20169 -0.23026
N  -2.41581  1.98012 -0.72720
N  -3.45257 -0.84171 -0.18390
C  -4.61689 -0.21542 -0.47891
C  -4.71844  1.14479 -0.85445
C  -3.74055  2.15700 -0.96868
H  -5.73466  1.47428 -1.07845
C  -5.90819 -1.00676 -0.40458
H  -6.01657 -1.49012  0.58056
H  -5.91789 -1.81773 -1.15222
H  -6.77845 -0.35951 -0.58097
C  -3.46603 -2.24460  0.17389
C  -3.47579 -2.60575  1.55055
C  -3.41475 -3.22827 -0.85344
C  -3.34563 -4.58115 -0.46756
C  -3.33404 -4.95727  0.88128
C  -3.40309 -3.97490  1.87718
C  -3.41588 -2.86590 -2.34158
H  -3.29982 -5.35405 -1.24190
```

H	-3.28192	-6.01562	1.15649
H	-3.40787	-4.27430	2.93079
C	-3.55676	-1.56590	2.67156
C	-2.02728	-3.10125	-2.98062
H	-1.23476	-2.53064	-2.46817
H	-2.03824	-2.80470	-4.04379
H	-1.74434	-4.16663	-2.92938
C	-4.50549	-3.62965	-3.12856
H	-3.64049	-1.78824	-2.42758
H	-4.30190	-4.71381	-3.15899
H	-4.54102	-3.27363	-4.17218
H	-5.50820	-3.49369	-2.68856
C	-2.23158	-1.49091	3.46594
H	-2.29527	-0.72118	4.25474
H	-1.37526	-1.24510	2.81432
H	-2.00844	-2.45433	3.95662
C	-4.74748	-1.82842	3.62143
H	-3.72263	-0.57924	2.20351
H	-4.62918	-2.77890	4.16946
H	-5.70388	-1.87681	3.07414
H	-4.82514	-1.02253	4.37127
C	-4.22661	3.53690	-1.36858
H	-5.29284	3.51744	-1.63354
H	-3.65394	3.92676	-2.22553
H	-4.08630	4.25762	-0.54491
H	1.02649	2.56181	-4.00910
H	-1.80747	2.94800	-5.30987
H	-0.01063	1.28509	-4.68395
C	-1.57269	3.53533	-4.40586
C	0.21377	1.85429	-3.76532
H	-2.48678	4.07550	-4.10687
H	0.59281	1.14926	-3.00742
C	-1.04803	2.60706	-3.28423
H	-1.83015	1.85416	-3.08167
H	-2.08800	5.73655	2.47174
H	-0.81868	4.29119	-4.68637
C	-2.82886	4.98877	2.13966
H	-3.43476	5.45567	1.34519
C	-1.51806	3.11302	-0.78723
C	-2.15097	3.68590	1.65689
C	-0.80307	3.38308	-1.98726
C	-1.32689	3.90163	0.38312
C	-0.35722	4.92336	0.34470
C	0.14604	4.42462	-1.97212
C	0.38358	5.18055	-0.81735
H	-0.20140	5.54688	1.23240
H	0.70118	4.65182	-2.88886
H	-2.95168	2.96370	1.42249
H	1.12053	5.99059	-0.83166
H	-3.49296	4.78031	2.99569
H	-0.49441	3.76289	3.09136
C	-1.30379	3.07202	2.79362
H	-1.92566	2.88501	3.68608
H	-0.84114	2.11673	2.49113
B	1.20372	0.50096	-0.17997
C	0.41207	-1.73208	-0.17632
C	1.49147	-1.59074	-1.07126
C	-0.07236	-2.97005	0.22850
C	2.10277	-2.72290	-1.62249
C	1.61068	-3.98756	-1.23646
C	0.54961	-4.11220	-0.32486
O	1.83541	-0.28695	-1.27262
O	-0.01716	-0.44434	0.24434
H	-0.89737	-3.06352	0.93886
H	2.94412	-2.61376	-2.31193
H	0.19154	-5.10296	-0.03206
H	2.07517	-4.88807	-1.65051
H	0.76178	1.56579	-0.55329
H	1.59804	2.38794	5.05900
C	2.03487	1.90370	1.89957
C	2.75276	0.37383	3.38673
C	3.16052	-0.26491	4.56834
C	3.50256	-1.61901	4.46637
C	2.67963	-0.27473	2.12153
C	2.23604	0.72713	1.11251
C	3.43879	-2.29309	3.22522
C	3.03624	-1.63748	2.05306
C	2.30042	2.70658	4.27152
N	2.34956	1.71014	3.20446
H	1.96297	3.66554	3.85505
H	3.20840	0.26179	5.52613
H	3.82275	-2.16145	5.36101
H	3.70687	-3.35322	3.18009
H	2.99455	-2.17754	1.10300
H	1.66112	2.87430	1.56424
H	3.30049	2.83836	4.71694
H	5.65728	1.58213	1.19040
C	5.20251	2.21001	0.41288
H	6.00066	2.73575	-0.14002
H	4.54053	2.95180	0.88968
H	8.46250	-3.09639	-2.23082
N	4.38849	1.37079	-0.50882
C	5.35866	0.00893	-2.38500
H	4.99301	0.71750	-3.13115
C	5.12701	0.21121	-1.01408
C	6.07279	-1.12114	-2.81697
H	3.26879	0.98494	0.30866
H	6.24393	-1.25911	-3.89031
C	5.61540	-0.73379	-0.09018
C	6.57913	-2.06814	-1.90798
C	6.32901	-1.85154	-0.53585
C	7.37963	-3.26306	-2.37629
H	5.41674	-0.61556	0.97916
H	6.69493	-2.57577	0.20040
C	3.76144	2.21765	-1.55248
H	3.12378	2.96505	-1.05484

H   4.52384 2.74235 -2.15601
H   7.21911 -3.46405 -3.44748
H   7.11528 -4.17368 -1.81301
H   3.12919 1.58785 -2.19205

F1

BP86 energy = -2682.07456825
Enthalpy 0K = -2681.001646
Enthalpy 298K = -2680.933796
Free energy 298K = -2681.113375
Low freq. = 4.8443 cm-1
Second freq. = 8.2633 cm-1

```
Zn -2.01399  0.06622 -0.19944
N  -2.75900  1.79196 -0.68578
N  -3.57021 -1.07465  0.00858
C  -4.79257 -0.52266 -0.18774
C  -5.00782  0.81422 -0.59227
C  -4.10685  1.87804 -0.82273
H  -6.05790  1.07753 -0.73277
C  -6.02323 -1.38020  0.03657
H  -6.00098 -1.84267  1.03737
H  -6.06783 -2.20943 -0.68945
H  -6.94131 -0.78399 -0.05928
C  -3.47121 -2.46977  0.37885
C  -3.29613 -2.81130  1.74935
C  -3.49104 -3.46525 -0.63964
C  -3.30538 -4.80708 -0.25405
C  -3.11256 -5.16229  1.08730
C  -3.11282 -4.17045  2.07570
C  -3.67568 -3.12246 -2.12114
H  -3.31135 -5.58814 -1.02155
H  -2.97117 -6.21237  1.36281
H  -2.97215 -4.45337  3.12438
C  -3.29260 -1.76101  2.86286
C  -2.34381 -3.25618 -2.89623
H  -1.55232 -2.61962 -2.46592
H  -2.48233 -2.97259 -3.95401
H  -1.97443 -4.29594 -2.86958
C  -4.77592 -3.97627 -2.79129
H  -3.99040 -2.06647 -2.18842
H  -4.49221 -5.04144 -2.84364
H  -4.94717 -3.63228 -3.82546
H  -5.73508 -3.91542 -2.24943
C  -1.89132 -1.62319  3.50325
H  -1.89867 -0.84653  4.28723
H  -1.12272 -1.34946  2.76039
H  -1.57519 -2.57033  3.97398
C  -4.35931 -2.05934  3.94114
H  -3.55104 -0.78825  2.40761
H  -4.14331 -2.99935  4.47743
H  -5.36852 -2.14887  3.50488
H  -4.38278 -1.24964  4.69034
C  -4.71866  3.20851 -1.22026
H  -5.76922  3.08175 -1.51753
H  -4.16344  3.67592 -2.04828
H  -4.68507  3.92063 -0.37754
H   0.48383  2.57272 -4.21758
H  -2.43136  2.38907 -5.29873
H  -0.35416  1.07745 -4.69010
C  -2.26202  3.06515 -4.44306
C  -0.16905  1.76288 -3.84494
H  -3.24439  3.43781 -4.10842
H   0.37927  1.21141 -3.06345
C  -1.50567  2.32816 -3.31223
H  -2.12976  1.47209 -2.99936
H  -2.59613  5.73570  2.30909
H  -1.68463  3.93197 -4.80909
C  -3.28022  4.90609  2.05999
H  -3.97642  5.27406  1.28738
C  -1.95416  2.98421 -0.84675
C  -2.50842  3.64860  1.59772
C  -1.30887  3.23049 -2.09080
C  -1.79349  3.86623  0.25964
C  -0.94523  4.98017  0.09995
C  -0.48347  4.36690 -2.19681
C  -0.29133  5.23228 -1.11323
H  -0.81323  5.67359  0.93780
H   0.00974  4.58045 -3.15165
H  -3.24659  2.83986  1.45976
H   0.34966  6.11389 -1.21946
H  -3.86623  4.68138  2.96733
H  -0.77945  3.97268  2.91138
C  -1.52867  3.18880  2.70125
H  -2.07222  2.98705  3.64053
H  -0.97883  2.27717  2.41388
B   1.02024  0.66218 -0.14771
C   0.43419 -1.68203 -0.26873
C   1.64525 -1.47958 -0.95785
C  -0.03649 -2.95718  0.02959
C   2.39472 -2.56744 -1.41515
C   1.91282 -3.86540 -1.14389
C   0.72348 -4.05766 -0.42640
O   1.95530 -0.14782 -1.08771
O  -0.13889 -0.44621  0.07600
H  -0.95707 -3.10631  0.59797
H   3.33277 -2.41225 -1.95597
H   0.36982 -5.06926 -0.20928
H   2.48512 -4.73057 -1.49221
H   0.51820  1.58719 -0.75163
H   2.35371  3.73578  4.50281
C   1.81374  2.41318  1.61958
C   3.05272  1.28796  3.12378
C   3.86164  0.91061  4.21073
C   4.17215 -0.44822  4.34130
```

```
C   2.54255  0.34815  2.16580
C   1.74457  1.08822  1.19445
C   3.68036 -1.40098  3.41625
C   2.87116 -1.01926  2.33812
C   2.82653  3.77714  3.50566
N   2.59146  2.54939  2.75980
H   2.39889  4.62320  2.94817
H   4.23025  1.64483  4.93428
H   4.79582 -0.77899  5.17769
H   3.92492 -2.45884  3.55840
H   2.47743 -1.77621  1.65142
H   1.34713  3.29480  1.17522
H   3.90785  3.95520  3.63823
H   4.76499  1.62330  0.47623
C   4.64380  2.02615 -0.53768
H   5.55563  2.55305 -0.85546
H   3.76816  2.68956 -0.56166
H   9.38077 -2.70012 -0.10659
N   4.40306  0.87465 -1.48409
C   6.54163 -0.15628 -2.34323
H   6.56287  0.52633 -3.19677
C   5.50758 -0.12427 -1.39983
C   7.57210 -1.09805 -2.18977
H   3.48199  0.38673 -1.18496
H   8.37705 -1.12798 -2.93144
C   5.49442 -1.00735 -0.30872
C   7.59138 -1.99960 -1.10766
C   6.53393 -1.93578 -0.17417
C   8.71511 -2.99656 -0.93698
H   4.68846 -0.97301  0.43455
H   6.51837 -2.62618  0.67569
C   4.10547  1.37254 -2.86946
H   3.16342  1.93614 -2.82610
H   4.91702  2.02614 -3.21893
H   9.33132 -3.07432 -1.84613
H   8.32809 -4.00101 -0.69708
H   3.99075  0.51075 -3.54027
```

TS(F1-F2)

```
BP86 energy = -2682.06473847
Enthalpy 0K = -2680.992314
Enthalpy 298K = -2680.925277
Free energy 298K = -2681.101071
Low freq. = -31.4696 cm-1
Second freq. = 7.7501 cm-1

Zn  1.17185  0.63883  0.58053
N   0.66530  2.50476  0.93329
N   3.10941  0.65272  0.46005
C   3.71254  1.77453  0.92761
C   3.03438  2.92668  1.39570
C   1.67419  3.31583  1.32849
H   3.69712  3.70307  1.78574
C   5.22983  1.84913  0.93206
H   5.60117  2.19940 -0.04707
H   5.69027  0.86779  1.11793
H   5.57549  2.56482  1.69285
C   3.90580 -0.36751 -0.19194
C   4.35679 -0.15239 -1.52645
C   4.20764 -1.57341  0.50141
C   4.98308 -2.54483 -0.16145
C   5.43646 -2.34893 -1.47014
C   5.11662 -1.16473 -2.14269
C   3.75557 -1.82316  1.94056
H   5.23684 -3.47188  0.36203
H   6.03809 -3.11790 -1.96533
H   5.46647 -1.01859 -3.16996
C   4.01822  1.11064 -2.32392
C   3.24680 -3.26253  2.16446
H   2.49476 -3.54818  1.41066
H   2.78101 -3.34859  3.15985
H   4.06744 -3.99941  2.11945
C   4.87770 -1.49111  2.95332
H   2.91394 -1.13758  2.15053
H   5.76544 -2.12207  2.77259
H   4.53397 -1.67795  3.98551
H   5.19477 -0.43726  2.88922
C   2.92486  0.80912 -3.37458
H   2.66789  1.72219 -3.94046
H   2.00792  0.42120 -2.89947
H   3.27097  0.04607 -4.09301
C   5.25616  1.74070 -3.00116
H   3.60564  1.86105 -1.62760
H   5.66371  1.09157 -3.79504
H   6.06975  1.93021 -2.28072
H   4.98654  2.70161 -3.47199
C   1.37997  4.75712  1.70908
H   2.03980  5.07395  2.53169
H   0.33237  4.90379  2.00791
H   1.58017  5.42826  0.85535
H  -3.39189  1.50640  3.69868
H  -0.97101  2.66446  5.10968
H  -2.08263  0.49448  4.34444
C  -1.20900  3.14809  4.14642
C  -2.42376  1.03824  3.44777
H  -0.40288  3.86471  3.92043
H  -2.59216  0.30030  2.64605
C  -1.37050  2.08576  3.03153
H  -0.40368  1.56412  2.92587
H  -0.79242  5.98856 -2.25638
H  -2.14242  3.72292  4.27791
C   0.13245  5.63467 -1.76887
H   0.25016  6.20633 -0.83360
C  -0.66825  3.01762  0.72144
C   0.09163  4.10937 -1.51367
```

C	-1.68182	2.74933	1.68847
C	-0.96967	3.73579	-0.47382
C	-2.30289	4.13490	-0.69565
C	-2.99488	3.18391	1.41825
C	-3.31212	3.86158	0.23438
H	-2.54784	4.68754	-1.60933
H	-3.77828	3.00401	2.16180
H	1.07811	3.80712	-1.12299
H	-4.33686	4.20217	0.05088
H	0.97380	5.88928	-2.43581
H	-1.11101	3.58953	-3.27898
C	-0.12322	3.35418	-2.84384
H	0.64446	3.64356	-3.58219
H	-0.06123	2.26335	-2.69731
B	-1.05189	-1.35413	0.37158
C	-0.01110	-1.51625	2.50035
C	-0.64195	-2.73803	2.18866
C	0.56606	-1.26650	3.74079
C	-0.69211	-3.76506	3.13566
C	-0.11017	-3.52767	4.40073
C	0.50542	-2.30288	4.70211
O	-1.14648	-2.75523	0.91505
O	-0.09502	-0.66458	1.39797
H	1.04469	-0.30853	3.96524
H	-1.15506	-4.72657	2.89343
H	0.94277	-2.14056	5.69140
H	-0.14213	-4.31715	5.15765
H	-2.16493	-0.76968	0.52190
H	0.06235	0.54512	-4.99821
C	-1.01333	-0.32154	-2.07794
C	0.23936	-1.77398	-3.26070
C	0.89826	-2.48076	-4.28221
C	1.59822	-3.63401	-3.90937
C	0.27197	-2.17847	-1.88932
C	-0.53862	-1.23332	-1.12424
C	1.63546	-4.06594	-2.56102
C	0.98033	-3.35399	-1.55131
C	-0.85022	0.09027	-4.57597
N	-0.55457	-0.62941	-3.34423
H	-1.57552	0.88825	-4.36279
H	0.86497	-2.15172	-5.32549
H	2.12310	-4.21364	-4.67516
H	2.18928	-4.97530	-2.30770
H	1.00278	-3.70309	-0.51522
H	-1.68989	0.52434	-1.94245
H	-1.28453	-0.59357	-5.32525
H	-4.67653	-2.33445	2.12690
C	-4.36021	-3.08800	1.39320
H	-5.15431	-3.83510	1.25542
H	-3.42036	-3.55984	1.70951
H	-9.42488	0.83458	-0.79528
C	-5.17273	-0.18751	-0.34160
C	-8.66426	0.81028	-1.59638
C	-5.27537	-1.58337	-0.36848
C	-6.27378	0.58068	-0.75079
C	-6.45289	-2.21884	-0.78833
C	-7.47289	-0.02209	-1.18006
C	-7.53973	-1.43290	-1.19196
N	-4.10704	-2.39200	0.07796
H	-3.30326	-1.71473	0.25372
H	-4.25212	0.30225	-0.00525
H	-6.19267	1.67235	-0.73426
H	-6.53531	-3.31011	-0.80876
H	-8.45891	-1.92683	-1.52404
H	-8.37675	1.85111	-1.81191
H	-9.15274	0.39484	-2.49319
C	-3.60708	-3.35571	-0.97263
H	-2.66852	-3.79000	-0.60174
H	-4.36633	-4.13162	-1.14164
H	-3.42395	-2.79080	-1.89584

F2

BP86 energy = -2682.07017937
Enthalpy 0K = -2680.997284
Enthalpy 298K = -2680.929911
Free energy 298K = -2681.104904
Low freq. = 9.0542 cm-1
Second freq. = 11.6744 cm-1

Zn	-1.10570	0.32422	-0.43908
N	-0.97423	1.99046	-1.52769
N	-3.00231	-0.16203	-0.65749
C	-3.66927	0.42729	-1.67992
C	-3.14420	1.44548	-2.51133
C	-1.97786	2.23904	-2.39402
H	-3.81940	1.76541	-3.30916
C	-5.09098	-0.01205	-1.99154
H	-5.67396	-0.19514	-1.07657
H	-5.07603	-0.95969	-2.55735
H	-5.61024	0.73903	-2.60392
C	-3.72735	-0.99730	0.27921
C	-4.21272	-0.39949	1.48218
C	-3.93900	-2.37993	0.01710
C	-4.65367	-3.13910	0.96629
C	-5.14831	-2.56484	2.14029
C	-4.92226	-1.20612	2.39152
C	-3.41735	-3.08453	-1.23590
H	-4.82881	-4.20280	0.77146
H	-5.70930	-3.17174	2.85852
H	-5.30774	-0.76165	3.31380
C	-4.01827	1.09008	1.78111
C	-2.37209	-4.15886	-0.86296
H	-1.54802	-3.73164	-0.26963
H	-1.93460	-4.60999	-1.76921
H	-2.83172	-4.96795	-0.26823

C -4.55075 -3.73395 -2.06488
H -2.91498 -2.33062 -1.86775
H -5.03200 -4.55598 -1.50696
H -4.14548 -4.16167 -2.99791
H -5.34227 -3.01518 -2.33347
C -3.85466 1.39339 3.28477
H -3.56089 2.44783 3.42472
H -3.08881 0.75308 3.75210
H -4.80034 1.25285 3.83673
C -5.17204 1.94139 1.19698
H -3.08788 1.40990 1.27168
H -6.13967 1.62583 1.62494
H -5.23976 1.85310 0.10129
H -5.02759 3.00883 1.44041
C -1.92996 3.46406 -3.29442
H -2.18340 3.18106 -4.32941
H -0.94646 3.95335 -3.28727
H -2.68294 4.20385 -2.97154
H 3.77082 1.57924 -3.01252
H 1.66803 1.70562 -5.23094
H 2.93005 0.16508 -3.68151
C 1.54073 2.44465 -4.42085
C 2.87790 0.93948 -2.89706
H 0.59284 2.97717 -4.59679
H 2.93149 0.43363 -1.91900
C 1.56887 1.74271 -3.04015
H 0.73355 1.02427 -2.99900
H -1.32756 6.54638 0.17850
H 2.35897 3.18159 -4.50409
C -1.97254 5.76107 -0.25273
H -1.94330 5.87421 -1.34866
C 0.08040 2.94521 -1.29773
C -1.51604 4.35022 0.19025
C 1.35389 2.76074 -1.91674
C -0.13427 4.01786 -0.38094
C 0.96227 4.83205 -0.03065
C 2.41158 3.61075 -1.53427
C 2.23184 4.62456 -0.58390
H 0.80909 5.65904 0.67210
H 3.39101 3.48766 -2.00898
H -2.23755 3.62044 -0.21440
H 3.06828 5.27363 -0.30385
H -3.00475 5.95552 0.08563
H -0.84629 4.92450 2.20823
C -1.56320 4.23443 1.72883
H -2.56891 4.49148 2.10354
H -1.33143 3.20841 2.06051
B 1.11790 -1.15269 -0.08603
C 0.51376 -2.23526 -2.04056
C 0.97098 -3.19300 -1.10842
C 0.22100 -2.57340 -3.35882
C 1.16476 -4.52503 -1.48420
C 0.88175 -4.87565 -2.82563
C 0.41660 -3.92101 -3.74309
O 1.16463 -2.63559 0.13489
O 0.42003 -0.99435 -1.43354
H -0.14131 -1.82237 -4.06645
H 1.48866 -5.27462 -0.75514
H 0.20386 -4.21804 -4.77433
H 1.02300 -5.91221 -3.14661
H 2.31001 -0.72479 -0.21426
H 0.10826 2.86391 4.04629
C 0.86074 0.98028 1.49455
C -0.07621 0.11398 3.34962
C -0.50959 -0.09917 4.66851
C -1.12515 -1.32784 4.93956
C -0.24028 -0.84369 2.30652
C 0.36385 -0.28751 1.07774
C -1.29791 -2.29855 3.92454
C -0.86265 -2.07173 2.61382
C 0.98780 2.40893 3.56350
N 0.59504 1.22198 2.80760
H 1.43115 3.14036 2.87351
H -0.37221 0.65211 5.45195
H -1.47814 -1.53869 5.95362
H -1.78689 -3.24582 4.17071
H -1.00150 -2.82939 1.83932
H 1.42309 1.71221 0.91062
H 1.72395 2.13978 4.33967
H 4.79014 -2.37385 -1.70388
H 5.36816 -3.73299 -0.67507
C 4.54171 -3.03318 -0.86138
H 3.60650 -3.57533 -1.06830
H 8.60397 2.35799 1.42158
H 6.85574 -2.92832 1.15746
C 6.73757 -1.84828 1.02459
C 7.82489 -0.99701 1.25756
H 8.78095 -1.42925 1.57100
N 4.34270 -2.17433 0.36514
C 5.51302 -1.29162 0.62729
C 7.71275 0.40197 1.09730
C 8.90433 1.30268 1.32997
C 5.36449 0.08965 0.45937
C 6.46702 0.92515 0.69773
H 3.50525 -1.54713 0.14693
H 4.40429 0.51187 0.14443
H 9.61961 1.23151 0.49094
H 6.35330 2.00641 0.56807
H 9.45060 1.01933 2.24487
H 4.70232 -3.76949 1.74777
C 3.94209 -2.99683 1.56814
H 2.95743 -3.43469 1.35259
H 3.87551 -2.32547 2.43473

TS(F2-G1)

BP86 energy = -2682.05558309
Enthalpy 0K = -2680.988621
Enthalpy 298K = -2680.921532
Free energy 298K = -2681.095911
Low freq. = -371.8250 cm-1
Second freq. = 8.9704 cm-1

Zn	-1.18913	0.23961	-0.41744
N	-1.06614	1.86476	-1.52657
N	-2.59238	-0.80587	-1.29354
C	-2.98167	-0.39009	-2.52402
C	-2.48131	0.75732	-3.18683
C	-1.69408	1.83707	-2.72474
H	-2.87665	0.89610	-4.19598
C	-4.03117	-1.18404	-3.28299
H	-4.80116	-1.59231	-2.61174
H	-3.55986	-2.04131	-3.79405
H	-4.51447	-0.56100	-4.04920
C	-3.32075	-1.86581	-0.62323
C	-4.30734	-1.49901	0.34207
C	-3.04752	-3.23422	-0.90390
C	-3.78881	-4.21682	-0.21587
C	-4.76777	-3.87392	0.72049
C	-5.01694	-2.52388	0.99560
C	-1.97581	-3.69238	-1.89444
H	-3.59238	-5.27341	-0.42716
H	-5.33672	-4.65475	1.23550
H	-5.78209	-2.26038	1.73143
C	-4.64029	-0.03467	0.64164
C	-0.84852	-4.44963	-1.15742
H	-0.43685	-3.85278	-0.32865
H	-0.02365	-4.69355	-1.84781
H	-1.22356	-5.39755	-0.73293
C	-2.54903	-4.58666	-3.01930
H	-1.53193	-2.79376	-2.35864
H	-2.92549	-5.54252	-2.61570
H	-1.76197	-4.82750	-3.75419
H	-3.38416	-4.10837	-3.55672
C	-5.09386	0.20672	2.09542
H	-5.16490	1.29014	2.29095
H	-4.39211	-0.23179	2.82339
H	-6.09539	-0.21575	2.28732
C	-5.70169	0.51371	-0.34273
H	-3.71469	0.55610	0.48217
H	-6.63484	-0.07122	-0.26667
H	-5.35895	0.47181	-1.38868
H	-5.94252	1.56540	-0.10796
C	-1.63358	3.05125	-3.63528
H	-1.66183	2.74009	-4.69089
H	-0.73570	3.66039	-3.46086
H	-2.51084	3.70005	-3.46314
H	3.76175	2.88611	-0.94090
H	2.89534	2.37581	-3.83185
H	3.70258	1.27890	-1.69138
C	2.23843	3.05246	-3.25834
C	3.10917	2.01456	-1.12355
H	1.36277	3.28180	-3.88684
H	2.86685	1.55797	-0.15026
C	1.83948	2.40118	-1.91116
H	1.28509	1.47281	-2.12820
H	-3.41661	6.01397	-0.34194
H	2.78867	3.99536	-3.09350
C	-3.58348	5.09669	-0.93270
H	-3.22889	5.29612	-1.95733
C	-0.49681	3.09241	-1.02079
C	-2.86806	3.87772	-0.30352
C	0.90915	3.32004	-1.11512
C	-1.34478	4.02994	-0.35831
C	-0.75367	5.15502	0.25220
C	1.44260	4.46717	-0.49183
C	0.62865	5.37293	0.19996
H	-1.39465	5.88308	0.76188
H	2.51834	4.65877	-0.56331
H	-3.14627	2.98621	-0.89097
H	1.06634	6.25847	0.67237
H	-4.67229	4.92342	-0.97417
H	-3.11310	4.50623	1.79093
C	-3.37256	3.65299	1.13938
H	-4.47099	3.54790	1.15362
H	-2.94092	2.73803	1.58106
B	1.04703	-0.93427	0.67072
C	1.93494	-1.85589	-1.16389
C	1.97766	-2.79239	-0.11105
C	2.53090	-2.10376	-2.39849
C	2.63815	-4.01413	-0.24782
C	3.25498	-4.27101	-1.49198
C	3.19949	-3.33910	-2.54496
O	1.28265	-2.31513	0.98582
O	1.22892	-0.73249	-0.75430
H	2.48296	-1.37323	-3.21033
H	2.65739	-4.74221	0.56768
H	3.68397	-3.57291	-3.49760
H	3.78267	-5.21815	-1.63852
H	2.34864	-0.28179	1.22811
H	-1.72165	3.15676	3.74309
C	0.04905	1.26895	1.77366
C	-1.22582	0.39308	3.41361
C	-1.97659	0.21688	4.58643
C	-2.42971	-1.08167	4.85348
C	-0.92934	-0.66655	2.51081
C	-0.10496	-0.10941	1.41494
C	-2.14477	-2.15326	3.97415
C	-1.40133	-1.96139	2.80321
C	-0.67303	2.87094	3.56645
N	-0.61519	1.56049	2.91783
H	-0.20884	3.61720	2.90657

```
H  -2.19384  1.04547  5.26659
H  -3.01327 -1.26916  5.75976
H  -2.51607 -3.15363  4.21529
H  -1.18663 -2.79457  2.12969
H   0.63274  2.04141  1.26809
H  -0.13944  2.83903  4.53066
H   4.43391 -2.72355  1.75104
H   5.30630 -2.32876  3.27151
C   4.43448 -2.07907  2.64030
H   3.50403 -2.25419  3.20267
H   9.42081 -0.42550 -1.65483
H   6.63576  1.06034  2.55236
C   6.59435  0.54907  1.58732
C   7.59970  0.80422  0.63979
H   8.39667  1.51263  0.89138
N   4.48050 -0.64963  2.22344
C   5.56388 -0.35868  1.28900
C   7.61054  0.16770 -0.61441
C   8.71241  0.42197 -1.61917
C   5.54947 -0.99634  0.03126
C   6.56151 -0.73380 -0.89831
H   3.19085 -0.42055  1.55370
H   4.74242 -1.68844 -0.22970
H   9.29064  1.32488 -1.36667
H   6.53112 -1.23855 -1.87029
H   8.30949  0.54794 -2.63837
H   5.24671  0.09542  4.09144
C   4.39684  0.24138  3.40018
H   3.46349  0.01236  3.93821
H   4.37196  1.28993  3.06761
```

G1

```
BP86 energy = -2275.36914365
Enthalpy 0K = -2274.513261
Enthalpy 298K = -2274.458772
Free energy 298K = -2274.603262
Low freq. = 13.5857 cm-1
Second freq. = 19.5022 cm-1
```

```
Zn -0.23346 -0.13255 -0.34029
N  -1.83441  0.32823 -1.35853
N   0.83571 -1.17831 -1.57371
C   0.40941 -1.24298 -2.85925
C  -0.77462 -0.63583 -3.34388
C  -1.84171  0.00766 -2.67585
H  -0.93806 -0.77246 -4.41543
C   1.23879 -1.99992 -3.88074
H   1.74341 -2.87112 -3.43800
H   2.02552 -1.33959 -4.28655
H   0.61545 -2.33195 -4.72359
C   1.95416 -1.97959 -1.11945
C   1.67375 -3.21433 -0.45939
C   3.29035 -1.52010 -1.28728
C   4.33629 -2.32455 -0.78994
C   4.08133 -3.54279 -0.15246
C   2.75973 -3.97795  0.00885
C   3.63861 -0.19483 -1.96811
H   5.37104 -1.98795 -0.91452
H   4.91037 -4.15534  0.21621
H   2.56757 -4.93233  0.50776
C   0.24343 -3.73820 -0.29592
C   4.28791  0.78542 -0.96518
H   3.66253  0.92388 -0.06853
H   4.44956  1.77261 -1.42971
H   5.26884  0.41126 -0.62396
C   4.57079 -0.38906 -3.18718
H   2.69867  0.26260 -2.32508
H   5.55876 -0.77130 -2.87778
H   4.73599  0.57345 -3.70058
H   4.15860 -1.10214 -3.91974
C   0.02515 -4.53913  1.00377
H  -1.04850 -4.75737  1.13318
H   0.37589 -3.98758  1.89116
H   0.54542 -5.51214  0.97816
C  -0.18712 -4.58384 -1.51918
H  -0.43159 -2.85851 -0.25742
H   0.47935 -5.45571 -1.63771
H  -0.15648 -4.00525 -2.45599
H  -1.21640 -4.96031 -1.38760
C  -3.08265  0.28385 -3.50510
H  -2.81389  0.45155 -4.55890
H  -3.65024  1.14851 -3.13301
H  -3.75986 -0.58862 -3.47393
H  -2.75632  4.96549  0.31186
H  -2.05221  4.68297 -2.62405
H  -1.09394  4.99391 -0.30365
C  -2.76753  3.92768 -2.25563
C  -1.88582  4.32872  0.07858
H  -2.93793  3.20433 -3.06879
H  -1.52275  3.89348  1.02485
C  -2.22365  3.24974 -0.97375
H  -1.28448  2.73271 -1.23725
H  -6.00784 -2.19487 -0.54855
H  -3.72451  4.43937 -2.05331
C  -5.12966 -2.22715 -1.21610
H  -5.38925 -1.66802 -2.13023
C  -3.03498  0.79996 -0.70630
C  -3.86803 -1.65270 -0.53109
C  -3.20322  2.19448 -0.45072
C  -3.99717 -0.15137 -0.25511
C  -5.10839  0.31630  0.47536
C  -4.33918  2.60315  0.27859
C  -5.28249  1.67904  0.74665
H  -5.85763 -0.40221  0.82523
H  -4.49191  3.66927  0.47406
```

```
H  -3.01880 -1.80363 -1.21924
H  -6.15993  2.02299  1.30386
H  -4.96703 -3.28211 -1.49443
H  -4.36621 -2.32415  1.50298
C  -3.55534 -2.44054  0.76199
H  -3.45502 -3.51806  0.54652
H  -2.61307 -2.10260  1.22940
B   1.19339  1.68770  1.26341
C   2.00982  3.51438  0.31014
C   2.85920  3.13630  1.36023
C   2.25501  4.63384 -0.47967
C   4.00191  3.86096  1.68761
C   4.26028  5.00086  0.89931
C   3.40960  5.37716 -0.15878
O   2.36310  1.97660  1.96076
O   0.95395  2.59913  0.22963
H   1.59188  4.91681 -1.30084
H   4.65595  3.55986  2.50946
H   3.64736  6.26803 -0.74711
H   5.14465  5.60658  1.11682
H  -3.27626 -1.05332  3.61156
C  -1.12388  0.79504  2.05368
C  -0.46215 -0.91041  3.37424
C  -0.41770 -1.90252  4.36546
C   0.79943 -2.57830  4.52102
C   0.64408 -0.59440  2.53997
C   0.22767  0.50978  1.64179
C   1.91779 -2.27447  3.70908
C   1.85440 -1.29168  2.71290
C  -2.83699 -0.04591  3.67618
N  -1.51945 -0.04086  3.03482
H  -3.49322  0.66295  3.15291
H  -1.27986 -2.13152  4.99790
H   0.88651 -3.35310  5.28830
H   2.85249 -2.82132  3.86285
H   2.72323 -1.07144  2.08676
H  -1.80043  1.56816  1.68339
H  -2.73747  0.24319  4.73477
```

TS(G1-G2)

```
BP86 energy = -2275.35577895
Enthalpy 0K = -2274.500510
Enthalpy 298K = -2274.446806
Free energy 298K = -2274.588008
Low freq. = -24.5190 cm-1
Second freq. = 18.9356 cm-1

Zn  0.01975 -0.62431  0.25890
N  -1.50730 -1.66355  0.78987
N   1.35773 -0.70982  1.64136
C   1.06397 -1.59900  2.62087
C  -0.07545 -2.45000  2.62650
C  -1.26455 -2.46665  1.86068
H  -0.07444 -3.17122  3.44769
C   2.00137 -1.74543  3.80505
H   2.57726 -0.82720  3.98841
H   2.72328 -2.55895  3.61510
H   1.43853 -2.00938  4.71275
C   2.46373  0.22132  1.74435
C   2.16642  1.57726  2.08080
C   3.79511 -0.19437  1.46090
C   4.81959  0.77098  1.54509
C   4.54951  2.09939  1.88364
C   3.23137  2.49470  2.14364
C   4.17032 -1.62247  1.05775
H   5.85120  0.46786  1.33771
H   5.36342  2.82875  1.94419
H   3.02631  3.53658  2.40443
C   0.74414  2.02955  2.42264
C   4.75549 -1.66024 -0.37296
H   4.06524 -1.22341 -1.11184
H   4.96763 -2.69969 -0.67534
H   5.70355 -1.09792 -0.42673
C   5.17784 -2.25908  2.04527
H   3.25044 -2.23469  1.06110
H   6.14953 -1.73707  2.01206
H   5.36216 -3.31380  1.77869
H   4.82414 -2.22485  3.08831
C   0.47055  3.51430  2.11273
H  -0.60422  3.72879  2.23553
H   0.75911  3.78323  1.08388
H   1.00892  4.18117  2.80820
C   0.40345  1.72239  3.90108
H   0.04150  1.43469  1.79706
H   1.09335  2.26441  4.57076
H   0.48187  0.64844  4.13374
H  -0.62423  2.04752  4.13807
C  -2.34541 -3.43476  2.30497
H  -1.89771 -4.29651  2.82196
H  -2.94262 -3.79327  1.45311
H  -3.04194 -2.94984  3.01072
H  -2.91212 -2.65255 -3.84268
H  -1.88087 -5.08122 -2.25232
H  -1.20143 -3.11580 -3.74465
C  -2.60456 -4.43915 -1.72124
C  -1.98700 -2.51374 -3.25775
H  -2.67800 -4.79857 -0.68139
H  -1.69996 -1.45165 -3.33419
C  -2.16850 -2.95554 -1.78906
H  -1.18792 -2.87708 -1.28528
H  -5.56987 -0.04104  3.14796
H  -3.59106 -4.58144 -2.19494
C  -4.59094 -0.48884  3.39066
H  -4.73813 -1.57943  3.46224
C  -2.83898 -1.50822  0.25175
```

```
C  -3.51634 -0.11346  2.34367
C  -3.14808 -2.06372 -1.02399
C  -3.80000 -0.73674  0.97246
C  -5.06093 -0.52693  0.37820
C  -4.42620 -1.82079 -1.56645
C  -5.37691 -1.05690 -0.87955
H  -5.81352  0.05633  0.91969
H  -4.68339 -2.25104 -2.53980
H  -2.54803 -0.49682  2.70717
H  -6.36637 -0.88734 -1.31604
H  -4.29889 -0.11674  4.38711
H  -4.33519  1.87890  1.90884
C  -3.38705  1.42430  2.24579
H  -3.14514  1.85502  3.23256
H  -2.59443  1.72324  1.53808
B   0.68887  0.74933 -2.21958
C   1.55559 -1.34625 -2.45615
C   2.25579 -0.44145 -3.26467
C   1.82205 -2.70943 -2.43071
C   3.28398 -0.86540 -4.10332
C   3.57503 -2.24505 -4.09172
C   2.86325 -3.14616 -3.27759
O   1.74976  0.84547 -3.10936
O   0.56098 -0.61799 -1.75948
H   1.25977 -3.40274 -1.80010
H   3.82450 -0.16075 -4.73968
H   3.11679 -4.20962 -3.30066
H   4.37268 -2.62278 -4.73756
H  -3.94346  3.53903 -0.19003
C  -1.64836  1.57994 -1.46385
C  -1.39711  3.82127 -1.42926
C  -1.61245  5.20639 -1.33696
C  -0.50634  6.03572 -1.55604
C  -0.12197  3.25776 -1.73098
C  -0.28529  1.81132 -1.74968
C   0.76957  5.49921 -1.85774
C   0.97561  4.11852 -1.94587
C  -3.74441  2.93176 -1.08775
N  -2.30536  2.76720 -1.27577
H  -4.20577  1.94253 -0.95704
H  -2.59721  5.62467 -1.10931
H  -0.63145  7.12099 -1.49620
H   1.60769  6.18148 -2.02877
H   1.96314  3.71305 -2.18587
H  -2.21464  0.64525 -1.46951
H  -4.18968  3.43073 -1.96499
```

G2

```
BP86 energy = -2275.35622159
Enthalpy 0K = -2274.501156
Enthalpy 298K = -2274.446537
Free energy 298K = -2274.592054
Low freq. = 4.8837 cm-1
Second freq. = 17.4347 cm-1

Zn -0.41924 -0.75057 -0.01498
N   0.64319 -2.33552 -0.14481
N  -1.96010 -0.92674 -1.14820
C  -2.06147 -2.13590 -1.75131
C  -1.21127 -3.24256 -1.47782
C   0.02318 -3.35677 -0.79814
H  -1.52899 -4.16680 -1.96751
C  -3.14249 -2.37375 -2.78822
H  -3.48442 -1.43382 -3.24466
H  -4.01865 -2.85847 -2.32362
H  -2.77399 -3.04764 -3.57632
C  -2.77775  0.21036 -1.51264
C  -2.14568  1.27974 -2.21960
C  -4.14103  0.29568 -1.11194
C  -4.85849  1.45855 -1.46115
C  -4.26038  2.50830 -2.16247
C  -2.91221  2.41714 -2.53140
C  -4.85953 -0.78670 -0.30208
H  -5.91035  1.53902 -1.16781
H  -4.84061  3.39977 -2.42030
H  -2.44944  3.24415 -3.07646
C  -0.69116  1.18341 -2.68864
C  -5.25663 -0.25763  1.09577
H  -4.38588  0.12141  1.65282
H  -5.72461 -1.06060  1.69131
H  -5.98832  0.56450  1.01416
C  -6.11513 -1.32362 -1.03038
H  -4.16078 -1.62908 -0.15262
H  -6.88947 -0.54222 -1.11610
H  -6.55689 -2.16124 -0.46418
H  -5.89262 -1.67710 -2.05009
C   0.00200  2.54468 -2.88249
H   1.07574  2.39235 -3.08045
H  -0.08994  3.19038 -1.99474
H  -0.40954  3.08750 -3.75071
C  -0.57834  0.33445 -3.97730
H  -0.10872  0.64705 -1.89717
H  -1.14985  0.81165 -4.79189
H  -0.97252 -0.68515 -3.84151
H   0.47354  0.25536 -4.30053
C   0.70442 -4.71150 -0.84441
H  -0.04136 -5.50994 -0.97252
H   1.28546 -4.90177  0.07067
H   1.40606 -4.77221 -1.69415
H   2.37995 -1.88703  4.46333
H   0.41364 -4.21820  4.02014
H   0.61693 -1.72686  4.58979
C   1.20819 -4.11801  3.26089
C   1.46503 -1.66805  3.88707
H   1.00287 -4.84366  2.45639
```

```
H   1.53953 -0.62938  3.52445
C   1.27033 -2.66710  2.72571
H   0.29443 -2.45280  2.25125
H   4.60375 -3.14608 -3.03798
H   2.16367 -4.40347  3.73354
C   3.51096 -3.29757 -3.06177
H   3.31409 -4.31931 -2.69643
C   2.02754 -2.43046  0.26030
C   2.76854 -2.22631 -2.22907
C   2.34492 -2.52753  1.64631
C   3.05474 -2.35395 -0.72855
C   4.39515 -2.37395 -0.29251
C   3.70332 -2.54392  2.02076
C   4.72383 -2.46660  1.06613
H   5.19692 -2.32768 -1.03746
H   3.96372 -2.63143  3.08047
H   1.68668 -2.36819 -2.39118
H   5.77263 -2.49086  1.37877
H   3.19645 -3.24521 -4.11770
H   4.20627 -0.61649 -2.64219
C   3.12518 -0.81386 -2.74849
H   2.87613 -0.72188 -3.81963
H   2.58135 -0.02503 -2.20031
B   0.21590  1.89064  1.50660
C  -1.33421  0.56757  2.55971
C  -1.47422  1.89670  2.97370
C  -2.07114 -0.48016  3.09686
C  -2.39527  2.25394  3.95599
C  -3.16087  1.20897  4.51225
C  -3.00221 -0.12644  4.09823
O  -0.56523  2.71080  2.31619
O  -0.30255  0.52325  1.58263
H  -1.93727 -1.51747  2.77761
H  -2.49979  3.29108  4.28316
H  -3.60855 -0.91114  4.55857
H  -3.89054  1.44406  5.29220
H   5.12177  1.81797 -1.49814
C   2.53934  1.42716  0.43782
C   3.21468  3.49651 -0.15538
C   3.95057  4.59951 -0.61936
C   3.32862  5.85167 -0.54994
C   1.89273  3.61389  0.36684
C   1.45822  2.27362  0.74243
C   2.01755  5.99390 -0.03409
C   1.29146  4.88915  0.42377
C   4.89759  1.62511 -0.43576
N   3.57950  2.14692 -0.08984
H   4.91182  0.54065 -0.25624
H   4.96477  4.49232 -1.01528
H   3.86777  6.73742 -0.89899
H   1.56716  6.99024  0.00928
H   0.28227  5.01119  0.82875
H   2.66497  0.35843  0.62496
H   5.67276  2.10468  0.18532
```

TS(G2-H)

```
BP86 energy = -2682.37853157
Enthalpy 0K = -2681.422924
Enthalpy 298K = -2681.360862
Free energy 298K = -2681.523119
Low freq. = -21.9251 cm-1
Second freq. = 8.4467 cm-1

Zn  0.66103 -0.03985 -0.56111
N  -0.00415  0.94902 -2.06928
N   1.98691 -1.30544 -1.19716
C   2.01251 -1.45209 -2.54197
C   1.18981 -0.72213 -3.44042
C   0.36071  0.41393 -3.26769
H   1.32017 -1.01371 -4.48501
C   3.00290 -2.41418 -3.16924
H   3.94769 -1.88843 -3.39518
H   2.61124 -2.80692 -4.11971
H   3.24819 -3.25066 -2.49935
C   2.97833 -1.94429 -0.35437
C   2.63410 -3.12865  0.35928
C   4.26352 -1.34331 -0.21379
C   5.18281 -1.94403  0.66952
C   4.86124 -3.10555  1.38022
C   3.60054 -3.69039  1.21658
C   4.68530 -0.08107 -0.97260
H   6.17391 -1.49474  0.79295
H   5.59450 -3.55874  2.05476
H   3.35806 -4.60604  1.76508
C   1.28581 -3.83094  0.18770
C   4.84592  1.12106 -0.01413
H   3.90682  1.34619  0.52071
H   5.14217  2.02674 -0.57097
H   5.62313  0.92585  0.74488
C   5.98715 -0.29758 -1.77923
H   3.88769  0.17502 -1.69177
H   6.85226 -0.46586 -1.11545
H   6.21072  0.59233 -2.39162
H   5.91545 -1.16794 -2.45263
C   0.65280 -4.25173  1.53196
H  -0.37954 -4.60534  1.37169
H   0.62761 -3.41924  2.25461
H   1.20890 -5.07940  2.00455
C   1.40931 -5.06069 -0.74458
H   0.59704 -3.11630 -0.29677
H   2.11210 -5.80041 -0.32394
H   1.77395 -4.78660 -1.74822
H   0.43016 -5.55634 -0.86258
C  -0.16117  1.05849 -4.53936
H   0.40713  0.71625 -5.41552
```

H	-0.11362	2.15645	-4.48698
H	-1.22122	0.79227	-4.69152
H	2.30092	5.40707	-1.41379
H	3.49541	3.21559	-2.93001
H	3.36239	4.22734	-0.62684
C	2.39982	3.23636	-3.06243
C	2.30814	4.45370	-0.85763
H	2.11721	2.34138	-3.63801
H	1.77197	4.60993	0.09169
C	1.69470	3.30558	-1.68510
H	1.91956	2.35860	-1.15221
H	-4.52684	2.14867	-3.62044
H	2.15162	4.12557	-3.66747
C	-3.82040	1.30298	-3.67618
H	-3.18552	1.46824	-4.56233
C	-0.65117	2.24994	-2.02152
C	-2.99898	1.17279	-2.37041
C	0.17117	3.40154	-1.82313
C	-2.06280	2.36588	-2.16069
C	-2.63004	3.65647	-2.10577
C	-0.45519	4.66149	-1.78209
C	-1.84172	4.79504	-1.92350
H	-3.71426	3.76460	-2.21523
H	0.15398	5.55727	-1.63468
H	-2.38017	0.25999	-2.44006
H	-2.30416	5.78644	-1.88936
H	-4.41758	0.39078	-3.84653
H	-4.60318	1.88659	-1.04877
C	-3.96168	0.99711	-1.17377
H	-4.62256	0.12801	-1.32976
H	-3.41786	0.83172	-0.22999
B	-1.43028	-1.27228	1.44944
C	0.49153	-0.60833	2.50507
C	-0.50413	-0.88080	3.44875
C	1.80304	-0.30571	2.84960
C	-0.24395	-0.81403	4.81632
C	1.07136	-0.47571	5.19157
C	2.07457	-0.23716	4.23371
O	-1.68012	-1.25575	2.81995
O	-0.07898	-0.76718	1.21503
H	-1.02347	-1.02893	5.55099
H	3.09071	-0.00259	4.56192
H	1.32084	-0.41404	6.25456
H	-3.35372	-4.35478	-2.90552
C	-2.05973	-2.16242	-0.93859
C	-4.19509	-2.81692	-0.63217
C	-5.48996	-3.33598	-0.80005
C	-6.34370	-3.27888	0.30762
C	-3.73897	-2.25184	0.59572
C	-2.35709	-1.82591	0.39161
C	-5.91476	-2.72287	1.53727
C	-4.62358	-2.20792	1.69419
C	-3.18161	-3.26525	-2.91117
N	-3.14013	-2.74591	-1.54885
H	-2.22198	-3.05748	-3.40550
H	-5.82092	-3.76917	-1.74863
H	-7.36052	-3.67345	0.22112
H	-6.61024	-2.69823	2.38178
H	-4.30116	-1.78427	2.64952
H	-1.12931	-2.05898	-1.50068
H	-3.98993	-2.78079	-3.48374
H	2.83225	2.61141	2.11739
B	1.68176	2.79458	2.35857
O	1.21696	3.38650	3.52232
O	0.63942	2.46938	1.47288
C	-0.16610	3.43900	3.37438
H	-0.82316	4.37577	5.22169
C	-1.11348	3.94979	4.25820
C	-0.52276	2.89369	2.13228
C	-2.45662	3.89240	3.83448
C	-1.84230	2.84898	1.69200
H	-3.23847	4.28204	4.49293
C	-2.81196	3.35909	2.58038
H	-2.10577	2.47489	0.69954
H	-3.86378	3.34834	2.28010
H	2.58939	-0.16891	2.10183

H

BP86 energy = -2682.38620759
Enthalpy 0K = -2681.429874
Enthalpy 298K = -2681.367371
Free energy 298K = -2681.528632
Low freq. = 11.5237 cm-1
Second freq. = 17.5899 cm-1

Zn	0.61763	-0.09678	-0.28420
N	-0.07985	1.05215	-1.69605
N	1.13464	-1.77235	-1.15287
C	0.62915	-1.93800	-2.39753
C	-0.10248	-0.95297	-3.11444
C	-0.34940	0.42046	-2.86491
H	-0.43306	-1.28877	-4.10041
C	0.80068	-3.26848	-3.11370
H	1.65731	-3.84145	-2.73438
H	0.91657	-3.11885	-4.19780
H	-0.10408	-3.88386	-2.96186
C	2.03117	-2.76122	-0.57904
C	1.51567	-3.84766	0.18426
C	3.43875	-2.59537	-0.77360
C	4.30398	-3.53552	-0.18130
C	3.81347	-4.60478	0.57868
C	2.43523	-4.75306	0.75392
C	4.01991	-1.46277	-1.63081
H	5.38374	-3.42988	-0.31933
H	4.50686	-5.32295	1.02761

H	2.05495	-5.59691	1.33908
C	0.01951	-4.11001	0.37743
C	5.42405	-1.00283	-1.18140
H	5.47984	-0.79464	-0.10035
H	5.70648	-0.08376	-1.72195
H	6.19418	-1.75734	-1.41684
C	4.07787	-1.84843	-3.13010
H	3.33505	-0.59568	-1.54337
H	4.69414	-2.75307	-3.27140
H	4.53461	-1.03328	-3.71733
H	3.08181	-2.04633	-3.55279
C	-0.37439	-4.19365	1.86969
H	-1.46956	-4.28481	1.97004
H	-0.04752	-3.30790	2.43587
H	0.07240	-5.07943	2.35322
C	-0.42079	-5.41061	-0.33882
H	-0.53502	-3.26642	-0.07016
H	0.06360	-6.29200	0.11568
H	-0.16050	-5.41062	-1.40932
H	-1.51198	-5.54866	-0.24850
C	-0.96625	1.21603	-4.00033
H	-0.97937	0.63490	-4.93296
H	-0.42113	2.15668	-4.17493
H	-2.00327	1.49785	-3.74851
H	3.87772	4.04787	-1.77922
H	3.59927	2.15665	-4.14482
H	4.50924	2.39074	-1.77940
C	2.66451	2.66879	-3.85880
C	3.62297	3.00195	-1.53747
H	1.84929	2.24605	-4.46817
H	3.44975	2.94946	-0.44988
C	2.39936	2.50898	-2.34213
H	2.27946	1.42846	-2.14226
H	-4.00971	4.01614	-1.76016
H	2.76792	3.73411	-4.12833
C	-3.75861	2.95226	-1.91266
H	-3.49566	2.82907	-2.97688
C	-0.10561	2.50340	-1.64302
C	-2.61773	2.49972	-0.97058
C	1.10214	3.21069	-1.92759
C	-1.29630	3.20067	-1.28940
C	-1.24911	4.60938	-1.22776
C	1.08423	4.61716	-1.85685
C	-0.07658	5.31736	-1.50969
H	-2.15882	5.15830	-0.96186
H	1.99954	5.17376	-2.08274
H	-2.47225	1.41437	-1.10755
H	-0.06758	6.41101	-1.46430
H	-4.67062	2.36485	-1.71362
H	-3.20346	3.80407	0.70299
C	-3.03663	2.73169	0.49989
H	-3.97363	2.19521	0.72247
H	-2.27038	2.37561	1.21041
B	-2.07684	-0.58141	1.53706
C	-0.19121	-0.63865	2.80502
C	-1.28476	-0.29028	3.60703
C	1.05942	-0.92964	3.34315
C	-1.15969	-0.12323	4.98496
C	0.11610	-0.35376	5.53730
C	1.19869	-0.76376	4.73820
O	-2.43429	-0.22167	2.84207
O	-0.62906	-0.73436	1.46267
H	-2.02038	0.14843	5.60091
H	2.16454	-0.98013	5.20301
H	0.25756	-0.23911	6.61572
H	-4.64852	-3.11392	-2.83364
C	-2.83600	-1.38745	-0.84218
C	-5.07839	-1.19589	-0.69704
C	-6.45932	-1.22203	-0.95416
C	-7.30452	-0.77624	0.06888
C	-4.52712	-0.74442	0.53934
C	-3.07462	-0.86834	0.43806
C	-6.78333	-0.32420	1.30511
C	-5.40603	-0.30396	1.55269
C	-4.14984	-2.13002	-2.86174
N	-4.01328	-1.57861	-1.51914
H	-3.14957	-2.24462	-3.30321
H	-6.86296	-1.57827	-1.90671
H	-8.38711	-0.78127	-0.08968
H	-7.47493	0.01214	2.08374
H	-5.01402	0.03800	2.51458
H	-1.89475	-1.63417	-1.33517
H	-4.74239	-1.44996	-3.49603
H	3.55341	-0.62927	1.10232
B	3.14660	0.41063	1.50329
O	3.73268	1.19712	2.46339
O	1.94331	0.99500	1.01319
C	2.88947	2.29620	2.62707
H	3.90784	3.44094	4.16509
C	3.04805	3.37376	3.49444
C	1.79516	2.19958	1.76052
C	2.04363	4.36002	3.45124
C	0.80130	3.16718	1.69091
H	2.12128	5.22643	4.11415
C	0.94974	4.25943	2.57070
H	-0.02786	3.10980	0.98206
H	0.19518	5.05055	2.55528
H	1.87418	-1.32873	2.73308

TS(H-D)

BP86 energy = -2682.37886958
Enthalpy 0K = -2681.422857
Enthalpy 298K = -2681.361108
Free energy 298K = -2681.521304
Low freq. = -21.1227 cm-1

Second freq. = 8.1535 cm-1

Zn	1.02038	0.61533	-0.33563
N	0.35971	2.25027	-1.12955
N	2.26766	-0.20178	-1.55228
C	2.40111	0.46092	-2.73323
C	1.73243	1.66020	-3.07532
C	0.86308	2.51931	-2.36047
H	1.99147	2.03219	-4.06956
C	3.33433	-0.08905	-3.79770
H	4.12081	-0.72877	-3.37434
H	3.80173	0.73176	-4.36207
H	2.75891	-0.69660	-4.51779
C	3.11369	-1.33517	-1.22483
C	2.80582	-2.64004	-1.70712
C	4.24181	-1.10608	-0.37532
C	5.01984	-2.21609	0.00679
C	4.71365	-3.50971	-0.43548
C	3.62471	-3.70929	-1.28764
C	4.65728	0.30351	0.06542
H	5.88717	-2.06561	0.65517
H	5.33284	-4.35708	-0.12475
H	3.40132	-4.71943	-1.64608
C	1.66979	-2.94371	-2.68933
C	5.49289	0.33235	1.36220
H	5.04031	-0.25824	2.17599
H	5.60351	1.37158	1.71369
H	6.51175	-0.05690	1.19398
C	5.43661	1.04110	-1.05227
H	3.73090	0.88814	0.24976
H	6.34269	0.47362	-1.32558
H	5.75404	2.03863	-0.70315
H	4.83414	1.18122	-1.96217
C	0.64120	-3.93873	-2.10480
H	-0.16817	-4.12235	-2.83268
H	0.19383	-3.56031	-1.17473
H	1.10997	-4.91392	-1.88801
C	2.22034	-3.50649	-4.02410
H	1.14261	-1.99603	-2.90440
H	2.66950	-4.50312	-3.87385
H	2.99499	-2.86305	-4.47038
H	1.40335	-3.62236	-4.75690
C	0.50122	3.82145	-3.05011
H	1.31320	4.14226	-3.71913
H	0.28984	4.62268	-2.32705
H	-0.40437	3.68857	-3.66738
H	1.88692	4.83953	2.88769
H	3.58609	4.81617	0.37575
H	3.12626	3.61099	2.58990
C	2.50373	4.91523	0.18489
C	2.05831	3.84473	2.44270
H	2.34355	4.86035	-0.90419
H	1.45949	3.11881	3.01838
C	1.71161	3.81989	0.93944
H	2.05615	2.84551	0.53709
H	-3.98223	4.27693	-2.36980
H	2.18931	5.91831	0.52103
C	-3.15963	3.70684	-2.83436
H	-2.42334	4.43858	-3.20474
C	-0.42454	3.22677	-0.40227
C	-2.54084	2.70089	-1.83270
C	0.21179	3.94430	0.65555
C	-1.80468	3.42028	-0.69772
C	-2.52826	4.33385	0.09664
C	-0.56283	4.84552	1.40973
C	-1.92297	5.03901	1.14095
H	-3.58989	4.49701	-0.11696
H	-0.09079	5.41146	2.21842
H	-1.80972	2.07801	-2.37818
H	-2.50648	5.74546	1.73962
H	-3.58164	3.17267	-3.70276
H	-4.37912	2.31713	-0.68639
C	-3.64465	1.76197	-1.29472
H	-4.19606	1.29733	-2.12941
H	-3.23753	0.94251	-0.67940
B	-1.80946	-2.18282	0.66617
C	0.14520	-2.59537	1.67680
C	-0.90030	-2.96741	2.53910
C	1.48626	-2.81022	1.99729
C	-0.65559	-3.55234	3.77975
C	0.69662	-3.76048	4.12546
C	1.74201	-3.40616	3.25399
O	-2.11631	-2.70006	1.93727
O	-0.39429	-2.04881	0.51333
H	-1.47551	-3.84697	4.43953
H	2.77740	-3.60532	3.54524
H	0.93343	-4.22396	5.08762
H	-4.34436	-2.38442	-4.39332
C	-2.55762	-1.72954	-1.78657
C	-4.80691	-1.75685	-1.64058
C	-6.18461	-1.73051	-1.91685
C	-7.05368	-1.88431	-0.83025
C	-4.28399	-1.92843	-0.32280
C	-2.83041	-1.90644	-0.42472
C	-6.56000	-2.06008	0.48558
C	-5.18671	-2.08370	0.75124
C	-3.82647	-1.51139	-3.96094
N	-3.72123	-1.64145	-2.51372
H	-2.81718	-1.44880	-4.39245
H	-6.56781	-1.60240	-2.93385
H	-8.13424	-1.87338	-1.00268
H	-7.27081	-2.18521	1.30845
H	-4.81286	-2.23102	1.76875
H	-1.59616	-1.69915	-2.30018
H	-4.38567	-0.59943	-4.23187
H	2.88256	-0.10774	2.32435

```
B   1.76142  0.08678  2.65074
O   1.26895  0.10520  3.93097
O   0.75287  0.47931  1.70863
C  -0.07581  0.45401  3.82920
H  -0.72965  0.40574  5.89790
C  -1.00625  0.58693  4.85676
C  -0.41985  0.68685  2.49410
C  -2.30933  0.95995  4.47417
C  -1.69518  1.05980  2.09324
H  -3.07651  1.07427  5.24518
C  -2.64575  1.19111  3.12690
H  -1.94972  1.26694  1.05205
H  -3.66646  1.48322  2.86671
H   2.29126 -2.58522  1.29140
```

TS(F1-I)

```
BP86 energy = -2276.11462465
Enthalpy 0K = -2275.252674
Enthalpy 298K = -2275.198196
Free energy 298K = -2275.343121
Low freq. = -9.3397 cm-1
Second freq. = 11.3293 cm-1

Zn  0.62840 -0.64025 -0.05518
N  -0.14820 -2.43638  0.00603
N   2.49402 -0.97783  0.46172
C   2.88271 -2.24733  0.71830
C   2.03300 -3.37250  0.63159
C   0.65369 -3.47620  0.33944
H   2.51636 -4.32311  0.86590
C   4.31530 -2.50340  1.15159
H   4.52894 -2.00115  2.11065
H   5.03098 -2.09232  0.42092
H   4.50520 -3.57971  1.26951
C   3.44140  0.09657  0.65852
C   3.52026  0.72300  1.93524
C   4.24663  0.53328 -0.42962
C   5.12552  1.61235 -0.21363
C   5.21258  2.24512  1.03166
C   4.41381  1.80062  2.09298
C   4.18065 -0.11849 -1.81296
H   5.74826  1.96524 -1.04283
H   5.90170  3.08382  1.17611
H   4.48327  2.29912  3.06598
C   2.65110  0.28154  3.11645
C   3.62901  0.86162 -2.87393
H   2.64036  1.25900 -2.59290
H   3.53840  0.35678 -3.85174
H   4.30238  1.72660 -3.00324
C   5.55331 -0.67548 -2.25643
H   3.48211 -0.97130 -1.74790
H   6.29528  0.13280 -2.37914
H   5.46139 -1.19066 -3.22832
H   5.96480 -1.39612 -1.52879
C   1.52934  1.30990  3.39605
H   0.89104  0.97230  4.23123
H   0.88464  1.46359  2.51441
H   1.95437  2.29094  3.67254
C   3.48006  0.02035  4.39387
H   2.16623 -0.67103  2.83970
H   3.94299  0.94617  4.77740
H   4.28993 -0.70793  4.21626
H   2.83284 -0.37812  5.19422
C   0.05023 -4.86585  0.45939
H   0.83877 -5.63083  0.50601
H  -0.62640 -5.09280 -0.37824
H  -0.55547 -4.94082  1.37961
H  -2.53377 -2.35946 -4.32326
H  -0.14044 -4.31138 -4.17139
H  -0.84641 -1.96695 -4.71915
C  -0.83000 -4.34110 -3.30971
C  -1.58316 -1.99292 -3.89739
H  -0.38965 -5.00874 -2.55042
H  -1.73769 -0.96285 -3.54119
C  -1.07398 -2.91277 -2.76513
H  -0.10095 -2.51536 -2.42486
H  -3.52884 -3.53455  3.42473
H  -1.77519 -4.79876 -3.65155
C  -2.43066 -3.50049  3.31659
H  -2.09639 -4.50769  3.01346
C  -1.55284 -2.70018 -0.22532
C  -1.99104 -2.41389  2.30958
C  -2.01241 -2.92789 -1.55423
C  -2.44900 -2.71442  0.87966
C  -3.80831 -2.98865  0.63062
C  -3.38084 -3.20253 -1.74324
C  -4.27468 -3.24016 -0.66565
H  -4.51070 -3.00801  1.47117
H  -3.75094 -3.38903 -2.75701
H  -0.88716 -2.39020  2.31145
H  -5.33325 -3.46312 -0.83720
H  -2.01024 -3.28842  4.31486
H  -3.59077 -1.00077  2.82402
C  -2.48824 -1.02198  2.76630
H  -2.09442 -0.77946  3.76904
H  -2.18642 -0.22574  2.06592
B  -1.42617  0.98355 -1.21921
C   0.62319  2.24147 -0.85549
C   0.03046  2.56417 -2.09960
C   1.68312  2.96715 -0.32280
C   0.53976  3.63123 -2.85517
C   1.62826  4.36129 -2.33443
C   2.19006  4.04318 -1.08824
O  -1.01265  1.77245 -2.42434
O  -0.06156  1.15896 -0.27282
```

```
H   2.11828  2.71058  0.64617
H   0.08181  3.88485 -3.81543
H   3.02872  4.62615 -0.69640
H   2.03274  5.19885 -2.91284
H  -1.46358 -0.22095 -1.45633
H  -6.39385  1.09586  1.77566
C  -3.83883  0.73092 -0.24623
C  -4.41363  2.75612  0.55369
C  -5.07735  3.85472  1.12692
C  -4.37742  5.06649  1.19237
C  -3.07414  2.83129  0.04478
C  -2.71392  1.51484 -0.46690
C  -3.05502  5.17111  0.69786
C  -2.40138  4.07132  0.12871
C  -6.18058  0.95852  0.69975
N  -4.85980  1.45366  0.36115
H  -6.22820 -0.11608  0.46871
H  -6.10170  3.77223  1.50633
H  -4.86252  5.94510  1.63115
H  -2.53537  6.13342  0.76070
H  -1.37959  4.17303 -0.25100
H  -4.00075 -0.31986 -0.49264
H  -6.96711  1.47779  0.12134
```

I

```
BP86 energy = -2276.12679370
Enthalpy 0K = -2275.265081
Enthalpy 298K = -2275.210040
Free energy 298K = -2275.355854
Low freq. = 11.3911 cm-1
Second freq. = 15.8877 cm-1
```

```
Zn  0.55812 -0.51657 -0.22673
N  -0.18123 -2.33622 -0.32154
N   2.41117 -0.76414 -0.83806
C   2.75058 -1.97491 -1.33053
C   1.92081 -3.12351 -1.29619
C   0.59981 -3.31506 -0.83018
H   2.38462 -4.02067 -1.71256
C   4.11845 -2.16565 -1.96583
H   4.88269 -2.37249 -1.19704
H   4.44153 -1.26265 -2.50587
H   4.10351 -3.01836 -2.66091
C   3.36323  0.32388 -0.86544
C   4.40877  0.38157  0.10232
C   3.21321  1.35050 -1.84230
C   4.12208  2.42596 -1.82751
C   5.14751  2.50199 -0.87891
C   5.28219  1.48601  0.07332
C   2.13773  1.29826 -2.92987
H   4.02096  3.21768 -2.57750
H   5.84012  3.35022 -0.88193
H   6.08370  1.54870  0.81755
C   4.61279 -0.68978  1.17817
C   1.33637  2.61434 -3.02966
H   0.91807  2.90272 -2.05190
H   0.50452  2.49888 -3.74602
H   1.96549  3.44644 -3.39265
C   2.75275  0.92336 -4.29929
H   1.42601  0.49682 -2.66136
H   3.48804  1.68209 -4.62094
H   1.96795  0.86388 -5.07378
H   3.27010 -0.05051 -4.26435
C   4.28800 -0.13814  2.58427
H   4.39435 -0.93206  3.34508
H   3.26422  0.26361  2.63946
H   4.97698  0.68128  2.85485
C   6.04643 -1.27093  1.16096
H   3.90968 -1.51645  0.97635
H   6.79066 -0.51127  1.45748
H   6.33819 -1.64242  0.16393
H   6.12915 -2.10769  1.87624
C   0.03454 -4.72064 -0.94512
H   0.80662 -5.43110 -1.27327
H  -0.79946 -4.75245 -1.66658
H  -0.37684 -5.06133  0.01915
H  -3.97598 -0.82960 -2.73974
H  -2.43046 -2.96018 -4.45404
H  -2.58889 -0.49429 -3.80727
C  -2.72646 -3.23833 -3.42771
C  -2.87317 -0.78869 -2.78176
H  -2.29561 -4.23030 -3.20826
H  -2.54549  0.00100 -2.08644
C  -2.25480 -2.16057 -2.42440
H  -1.15930 -2.06928 -2.52339
H  -1.64151 -4.79099  3.52489
H  -3.82545 -3.34497 -3.41899
C  -0.70051 -4.46438  3.04804
H  -0.45274 -5.19938  2.26341
C  -1.55104 -2.62477  0.02837
C  -0.82673 -3.03089  2.48157
C  -2.56209 -2.54378 -0.97398
C  -1.88133 -2.94698  1.37601
C  -3.23048 -3.20550  1.69153
C  -3.89390 -2.81019 -0.60221
C  -4.23243 -3.14614  0.71481
H  -3.49550 -3.46570  2.72243
H  -4.67955 -2.75374 -1.36372
H   0.14517 -2.75946  2.03390
H  -5.27282 -3.36650  0.97794
H   0.09383 -4.50730  3.81356
H  -2.07136 -2.25603  4.12628
C  -1.11906 -2.02418  3.61702
H  -0.32090 -2.06481  4.37952
H  -1.17789 -0.99551  3.22757
```

B	-0.76980	1.48420	0.26368
C	0.53399	1.54107	2.19770
C	0.64425	2.76839	1.51030
C	1.05955	1.35569	3.47204
C	1.32232	3.84538	2.08492
C	1.87465	3.66421	3.37242
C	1.74302	2.44796	4.05875
O	0.00903	2.73932	0.29962
O	-0.19356	0.62190	1.43597
H	0.93843	0.40475	3.99833
H	1.40494	4.79612	1.55124
H	2.16843	2.33673	5.06089
H	2.40581	4.49691	3.84488
H	-0.39086	0.80066	-0.84022
H	-6.21723	1.05860	1.90831
C	-3.26808	0.73701	0.74985
C	-4.50571	2.45115	-0.03336
C	-5.54481	3.32370	-0.40165
C	-5.18400	4.52960	-1.01585
C	-3.12243	2.75495	-0.25463
C	-2.33604	1.64291	0.25642
C	-3.82454	4.85472	-1.24559
C	-2.79519	3.98405	-0.87039
C	-5.77519	0.54473	1.03451
N	-4.56425	1.20471	0.58011
H	-5.53128	-0.49107	1.31361
H	-6.59541	3.07697	-0.21485
H	-5.96640	5.23432	-1.31698
H	-3.57728	5.81015	-1.72091
H	-1.74642	4.24977	-1.03244
H	-3.11055	-0.23463	1.22023
H	-6.52722	0.52049	0.22651

TS(I-J)

BP86 energy = -2276.12630972
Enthalpy 0K = -2275.265404
Enthalpy 298K = -2275.211036
Free energy 298K = -2275.355161
Low freq. = -68.9476 cm-1
Second freq. = 10.1559 cm-1

Zn	0.49351	-0.45762	-0.33260
N	-0.26349	-2.27264	-0.39358
N	2.34291	-0.76487	-0.96653
C	2.62736	-1.96071	-1.52350
C	1.76684	-3.08638	-1.49358
C	0.46599	-3.25600	-0.96939
H	2.18181	-3.97930	-1.96692
C	3.95332	-2.15995	-2.24036
H	4.74912	-2.44023	-1.52925
H	4.28031	-1.23747	-2.74404
H	3.86876	-2.96812	-2.98238
C	3.32818	0.29210	-0.97657
C	4.42923	0.25087	-0.07037
C	3.15777	1.39135	-1.86762
C	4.09904	2.43798	-1.82895
C	5.17762	2.41728	-0.93850
C	5.33350	1.33045	-0.07155
C	2.02429	1.45114	-2.89393
H	3.98128	3.28397	-2.51466
H	5.89518	3.24432	-0.92098
H	6.17696	1.31649	0.62740
C	4.66140	-0.89957	0.91472
C	1.23645	2.77712	-2.82086
H	0.86924	2.96968	-1.79977
H	0.36861	2.74315	-3.50204
H	1.85931	3.63641	-3.12655
C	2.55809	1.20341	-4.32468
H	1.31721	0.63363	-2.66423
H	3.28207	1.98468	-4.61646
H	1.73088	1.21966	-5.05593
H	3.06704	0.22796	-4.40770
C	4.41628	-0.44586	2.37072
H	4.54768	-1.29355	3.06692
H	3.40297	-0.03711	2.50582
H	5.13018	0.34412	2.66328
C	6.07959	-1.50428	0.78527
H	3.93147	-1.69663	0.69129
H	6.85358	-0.78385	1.10268
H	6.31722	-1.80400	-0.24962
H	6.17643	-2.39436	1.43104
C	-0.14717	-4.63907	-1.11247
H	0.59935	-5.36614	-1.46288
H	-0.98364	-4.62433	-1.83225
H	-0.56728	-4.99021	-0.15634
H	-4.07567	-0.55390	-2.55297
H	-2.72425	-2.68819	-4.44539
H	-2.73576	-0.24669	-3.68818
C	-2.99299	-2.99314	-3.41921
C	-2.97675	-0.57035	-2.66035
H	-2.61118	-4.01607	-3.25838
H	-2.56585	0.16978	-1.95453
C	-2.41753	-1.98785	-2.39552
H	-1.32488	-1.94910	-2.54704
H	-1.56637	-4.89157	3.35739
H	-4.09502	-3.03865	-3.36805
C	-0.64347	-4.52212	2.87631
H	-0.41087	-5.19975	2.03745
C	-1.62287	-2.54626	0.00784
C	-0.80345	-3.05494	2.41296
C	-2.67287	-2.41797	-0.94826
C	-1.90129	-2.91173	1.35621
C	-3.23986	-3.16690	1.71712
C	-3.99194	-2.68064	-0.53151
C	-4.28014	-3.06104	0.78552

```
H  -3.46584 -3.46291  2.74761
H  -4.80725 -2.58870 -1.25756
H   0.14736 -2.74264  1.94716
H  -5.31130 -3.28099  1.08333
H   0.17430 -4.60589  3.61330
H  -1.98714 -2.40796  4.15649
C  -1.05851 -2.13226  3.62578
H  -0.23019 -2.21934  4.35135
H  -1.13657 -1.07974  3.31139
B  -0.75217  1.55473  0.61621
C   0.67901  1.41660  2.41379
C   0.81149  2.68635  1.81267
C   1.33753  1.09227  3.59672
C   1.63940  3.66255  2.36677
C   2.32420  3.33780  3.55952
C   2.17291  2.08250  4.16746
O   0.00625  2.80132  0.70891
O  -0.20999  0.63006  1.68930
H   1.20137  0.11484  4.06785
H   1.73315  4.64526  1.89661
H   2.70463  1.86013  5.09792
H   2.97587  4.08724  4.02015
H  -0.31139  0.94503 -0.62130
H  -6.27756  1.09659  1.86942
C  -3.26470  0.77671  0.91268
C  -4.45585  2.46679  0.01249
C  -5.47272  3.32493 -0.44154
C  -5.07815  4.52513 -1.04614
C  -3.06331  2.78008 -0.11424
C  -2.30787  1.68352  0.47082
C  -3.70847  4.85927 -1.18287
C  -2.70178  4.00310 -0.72233
C  -5.78402  0.57297  1.02971
N  -4.54815  1.22943  0.64115
H  -5.55700 -0.45955  1.33348
H  -6.53204  3.07235 -0.32561
H  -5.84248  5.21868 -1.41243
H  -3.43541  5.81039 -1.65253
H  -1.64640  4.27710 -0.81190
H  -3.13239 -0.18504  1.40993
H  -6.48556  0.53958  0.17806
```

J

```
BP86 energy = -1467.16525241
Enthalpy 0K = -1466.541016
Enthalpy 298K = -1466.502069
Free energy 298K = -1466.613381
Low freq. = 13.9602 cm-1
Second freq. = 16.5472 cm-1

Zn  0.00001 -0.00091 -0.80138
N  -1.48710 -0.00014  0.51856
N   1.48710  0.00011  0.51864
C   1.28178  0.00066  1.85164
C  -0.00003  0.00082  2.45063
C  -1.28180  0.00058  1.85158
H  -0.00005  0.00127  3.54296
C   2.48209  0.00116  2.78345
H   3.11904  0.88416  2.60398
H   3.11950 -0.88158  2.60432
H   2.16793  0.00129  3.83726
C   2.83047  0.00002 -0.00511
C   3.46906  1.23789 -0.30001
C   3.46899 -1.23791 -0.29981
C   4.74957 -1.20970 -0.88490
C   5.39167 -0.00014 -1.17545
C   4.74964  1.20952 -0.88506
C   2.78335 -2.58085 -0.04066
H   5.24945 -2.15571 -1.12106
H   6.38751 -0.00018 -1.63118
H   5.24960  2.15544 -1.12138
C   2.78324  2.58082 -0.04130
C   2.32084 -3.21969 -1.37140
H   1.64512 -2.54609 -1.92558
H   1.78851 -4.16940 -1.18613
H   3.18437 -3.43631 -2.02502
C   3.67167 -3.55946  0.75838
H   1.88078 -2.38365  0.56364
H   4.57176 -3.85326  0.19040
H   3.11320 -4.48348  0.98868
H   4.00918 -3.11883  1.71216
C   2.31915  3.21823 -1.37219
H   1.78661  4.16789 -1.18732
H   1.64318  2.54384 -1.92512
H   3.18199  3.43453 -2.02683
C   3.67192  3.56060  0.75590
H   1.88134  2.38385  0.56406
H   4.57113  3.85457  0.18660
H   4.01087  3.12092  1.70961
H   3.11314  4.48444  0.98613
C  -2.48213  0.00129  2.78336
H  -2.16798  0.00025  3.83718
H  -3.12038 -0.88067  2.60343
H  -3.11824  0.88508  2.60469
H  -3.18395 -3.43573 -2.02566
H  -3.11332 -4.48379  0.98780
H  -1.78823 -4.16901 -1.18668
C  -3.67186 -3.55980  0.75758
C  -2.32057 -3.21927 -1.37178
H  -4.00970 -3.11945  1.71138
H  -1.64477 -2.54546 -1.92560
C  -2.78345 -2.58086 -0.04095
H  -1.88105 -2.38375  0.56361
H  -4.57093  3.85462  0.18700
H  -4.57174 -3.85361  0.18927
```

```
C  -3.67172  3.56055  0.75624
H  -4.01067  3.12082  1.70993
C  -2.83050 -0.00003 -0.00513
C  -2.78313  2.58078 -0.04106
C  -3.46912 -1.23790 -0.29992
C  -3.46904  1.23790 -0.29984
C  -4.74967  1.20966 -0.88480
C  -4.74974 -1.20956 -0.88489
C  -5.39179  0.00008 -1.17527
H  -5.24958  2.15565 -1.12099
H  -5.24971 -2.15550 -1.12112
H  -1.88121  2.38373  0.56424
H  -6.38767  0.00013 -1.63090
H  -3.11288  4.48433  0.98654
H  -3.18192  3.43462 -2.02654
C  -2.31906  3.21823 -1.37195
H  -1.78646  4.16786 -1.18704
H  -1.64315  2.54383 -1.92492
H   0.00026 -0.00194 -2.33841
```

TS(J-1)

```
BP86 energy = -1873.05962160
Enthalpy 0K = -1872.229262
Enthalpy 298K = -1872.178246
Free energy 298K = -1872.317152
Low freq. = -318.7465 cm-1
Second freq. = 7.7499 cm-1

Zn  0.24202 -0.38436  0.14144
N   2.05643 -0.87065  0.43793
N  -0.85510 -1.93871  0.19478
C  -0.19023 -3.07845  0.51926
C   1.20337 -3.15008  0.77301
C   2.23577 -2.17930  0.75914
H   1.54738 -4.15033  1.04549
C  -0.97985 -4.36226  0.68625
H  -1.81869 -4.42332 -0.02253
H  -1.41160 -4.40873  1.70203
H  -0.33002 -5.24008  0.55858
C  -2.28904 -1.92078  0.00163
C  -2.80151 -1.90928 -1.32955
C  -3.14798 -1.84802  1.13345
C  -4.53765 -1.80263  0.90440
C  -5.06308 -1.82257 -0.39134
C  -4.19975 -1.86768 -1.49397
C  -2.62204 -1.76991  2.56920
H  -5.21795 -1.75031  1.76073
H  -6.14637 -1.79588 -0.54559
H  -4.62244 -1.86991 -2.50249
C  -1.87564 -1.95544 -2.55032
C  -2.81317 -0.34511  3.14290
H  -2.32341  0.42070  2.51530
H  -2.39357 -0.27480  4.16106
H  -3.88351 -0.08252  3.19979
C  -3.27712 -2.81480  3.50082
H  -1.53723 -1.97634  2.55091
H  -4.35308 -2.61630  3.64266
H  -2.80519 -2.78715  4.49739
H  -3.18005 -3.83945  3.10448
C  -2.48848 -1.30703 -3.80871
H  -1.71958 -1.20286 -4.59230
H  -2.91254 -0.30905 -3.60431
H  -3.29451 -1.92866 -4.23494
C  -1.41910 -3.39693 -2.88314
H  -0.96176 -1.37986 -2.28944
H  -2.29268 -4.03884 -3.08994
H  -0.84609 -3.85434 -2.06192
H  -0.77762 -3.39933 -3.78109
C   3.62600 -2.65420  1.13191
H   3.57097 -3.55541  1.75951
H   4.18768 -1.87305  1.66658
H   4.20627 -2.91035  0.22833
H   2.50724  3.12777  3.25712
H   2.54891  0.42036  4.80141
H   1.07915  2.27923  3.88219
C   3.19416  0.48000  3.90839
C   1.74831  2.36655  3.00943
H   3.61346 -0.52270  3.72294
H   1.15454  2.75486  2.16349
C   2.39271  0.99944  2.69013
H   1.57395  0.27538  2.51377
H   6.03517 -0.92144 -2.53799
H   4.03442  1.15606  4.14214
C   5.27875 -1.62439 -2.14952
H   5.69290 -2.07790 -1.23364
C   3.14884  0.07677  0.38682
C   3.92775 -0.91734 -1.88889
C   3.27099  1.03353  1.43605
C   4.02080  0.09320 -0.74024
C   5.00522  1.10026 -0.79553
C   4.28024  2.01038  1.32752
C   5.13742  2.05274  0.22165
H   5.68495  1.13348 -1.65344
H   4.40167  2.74494  2.13040
H   3.19124 -1.69111 -1.61110
H   5.91450  2.82084  0.15753
H   5.15435 -2.42125 -2.90200
H   4.09803  0.55352 -3.51860
C   3.42035 -0.25284 -3.18935
H   3.35607 -0.99304 -4.00506
H   2.41687  0.18706 -3.05431
H  -1.36152  1.29493 -2.93449
H   1.48317  3.11863 -0.37863
H  -4.09491  6.36784  1.19901
H  -5.15158  4.92949  1.20290
```

N	-0.11282	2.26428	-1.49806
C	-1.17628	3.11968	-0.99845
C	-0.94472	4.42555	-0.52271
C	-2.48816	2.59575	-0.91456
C	-3.53376	3.37125	-0.40315
C	-2.00427	5.18790	-0.00412
C	-3.31747	4.68734	0.06002
C	-0.37453	1.77125	-2.87547
C	1.24217	2.85012	-1.41874
C	-4.45893	5.52731	0.58723
H	0.05183	4.87098	-0.56323
H	-2.69630	1.57172	-1.24491
H	1.97477	2.10195	-1.75568
H	0.39143	1.02737	-3.14756
H	-1.79794	6.20320	0.35214
H	-4.53971	2.93992	-0.35364
H	-0.34023	2.60128	-3.60853
H	1.34481	3.75203	-2.05487
H	-5.05084	5.95521	-0.24229
H	-0.60205	1.06955	0.90721
H	-0.41353	1.34262	0.16492

K

BP86 energy = -1869.52312133
Enthalpy 0K = -1868.749132
Enthalpy 298K = -1868.701378
Free energy 298K = -1868.831203
Low freq. = 15.1934 cm-1
Second freq. = 17.4697 cm-1

Zn	0.06100	-0.30116	-0.15709
N	-1.41137	-1.29638	0.60056
N	1.61746	-1.13473	0.63716
C	1.44055	-2.18431	1.47689
C	0.17525	-2.71726	1.81265
C	-1.13426	-2.32557	1.43657
H	0.21092	-3.57179	2.49139
C	2.66381	-2.82859	2.09725
H	3.19991	-2.11070	2.74183
H	3.37881	-3.14724	1.32038
H	2.38996	-3.70262	2.70421
C	2.92538	-0.60547	0.33513
C	3.45565	0.43969	1.14531
C	3.60507	-1.04926	-0.83680
C	4.79252	-0.38531	-1.20775
C	5.30692	0.67169	-0.44456
C	4.64691	1.06656	0.72725
C	3.10285	-2.22345	-1.68234
H	5.33021	-0.71318	-2.10413
H	6.23480	1.16752	-0.74764
H	5.07176	1.86963	1.33932
C	2.77275	0.89082	2.43956
C	2.72961	-1.79000	-3.11879
C	4.13874	-3.37180	-1.72469
H	5.06483	-3.06121	-2.23821
H	3.72916	-4.23821	-2.27099
H	4.41845	-3.70932	-0.71281
C	2.10964	2.27804	2.27971
H	1.60079	2.57790	3.21147
H	1.35671	2.28016	1.47060
H	2.86392	3.05092	2.04739
C	3.74506	0.89186	3.64093
H	1.96846	0.16943	2.66478
H	4.53861	1.65018	3.52665
H	4.23746	-0.08666	3.76817
H	3.20082	1.12349	4.57200
C	-2.28472	-3.10997	2.03701
H	-3.07167	-3.30553	1.29205
H	-2.75741	-2.53655	2.85402
H	-1.93467	-4.06548	2.45262
H	-3.59031	-1.69677	-3.79426
H	-2.67610	-4.37779	-2.57437
H	-1.96488	-2.38156	-3.99614
C	-3.23002	-3.70829	-1.89421
C	-2.58151	-1.73473	-3.34913
H	-3.20799	-4.15584	-0.88699
H	-2.17002	-0.71080	-3.39202
C	-2.61415	-2.28856	-1.90655
H	-1.56957	-2.38246	-1.55567
H	-4.73638	0.82783	3.63376
H	-4.28204	-3.68401	-2.22684
C	-3.82652	0.20793	3.70737
H	-4.14887	-0.84334	3.79515
C	-2.77597	-0.92707	0.29321
C	-2.88566	0.42409	2.50078
C	-3.34789	-1.35540	-0.93935
C	-3.49030	-0.07904	1.18693
C	-4.78696	0.32895	0.81825
C	-4.65226	-0.92117	-1.25186
C	-5.36885	-0.08536	-0.38628
H	-5.35237	0.98137	1.49182
H	-5.11633	-1.25239	-2.18690
H	-1.96295	-0.15050	2.69367
H	-6.38216	0.23638	-0.64707
H	-3.31570	0.48800	4.64414
H	-3.36611	2.54663	2.19222
C	-2.48371	1.91346	2.38848
H	-2.01932	2.26210	3.32706
H	-1.76913	2.08337	1.56433
H	-0.21341	0.27259	-2.50017
H	3.34381	3.23260	-1.28516
C	2.41827	3.81793	-1.19964
H	2.37251	4.27300	-0.19815
H	2.40377	4.61185	-1.96340
H	2.29546	1.13948	-1.90650

```
N   1.27142  2.92664 -1.39098
C   1.33364  1.62247 -1.72259
H   0.04672  5.45113 -0.75230
C  -0.07716  3.32275 -1.25219
C   0.01740  1.05105 -1.75776
C  -0.59778  4.59105 -0.95389
C  -0.89479  2.18759 -1.50893
C  -1.99341  4.70917 -0.94363
H  -2.44609  5.68089 -0.72606
C  -2.29496  2.33177 -1.49029
C  -2.82619  3.59869 -1.21520
H  -2.95219  1.47716 -1.67804
H  -3.91190  3.73140 -1.20493
H   3.60232 -1.38067 -3.65656
H   1.94108 -1.01640 -3.12745
H   2.35694 -2.65130 -3.69865
H   2.18895 -2.61620 -1.20305
```

TS(K-L)

```
BP86 energy = -2275.03456758
Enthalpy 0K = -2274.066873
Enthalpy 298K = -2274.007795
Free energy 298K = -2274.162354
Low freq. = -895.5869 cm-1
Second freq. = 11.9062 cm-1

Zn -0.77137  0.33481 -0.34570
N  -0.55012  2.21235 -0.88778
N  -2.68074  0.06843 -0.83960
C  -3.39499  1.07810 -1.38572
C  -2.84526  2.33338 -1.73035
C  -1.57518  2.89009 -1.45624
H  -3.54966  3.02280 -2.20284
C  -4.89197  0.92547 -1.60414
H  -5.44029  1.36352 -0.75087
H  -5.20250 -0.12536 -1.68971
H  -5.20890  1.47216 -2.50593
C  -3.32699 -1.14155 -0.39900
C  -3.96070 -1.18323  0.87720
C  -3.22920 -2.31576 -1.20744
C  -3.75353 -3.52145 -0.70069
C  -4.35991 -3.58073  0.56226
C  -4.46391 -2.41858  1.33461
C  -2.60632 -2.26855 -2.60674
H  -3.69545 -4.43067 -1.30643
H  -4.76521 -4.52802  0.93261
H  -4.95543 -2.46433  2.31269
C  -4.10807  0.05387  1.76798
C  -1.99791 -3.61212 -3.05894
H  -1.32818 -4.05201 -2.29968
H  -1.42255 -3.47476 -3.99023
H  -2.77986 -4.35923 -3.27854
C  -3.62039 -1.79056 -3.67538
H  -1.79836 -1.50966 -2.57284
H  -4.49374 -2.46473 -3.70801
H  -3.15347 -1.79122 -4.67553
H  -3.98221 -0.77131 -3.47654
C  -3.23708 -0.05437  3.04084
H  -3.34629  0.85048  3.66273
H  -2.16819 -0.16886  2.79288
H  -3.54082 -0.91999  3.65608
C  -5.58390  0.31576  2.14859
H  -3.74876  0.92854  1.19943
H  -5.98552 -0.48485  2.79356
H  -6.23203  0.37937  1.25894
H  -5.67290  1.26315  2.70697
C  -1.41751  4.36705 -1.77994
H  -2.04364  4.64033 -2.64301
H  -0.37404  4.64435 -1.98573
H  -1.75458  4.97856 -0.92405
H   3.87723  2.10881 -3.30568
H   1.28783  2.33955 -4.77879
H   2.98962  0.71268 -3.92328
C   1.29367  2.90910 -3.83302
C   3.11618  1.35678 -3.03496
H   0.27650  3.29584 -3.66954
H   3.52738  0.74608 -2.21332
C   1.77093  2.01374 -2.66354
H   1.01551  1.21079 -2.54482
H  -0.02881  5.76736  2.52785
H   1.97070  3.77150 -3.96100
C  -0.82759  5.30276  1.92445
H  -0.93853  5.90326  1.00608
C   0.65814  2.91229 -0.51524
C  -0.51734  3.81994  1.61449
C   1.81572  2.78248 -1.33935
C   0.69713  3.66411  0.69419
C   1.91414  4.27305  1.06019
C   3.00312  3.41776 -0.92554
C   3.05817  4.15625  0.26404
H   1.96131  4.85258  1.98830
H   3.90067  3.33831 -1.54595
H  -1.39569  3.39698  1.09679
H   3.99105  4.64426  0.56432
H  -1.76368  5.38457  2.50280
H   0.53796  3.40258  3.49781
C  -0.33019  3.02703  2.92890
H  -1.22239  3.12915  3.57087
H  -0.15902  1.95461  2.73822
H   0.04789 -3.86053  3.90174
C  -0.45939 -1.98192  1.32775
H  -1.27911 -2.57556  0.91589
H  -1.52618 -3.63481  3.06590
C  -0.69515 -3.11787  3.56588
N  -0.09220 -2.17318  2.62868
```

```
C   1.21590 -0.51149  1.77344
C   0.94463 -1.28168  2.94364
C   2.23097  0.46744  1.83470
C   1.64101 -1.11883  4.15361
C   2.92837  0.64433  3.03573
H   1.41603 -1.72286  5.03783
C   2.64052 -0.13823  4.17960
H  -1.08627 -2.58108  4.44561
H   3.71028  1.40756  3.09380
H   3.20506  0.02688  5.10237
H   2.46243  1.08582  0.96129
C   0.32711 -0.98207  0.68524
H   1.80328 -1.04126 -2.65350
C   1.44283 -2.05340 -2.42416
H   1.81123 -2.76404 -3.18198
H   0.34254 -2.06197 -2.43424
H   7.81789 -0.96953  0.49963
C   3.80709 -2.35486  0.50682
C   7.59268 -1.94503  0.03229
C   3.34147 -2.28782 -0.81920
C   5.17695 -2.24902  0.77044
C   4.25627 -2.11834 -1.86996
C   6.11655 -2.07433 -0.26876
C   5.62821 -2.01088 -1.58671
N   1.89163 -2.43773 -1.06009
H   1.18672 -1.68115 -0.28700
H   3.10447 -2.47242  1.33679
H   5.52194 -2.29793  1.80872
H   3.92696 -2.07440 -2.91061
H   6.33093 -1.88178 -2.41678
H   7.92931 -2.72485  0.73603
H   8.20133 -2.02439 -0.88195
C   1.43337 -3.82443 -0.73450
H   0.33539 -3.86165 -0.80373
H   1.87877 -4.54315 -1.44142
H   1.74321 -4.07961  0.28741
```

L

BP86 energy = -2275.04687005
Enthalpy 0K = -2274.075194
Enthalpy 298K = -2274.014566
Free energy 298K = -2274.178459
Low freq. = 3.8135 cm-1
Second freq. = 8.4276 cm-1

```
Zn -1.36071  0.41305  0.01047
N  -0.84413  2.30617 -0.12920
N  -3.12879  0.55626 -0.87979
C  -3.61038  1.72205 -1.35296
C  -2.91158  2.95088 -1.25834
C  -1.65036  3.23730 -0.69299
H  -3.43377  3.81182 -1.68084
C  -4.97363  1.74568 -2.01906
H  -5.76383  1.45491 -1.30580
H  -5.01988  1.01916 -2.84769
H  -5.20930  2.74511 -2.41130
C  -3.90740 -0.65397 -0.97344
C  -4.80178 -0.99712  0.08214
C  -3.69301 -1.54124 -2.06740
C  -4.37634 -2.77329 -2.07576
C  -5.24936 -3.12980 -1.04016
C  -5.45718 -2.24299  0.02414
C  -2.74065 -1.20097 -3.21612
H  -4.22346 -3.46296 -2.91351
H  -5.77497 -4.09004 -1.06802
H  -6.14721 -2.51973  0.82893
C  -5.05046 -0.07139  1.27581
C  -1.52171 -2.15263 -3.23236
H  -0.98542 -2.12938 -2.26782
H  -0.81707 -1.86759 -4.03353
H  -1.83476 -3.19541 -3.41849
C  -3.45649 -1.20950 -4.58589
H  -2.36227 -0.17832 -3.04325
H  -3.83214 -2.21563 -4.84124
H  -2.76199 -0.90139 -5.38655
H  -4.31785 -0.52078 -4.60069
C  -4.45157 -0.66169  2.57406
H  -4.60407  0.02950  3.42108
H  -3.36901 -0.84801  2.47088
H  -4.93574 -1.62004  2.83311
C  -6.55126  0.24516  1.46631
H  -4.53111  0.88181  1.07565
H  -7.12765 -0.65794  1.73214
H  -7.00171  0.66811  0.55237
H  -6.68823  0.97544  2.28215
C  -1.18627  4.68225 -0.72724
H  -1.94227  5.33107 -1.19147
H  -0.24330  4.78451 -1.29120
H  -0.98185  5.05391  0.29142
H   3.09441  0.86164 -1.98941
H   1.99975  3.16697 -3.89310
H   1.88749  0.66856 -3.29316
C   2.17608  3.39210 -2.82733
C   2.01758  0.92801 -2.22804
H   1.80354  4.41108 -2.62966
H   1.48361  0.17571 -1.62066
C   1.47399  2.34561 -1.93462
H   0.40265  2.34395 -2.20029
H   0.07259  4.58233  4.13001
H   3.26888  3.40165 -2.66955
C  -0.77348  4.40657  3.44305
H  -0.80188  5.24657  2.72851
C   0.43795  2.68670  0.40614
C  -0.65215  3.04357  2.72411
C   1.58278  2.67013 -0.44260
```

```
C   0.56078  2.99313  1.79253
C   1.84586  3.25279  2.30961
C   2.84351  2.94074  0.12517
C   2.98206  3.22623  1.49069
H   1.95390  3.48995  3.37366
H   3.72858  2.94240 -0.52122
H  -1.55439  2.91384  2.10162
H   3.96809  3.44945  1.91249
H  -1.69831  4.44036  4.04386
H   0.26439  1.95714  4.40374
C  -0.62271  1.88667  3.74979
H  -1.51748  1.92040  4.39498
H  -0.59531  0.90256  3.25199
H  -0.43525 -5.50809  1.52807
C  -1.33836 -2.41377  0.81672
H  -2.30861 -2.65696  0.37926
H  -2.09569 -4.89945  1.26517
C  -1.16634 -4.73819  1.82970
N  -0.66522 -3.40112  1.53605
C   0.57052 -1.49040  1.54823
C   0.51239 -2.85663  2.01272
C   1.69166 -0.71243  1.91880
C   1.50774 -3.43230  2.82546
C   2.69734 -1.28302  2.72496
H   1.41911 -4.45733  3.20091
C   2.59930 -2.62743  3.18463
H  -1.38166 -4.85510  2.90611
H   3.52410 -0.65712  3.08065
H   3.36803 -3.02706  3.85415
H   1.75821  0.33803  1.61211
C  -0.63140 -1.21396  0.78148
H   2.47493 -1.81298 -1.08906
C   2.95124 -2.76830 -0.83364
H   3.37252 -3.24516 -1.72927
H   2.22213 -3.42471 -0.33932
H   7.86788  1.33882 -3.11385
C   5.18829 -0.26332  0.03012
C   8.11851  1.06177 -2.07430
C   5.11081 -1.58076 -0.43427
C   6.17158  0.58521 -0.50360
C   5.98099 -2.05681 -1.42626
C   7.06905  0.14130 -1.49485
C   6.95202 -1.19229 -1.94638
N   4.07275 -2.48557  0.14165
H   3.62486 -1.98418  0.96021
H   4.49060  0.10919  0.78683
H   6.24080  1.61350 -0.13453
H   5.91921 -3.08504 -1.79635
H   7.63577 -1.56347 -2.71691
H   8.20880  1.99196 -1.49259
H   9.10802  0.57544 -2.09991
C   4.65600 -3.75942  0.70399
H   3.85879 -4.28126  1.25046
H   5.03469 -4.37956 -0.11946
H   5.47516 -3.49517  1.38579
```

TS(L-M)

```
BP86 energy = -2276.11787970
Enthalpy 0K = -2275.255976
Enthalpy 298K = -2275.201275
Free energy 298K = -2275.347434
Low freq. = -143.2855 cm-1
Second freq. = 7.3241 cm-1

Zn  0.18260 -0.67020 -0.20500
N  -1.01124 -2.18509 -0.66234
N   1.94544 -1.57050 -0.52469
C   2.00589 -2.86310 -0.89908
C   0.87138 -3.65717 -1.18754
C  -0.50648 -3.38062 -1.03669
H   1.09910 -4.67754 -1.50695
C   3.34802 -3.57726 -0.97082
H   3.54974 -4.09150 -0.01375
H   4.18230 -2.88495 -1.15229
H   3.33564 -4.34660 -1.75867
C   3.14218 -0.85192 -0.16901
C   3.65792 -0.94633  1.15663
C   3.74344  0.01700 -1.12942
C   4.85329  0.78606 -0.72786
C   5.36192  0.71389  0.57576
C   4.76666 -0.14871  1.50335
C   3.23908  0.09021 -2.57444
H   5.32553  1.45728 -1.45189
H   6.22470  1.32406  0.86409
H   5.17028 -0.21031  2.52053
C   3.04958 -1.87430  2.21229
C   3.31828  1.50564 -3.18278
H   2.79830  2.24599 -2.55607
H   2.83764  1.50707 -4.17629
H   4.36349  1.83071 -3.33340
C   3.99508 -0.91195 -3.48142
H   2.17568 -0.21089 -2.56348
H   5.07604 -0.68429 -3.49669
H   3.62087 -0.84627 -4.51815
H   3.87339 -1.95406 -3.14651
C   2.39580 -1.07708  3.36418
H   1.94276 -1.76260  4.10171
H   1.60747 -0.40404  2.98936
H   3.14441 -0.46209  3.89516
C   4.09259 -2.86910  2.77237
H   2.25272 -2.46271  1.72563
H   4.88642 -2.34722  3.33524
H   4.58215 -3.44591  1.96975
H   3.61194 -3.58241  3.46448
C  -1.44525 -4.55677 -1.26081
```

```
H  -0.99406 -5.28625 -1.95055
H  -2.42243 -4.24101 -1.65371
H  -1.63550 -5.07623 -0.30445
H  -4.30464  0.17025 -3.60541
H  -2.56431 -1.97986 -4.85975
H  -2.71082  0.47086 -4.32446
C  -2.97349 -2.25218 -3.87095
C  -3.22406  0.21792 -3.38040
H  -2.52224 -3.21284 -3.57437
H  -3.04663  1.03706 -2.66697
C  -2.69157 -1.12787 -2.84420
H  -1.59510 -1.03836 -2.74216
H  -3.69309 -4.24106  2.92778
H  -4.06056 -2.41086 -3.98720
C  -2.71023 -4.37083  2.44172
H  -2.84335 -5.09031  1.61592
C  -2.43136 -2.04680 -0.43795
C  -2.14023 -3.02084  1.94816
C  -3.24971 -1.48679 -1.46329
C  -2.98508 -2.41746  0.82202
C  -4.36106 -2.20604  1.03603
C  -4.62126 -1.30679 -1.19541
C  -5.17760 -1.65661  0.04112
H  -4.79783 -2.47770  2.00353
H  -5.26372 -0.88121 -1.97307
H  -1.12973 -3.21235  1.54722
H  -6.24582 -1.50233  0.22741
H  -2.03288 -4.82468  3.18611
H  -2.97259 -1.81145  3.58425
C  -1.99093 -2.03845  3.13256
H  -1.35149 -2.47686  3.91922
H  -1.54762 -1.08123  2.81421
H   1.41575  4.36442  2.99828
C   1.08937  1.70550  1.24904
H   2.12637  1.68922  0.90954
H   2.69391  3.12686  2.77021
C   1.66580  3.29602  3.12137
N   0.75973  2.45994  2.34791
C  -1.14677  1.51458  1.53489
C  -0.62044  2.36474  2.55509
C  -2.53748  1.27631  1.51822
C  -1.41567  2.96505  3.54429
C  -3.34202  1.86780  2.50048
H  -0.98662  3.61646  4.31259
C  -2.79087  2.70047  3.50272
H   1.60962  3.03857  4.19301
H  -4.42154  1.68454  2.49174
H  -3.44773  3.14992  4.25466
H  -2.98014  0.64231  0.74419
C  -0.03813  1.09379  0.66516
B  -0.22109  1.74870 -1.34752
C  -1.34775  3.67702 -1.24488
C   0.03001  3.97208 -1.32462
C  -2.30319  4.68065 -1.09729
C   0.50167  5.28274 -1.26215
C  -0.46121  6.30628 -1.11444
C  -1.83309  6.01128 -1.03296
O   0.74987  2.81016 -1.48977
O  -1.54380  2.31866 -1.35826
H  -3.36772  4.43966 -1.03243
H   1.57115  5.50162 -1.33309
H  -0.12858  7.34820 -1.06345
H  -2.55469  6.82631 -0.91646
H  -0.03292  0.74749 -2.01278
```

M

BP86 energy = -2276.12545646
Enthalpy 0K = -2275.263082
Enthalpy 298K = -2275.208439
Free energy 298K = -2275.352319
Low freq. = 14.8314 cm-1
Second freq. = 16.1371 cm-1

```
Zn  0.13163 -0.05839 -0.61153
N  -1.01222 -1.45526 -1.45460
N   1.91853 -0.57629 -1.34044
C   1.95957 -1.41802 -2.39486
C   0.82857 -2.05336 -2.95578
C  -0.50520 -2.15652 -2.48726
H   1.04472 -2.66371 -3.83690
C   3.29533 -1.77030 -3.03647
H   3.66531 -2.73805 -2.65453
H   4.06784 -1.01487 -2.83400
H   3.17259 -1.87782 -4.12606
C   3.12660 -0.16180 -0.68186
C   3.81307 -1.07176  0.17607
C   3.56697  1.19050 -0.81958
C   4.68051  1.60733 -0.06411
C   5.35163  0.72989  0.79914
C   4.92066 -0.59705  0.90805
C   2.87648  2.15863 -1.78605
H   5.02802  2.64060 -0.15232
H   6.21506  1.07929  1.37550
H   5.45552 -1.28554  1.57235
C   3.38691 -2.53424  0.34491
C   3.04579  3.64130 -1.40365
H   2.73086  3.82645 -0.36546
H   2.41320  4.26339 -2.05847
H   4.08745  3.98620 -1.53554
C   3.35436  1.93486 -3.24180
H   1.79344  1.93604 -1.75817
H   4.44366  2.09890 -3.32520
H   2.85164  2.64641 -3.91980
H   3.13232  0.91804 -3.60074
C   2.78590 -2.79725  1.74396
```

```
H   2.46845 -3.85059  1.83729
H   1.90733 -2.15870  1.93166
H   3.53075 -2.60153  2.53621
C   4.55449 -3.51150  0.07489
H   2.59348 -2.74758 -0.39052
H   5.34618 -3.41876  0.83906
H   5.02257 -3.33218 -0.90745
H   4.19540 -4.55505  0.09995
C  -1.36851 -3.18178 -3.20801
H  -1.09946 -3.22283 -4.27524
H  -2.44203 -2.96747 -3.11172
H  -1.19216 -4.18765 -2.78715
H  -5.00561  1.47838 -2.14587
H  -3.42585  0.61315 -4.59699
H  -3.61805  2.37777 -2.79187
C  -3.60185 -0.19293 -3.86316
C  -3.90757  1.58297 -2.08312
H  -3.03970 -1.08046 -4.19631
H  -3.63775  1.91576 -1.06949
C  -3.18406  0.26959 -2.44577
H  -2.10043  0.48327 -2.47059
H  -2.75445 -5.56864  0.54663
H  -4.67702 -0.44553 -3.88967
C  -1.86801 -5.21662 -0.00981
H  -2.08806 -5.33041 -1.08454
C  -2.35709 -1.70725 -0.99292
C  -1.51723 -3.75608  0.36070
C  -3.40933 -0.84214 -1.41647
C  -2.60838 -2.78135 -0.09148
C  -3.92026 -2.95522  0.39118
C  -4.70244 -1.06882 -0.90592
C  -4.96159 -2.10931 -0.00523
H  -4.12572 -3.77328  1.09066
H  -5.52167 -0.41614 -1.22454
H  -0.58502 -3.48883 -0.16673
H  -5.97489 -2.26344  0.38097
H  -1.03076 -5.89069  0.24300
H  -2.13864 -3.90848  2.46443
C  -1.24164 -3.64665  1.87673
H  -0.43412 -4.34022  2.17165
H  -0.94697 -2.62553  2.16464
H   2.26973  0.60739  4.99184
C   1.21200  0.82396  2.00111
H   2.13799  1.25662  1.61980
H   3.27307  0.28314  3.53525
C   2.36490 -0.00985  4.08133
N   1.21216  0.17539  3.21021
C  -0.93390  0.21627  2.43788
C  -0.09789 -0.20886  3.51562
C  -2.32400 -0.01141  2.53003
C  -0.59325 -0.86277  4.65655
C  -2.82770 -0.65487  3.66648
H   0.06534 -1.17876  5.47178
C  -1.97597 -1.07912  4.71449
H   2.45754 -1.06852  4.37563
H  -3.90458 -0.83320  3.74833
H  -2.40395 -1.57949  5.58922
H  -2.98844  0.32355  1.72965
C  -0.08125  0.88204  1.44684
B  -0.53254  2.01525  0.36892
C  -1.89034  3.82431  0.39576
C  -0.55417  4.27812  0.30956
C  -2.96347  4.71395  0.37894
C  -0.25540  5.63623  0.20388
C  -1.34012  6.54386  0.18313
C  -2.66612  6.09251  0.26928
O   0.32105  3.22164  0.37296
O  -1.93109  2.45526  0.51350
H  -3.99323  4.35269  0.45506
H   0.78148  5.97970  0.14352
H  -1.13649  7.61642  0.09857
H  -3.48795  6.81580  0.25191
H  -0.43517  1.55104 -0.92438
```

TS(M-J)

BP86 energy = -2276.12473089
Enthalpy 0K = -2275.263314
Enthalpy 298K = -2275.209327
Free energy 298K = -2275.350736
Low freq. = -59.4025 cm-1
Second freq. = 17.5657 cm-1

```
Zn  0.06419  0.05639 -0.73196
N  -0.95790 -1.46014 -1.53066
N   1.88294 -0.35600 -1.44806
C   1.96615 -1.15124 -2.53707
C   0.87753 -1.85631 -3.09877
C  -0.42991 -2.08461 -2.60140
H   1.11898 -2.40957 -4.01025
C   3.30676 -1.35622 -3.23054
H   3.76670 -2.30955 -2.91717
H   4.02004 -0.55058 -3.00536
H   3.15808 -1.41175 -4.32091
C   3.07155  0.11517 -0.79206
C   3.86187 -0.78817 -0.02022
C   3.39450  1.50575 -0.85008
C   4.49699  1.96536 -0.10386
C   5.27074  1.09281  0.67419
C   4.95419 -0.26989  0.70532
C   2.59375  2.47139 -1.72961
H   4.75530  3.02767 -0.13268
H   6.12427  1.47494  1.24442
H   5.56889 -0.95311  1.30251
C   3.56634 -2.28993  0.06576
C   2.67425  3.93976 -1.27154
```

H	2.39818	4.04542	-0.21115
H	1.96907	4.54756	-1.86282
H	3.68122	4.36719	-1.42829
C	3.01745	2.36020	-3.21462
H	1.53086	2.16942	-1.66800
H	4.08641	2.61142	-3.33533
H	2.43197	3.06440	-3.83117
H	2.85544	1.34844	-3.61744
C	3.04867	-2.69321	1.46464
H	2.81151	-3.77122	1.49233
H	2.13820	-2.13510	1.73642
H	3.81425	-2.50068	2.23753
C	4.80020	-3.14456	-0.30751
H	2.76432	-2.52504	-0.65335
H	5.60855	-3.03480	0.43670
H	5.21676	-2.86326	-1.28908
H	4.52987	-4.21430	-0.34181
C	-1.23982	-3.14129	-3.33635
H	-0.92754	-3.19898	-4.39045
H	-2.32036	-2.94315	-3.28667
H	-1.06938	-4.13507	-2.88559
H	-5.09098	1.26454	-1.80736
H	-3.74169	0.59625	-4.47785
H	-3.80314	2.24787	-2.53727
C	-3.83803	-0.25326	-3.77901
C	-3.99561	1.39878	-1.85905
H	-3.30026	-1.11169	-4.21496
H	-3.62983	1.66964	-0.85679
C	-3.28996	0.13058	-2.38345
H	-2.21852	0.37194	-2.50098
H	-2.30024	-5.76228	0.41645
H	-4.90782	-0.52212	-3.71993
C	-1.46768	-5.32960	-0.16568
H	-1.71723	-5.44870	-1.23367
C	-2.26476	-1.83478	-1.04476
C	-1.22470	-3.85021	0.21525
C	-3.39417	-1.04384	-1.40688
C	-2.40497	-2.95895	-0.18074
C	-3.68551	-3.25811	0.32364
C	-4.65187	-1.39612	-0.87969
C	-4.80301	-2.48814	-0.01706
H	-3.80526	-4.11486	0.99616
H	-5.52907	-0.80100	-1.15519
H	-0.33636	-3.50046	-0.33889
H	-5.79018	-2.74072	0.38456
H	-0.56860	-5.93456	0.04606
H	-1.75997	-4.07722	2.33527
C	-0.90758	-3.73596	1.72264
H	-0.03580	-4.36270	1.98177
H	-0.68948	-2.69708	2.01551
H	2.62055	0.38603	4.96182
C	1.26632	0.82466	2.14558
H	2.13393	1.34605	1.73898

H	3.49144	0.17855	3.40210
C	2.64482	-0.17348	4.00941
N	1.41388	0.02164	3.25780
C	-0.79516	0.05301	2.69448
C	0.15874	-0.46270	3.62818
C	-2.16029	-0.26078	2.87787
C	-0.20301	-1.28062	4.71282
C	-2.52818	-1.07101	3.95786
H	0.54076	-1.66069	5.42052
C	-1.56357	-1.57769	4.86246
H	2.79401	-1.24349	4.22988
H	-3.58409	-1.31675	4.11047
H	-1.88575	-2.20729	5.69837
H	-2.90994	0.13948	2.19034
C	-0.07157	0.88365	1.73472
B	-0.66934	1.99357	0.77055
C	-2.17618	3.66088	0.65777
C	-0.88816	4.21753	0.49946
C	-3.32584	4.44513	0.57818
C	-0.70912	5.57890	0.25587
C	-1.87047	6.37996	0.17097
C	-3.15119	5.82538	0.32919
O	0.07330	3.24580	0.65348
O	-2.08500	2.31039	0.91096
H	-4.31804	4.00471	0.71134
H	0.29225	6.00376	0.14239
H	-1.76514	7.45278	-0.02051
H	-4.03298	6.47052	0.25972
H	-0.62509	1.53242	-0.66145

S11. References

[1] Ihara, E.; Young, J.; Jordan, R. F. Cationic Aluminum Alkyl Complexes Incorporating Aminotroponiminate Ligands. *J. Am. Chem. Soc.* **1998**, *120*, 8277–8278.

[2] Stender, M.; Wright, R. J.; Eichler, B. E.; Prust, J.; Olmstead, M. M.; Roesky, H. W.; Power, P. P. The Synthesis and Structure of Lithium Derivatives of the Sterically Encumbered *β*-Diketiminate Ligand [{(2,6-$Pr^i_2H_3C_6$)N(CH_3)C}$_2$CH]$^-$, and a Modified Synthesis of the Aminoimine Precursor. *J. Chem. Soc. Dalt. Trans.* **2001**, 3465–3469.

[3] Eberhardt, R.; Allmendinger, M.; Luinstra, G. A.; Rieger, B. The Ethylsulfinate Ligand: A Highly Efficient Initiating Group for the Zinc *β*-Diiminate Catalyzed Copolymerization Reaction of CO_2 and Epoxides. *Organometallics* **2003**, *22*, 211–214.

[4] Garçon, M.; Mun, N. W.; White, A. J. P.; Crimmin, M. R. Palladium-Catalysed C-H Bond Zincation of Arenes: Scope, Mechanism, and the Role of Heterometallic Intermediates. *Angew. Chem., Int. Ed.* **2021**, *60*, 6145–6153.

[5] Gaussian 09, Revision E.01, Frisch, M. J.; Trucks, G. W.; Schlegel, H. B.; Scuseria, G. E.; Robb, M. A.; Cheeseman, J. R.; Scalmani, G.; Barone, V.; Mennucci, B.; Petersson, G. A.; Nakatsuji, H.; Caricato, M.; Li, X.; Hratchian, H. P.; Izmaylov, A. F.; Bloino, J.; Zheng, G.; Sonnenberg, J. L.; Hada, M.; Ehara, M.; Toyota, K.; Fukuda, R.; Hasegawa, J.; Ishida, M.; Nakajima, T.; Honda, Y.; Kitao, O.; Nakai, H.; Vreven, T.; Montgomery, J. A., Jr.; Peralta, J. E.; Ogliaro, F.; Bearpark, M.; Heyd, J. J.; Brothers, E.; Kudin, K. N.; Staroverov, V. N.; Kobayashi, R.; Normand, J.; Raghavachari, K.; Rendell, A.; Burant, J. C.; Iyengar, S. S.; Tomasi, J.; Cossi, M.; Rega, N.; Millam, J. M.; Klene, M.; Knox, J. E.; Cross, J. B.; Bakken, V.; Adamo, C.; Jaramillo, J.; Gomperts, R.; Stratmann, R. E.; Yazyev, O.; Austin, A. J.; Cammi, R.; Pomelli, C.; Ochterski, J. W.; Martin, R. L.; Morokuma, K.; Zakrzewski, V. G.; Voth, G. A.; Salvador, P.; Dannenberg, J. J.; Dapprich, S.; Daniels, A. D.; Farkas, Ö.; Foresman, J. B.; Ortiz, J. V.; Cioslowski, J.; Fox, D. J. Gaussian, Inc., Wallingford CT, **2013**.

[6] (a) Becke, A. D. Density-functional thermochemistry. III. The role of exact exchange. *J. Chem. Phys.* **1993**, *98*, 5648-5652; (b) Burke, K; Perdew, J. P. ; Yang, W. Electronic Density Functional Theory: Recent Progress and New Directions, (Ed: J. F. Dobson; G. Vignale, M. P. Das), Springer, Heildelberg, **1998**.

[7] Mennucci B.; Cancès E.; Tomasi J. Evaluation of Solvent Effects in Isotropic and Anisotropic Dielectrics and in Ionic Solutions with a Unified Integral Equation Method: Theoretical Bases, Computational Implementation, and Numerical Applications. *J. Phys. Chem. B* **1997**, *101*, 10506-10517.

[8] Granados, A.; Shafir, A.; Arrieta, A.; Cossío, F. P.; Vallribera, A. Stepwise Mechanism for the Bromination of Arenes by a Hypervalent Iodine Reagent. *J. Org. Chem.* **2020**, *85*, 4, 2142–2150.

[9] Tanaka, S.; Saito, Y.; Yamamoto, T.; Hattori, T. Electrophilic Borylation of Terminal Alkenes with BBr_3/2,6-

Disubstituted Pyridines. *Org. Lett.* 2018, **20**, 1828–1831.

[10]Britton, L.; Docherty, J. H.; Dominey, A. P.; Thomas, S. P. Iron-Catalysed C(sp^2)-H Borylation Enabled by Carboxylate Activation. *Mol.* **2020**, *25*, 905–915.

[11] Varni, A. J.; Bautista, M. V.; Noonan, K. J. T. Chemoselective Rhodium-Catalyzed Borylation of Bromoiodoarenes Under Mild Conditions. *J. Org. Chem.* **2020**, *85*, 6770–6777.

[12]Rochette, É.; Desrosiers, V.; Soltani, Y.; Fontaine, F. G. Isodesmic C–H Borylation: Perspectives and Proof of Concept of Transfer Borylation Catalysis. *J. Am. Chem. Soc.* **2019**, *141*, 12305–12311.

[13] Yin, Q.; Klare, H. F. T.; Oestreich, M. Catalytic Friedel–Crafts C−H Borylation of Electron-Rich Arenes: Dramatic Rate Acceleration by Added Alkenes. *Angew. Chem., Int. Ed.*, **2017**, *56*, 3712–3717.

[14] Del Grosso, A.; Singleton, P. J.; Muryn, C. A.; Ingleson, M. J. Pinacol Boronates by Direct Arene Borylation with Borenium Cations. *Angew. Chem., Int. Ed.* **2011**, *50*, 2102–2106.

[15] Grundy, M. E.; Yuan, K.; Nichol, G. S.; Ingleson, M. J. Zinc catalysed electrophilic C–H borylation of heteroarenes. *Chem. Sci.* **2021**, *12*, 8190-8198.

[16] Slack, E. D.; Colacot, T. J. Understanding the Activation of Air-Stable Ir(COD)(Phen)Cl Precatalyst for C–H Borylation of Aromatics and Heteroaromatics. *Org. Lett.* **2021**, *23*, 1561–1565.

[17] Lehmann, M.; Schulz, A.; Villinger, A. Bissilylated Halonium Ions: [Me_3Si-X-$SiMe_3$][$B(C_6F_5)_4$] (X = F, Cl, Br, I). *Angew. Chem., Int. Ed.* **2009**, *48*, 7444– 7447.

[18] Frisch, M. J.; Trucks, G. W.; Schlegel, H. B.; Scuseria, G. E.; Robb, M. A.; Cheeseman, J. R.; Scalmani, G.; Barone, V.; Petersson, G. A.; Nakatsuji, H.; Li, X.; Caricato, M.; Marenich, A. V.; Bloino, J.; Janesko, B. G.; Gomperts, R.; Mennucci, B.; Hratchian, H. P.; Ortiz, J. V.; Izmaylov, A. F.; Sonnenberg, J. L.; Williams-Young, D.; F. Ding, F. L.; F. Egidi, J. G.; B. Peng, A. P.; Henderson, T.; Ranasinghe, D.; Zakrzewski, V. G.; Gao, J.; Rega, N.; Zheng, G.; Liang, W.; Hada, M.; Ehara, M.; Toyota, K.; Fukuda, R.; Hasegawa, J.; Ishida, M.; Nakajima, T.; Honda, Y.; Kitao, O.; Nakai, H.; Vreven, T.; Throssell, K.; J. A. Montgomery, J.; Peralta, J. E.; Ogliaro, F.; Bearpark, M. J.; Heyd, J. J.; Brothers, E. N.; Kudin, K. N.; Staroverov, V. N.; Keith, T. A.; Kobayashi, R.; Normand, J.; Raghavachari, K.; Rendell, A. P.; Burant, J. C.; Iyengar, S. S.; Tomasi, J.; Cossi, M.; Millam, J. M.; Klene, M.; Adamo, C.; Cammi, R.; Ochterski, J. W.; Martin, R. L.; Morokuma, K.; Farkas, O.; Foresman, J. B.; Fox, D. J. *Gaussian 16, Revision A.03* Gaussian Inc.: Wallingford CT, 2016.

[19] Becke, A. D. Density-functional exchange-energy approximation with correct asymptotic behavior. *Phys. Rev. A* **1988**, *38*, 3098–3100.

[20] Perdew, J. P. Density-functional approximation for the correlation energy of the inhomogeneous electron gas. *Phys. Rev. B* **1986**, *33*, 8822–8824.

[21] Andrae, D.; Häußermann, U.; Dolg, M.; Stoll, H.; Preuß, H. Energy-adjustedab initio pseudopotentials for the second and third row transition elements. *Theor. Chim. Acta* **1990**, *77*, 123–141.

[22] Hehre, W. J.; Ditchfield, R.; Pople, J. A. Self-Consistent Molecular Orbital Methods. XII. Further Extensions of Gaussian-Type Basis Sets for Use in Molecular Orbital Studies of Organic Molecules. *J. Chem. Phys,* **1972**, *56*, 2257-2261.

[23] Hariharan, P. C.; Pople, J. A. The influence of polarization functions on molecular orbital hydrogenation energies. *Theor. Chim. Acta* **1973**, *28*, 213–222.

[24] Weigend, F.; Ahlrichs, R. Balanced basis sets of split valence, triple zeta valence and quadruple zeta valence quality for H to Rn: Design and assessment of accuracy. *Phys. Chem. Chem. Phys.* **2005**, *7*, 3297–3305.

[25] Weigend, F.; Köhn, A.; Hättig, C. Efficient use of the correlation consistent basis sets in resolution of the identity MP2 calculations. *J. Chem. Phys,* **2002**, *116*, 3175–3183.

[26] Grimme, S.; Ehrlich, S.; Goerigk, L. Effect of the damping function in dispersion corrected density functional theory. *J. Comput. Chem.* **2011**, *32*, 1456–1465.

[27] Tomasi, J.; Mennucci, B.; Cammi, R. Quantum mechanical continuum solvation models. *Chem. Rev.* **2005**, *105*, 2999–3093.
